# Exploring the health complications of female genital mutilation through a systematic review and meta-analysis

**DOI:** 10.1186/s12889-025-21584-z

**Published:** 2025-04-14

**Authors:** Christina Pallitto, Fernando Ruiz-Vallejo, Vernon Mochache, Karin Stein, Joshua P. Vogel, Max Petzold

**Affiliations:** 1 Department of Sexual and Reproductive Health and Research, and the UNDP/UNFPA/UNICEF/WHO/World Bank Special Programme of Research, Development and Research Training in Human Reproduction (HRP), World Health Organization, 20 Avenue Appia, Geneva, 1211 Switzerland; 2 Facultad de Medicina, Universidad Nacional de Colombia, Bogota, Colombia; 3 Division of Healthier Populations, World Health Organization, Geneva, Switzerland; 4 Burnet Institute, Melbourne, VIC Australia; 5 School of Population and Global Health, University of Melbourne, Parkville, VIC Australia; 6 School of Public Health and Community Medicine, Institute of Medicine, University of Gothenburg, Gothenburg, Sweden

**Keywords:** Female genital mutilation, FGM, FGC, Health burden, Sexual health, Reproductive health, Mental health

## Abstract

**Background:**

Female genital mutilation (FGM) is a harmful practice that affects an estimated 230 million women and girls. Previous research indicates that FGM is associated with increased risk of short- and long-term health complications. Understanding the health complications is important in ensuring high quality care for women and girls already affected and for advocating for prevention of the practice.

**Objective:**

The objective of this study was to conduct a systematic review and meta-analysis of all existing evidence on the association between FGM and a range of health complications.

**Methods:**

We conducted a systematic review of the literature on the health complications of FGM published between February 2009 and December 2022, applying search strategies and terms aligned with previous reviews. We identified studies that compared women with various types of FGM versus those without for six domains of health complications (i.e., immediate, obstetric & neonatal, gynecological, urological, sexual and mental). Random effects meta-analysis was conducted by health condition and FGM type. Immediate health complications were analysed separately based on data from population-based surveys.

**Results:**

We analysed data from 78 studies (*n* = 486,949), of which 67 informed the meta-analyses comparing women with and without FGM and 11 informed analyses on the immediate health complications. Most of the studies (*N* = 68) were conducted in high FGM prevalence countries. Among women and girls living with FGM compared to those without, we found an increased risk for obstetric complications, including prolonged/obstructed labor, obstetric tears, caesarean birth, postpartum hemorrhage, episiotomy, fetal distress, extended maternal hospital stay, neonatal asphyxia, and stillbirth/neonatal death; gynecological complications, including genital tissue damage, genitourinary tract infections, and menstrual difficulties; urological complications, including urinary tract infections and difficulty urinating; sexual complications including dyspareunia and sexual dysfunction; and mental health complications including depression or anxiety and somatoform disorder.

**Conclusion:**

These results support results from previous research finding an association between FGM and a range of health complications over the life course. This calls for strengthening health systems to provide high-quality care for women and girls at-risk of or affected by FGM and ensuring that FGM prevention and care services are included in essential health service packages.

**Supplementary Information:**

The online version contains supplementary material available at 10.1186/s12889-025-21584-z.

## Introduction

Female genital mutilation (FGM) is a harmful practice that involves partial or total removal of external female genitalia or other injury to the female genital organs for non-medical reasons. It is considered a violation of the rights of girls and women and can have a profound impact on their health and well-being, requiring a comprehensive public health response [1]. The practice of FGM has been classified into four types, which relate to the extent of genital tissue affected [2]. Type I involves the partial or total removal of the clitoral glans (the external part of the clitoris) and/or the prepuce. Type II involves the partial or total removal of the clitoral glans and the labia minora, with or without excision of the labia majora. Type III, also known as infibulation, includes the narrowing of the vaginal orifice with the creation of a covering seal by cutting and appositioning the labia minora and/or the labia majora, with or without excision of the clitoral glans. Type IV includes other harmful procedures to the female genitalia for non- medical purposes without excision of genital tissue, for example pricking, piercing, incising, scraping, and cauterizing.

It is estimated that 230 million women and girls alive today have undergone FGM [3]. While population-based data from 31 countries show promising declines among the 15–19-year-old age group, absolute numbers could increase as young population cohorts reach the age at which FGM occurs. The deep-rooted nature of the practice and the fact that it is a social norm driven by cultural beliefs that foster a sense of cultural identity make the abandonment of the practice elusive despite decades of efforts at the community, national and international levels. A large body of evidence exists documenting the ways that different types of FGM can affect multiple domains of health and well-being of women and girls [4–6]. These studies have shown that women who have undergone FGM are at a greater risk of a range of health complications that affect their gynecological, urological, obstetric, sexual, and mental health as compared to women who have not undergone FGM. When this body of evidence is considered as a whole, the breadth and magnitude of the health consequences are clearly seen.

Previous systematic reviews compiling the evidence from available studies have described the range of health complications that can result from the practice [7, 8]. Some studies considered the obstetric and neonatal complications associated with FGM [5], the psychological, social, and sexual complications associated with FGM and a range of “physical” health complications of FGM ranging from short-term to long term complications [6]. The authors of these reviews summarize some of the potential pathways through which health complications occur due to infections, anatomical changes and scarring affecting physiological functions on gynecological, urological, sexual, and obstetric health, as well as the psychological sequelae of a traumatic practice.

The present study updates and expands previous reviews, compiling all available data on health complications from studies with comparison groups of women with and without FGM, by types of FGM, and synthesizing these data using meta-analyses. The result of this process is a comprehensive summary of health complications associated with FGM. Understanding the risk of health complications of FGM is a critical step in the development of guidance and training tools for health workers to support them in providing the necessary prevention and care services to women and girls affected by FGM or at-risk of this harmful practice.

## Methods

We conducted a systematic review of available literature for published data on the health complications of FGM. This review was carried out in accordance with the Preferred Reporting Items for Systematic Reviews & Meta-Analyses (PRISMA) standards [9]. This study builds on the work of Berg et al. who have conducted two systematic reviews of the physical health complications of FGM [6] and the psychological, social, and sexual consequences of FGM [4]. The search strategies for these two reviews are similar. These existing reviews obviate the need to conduct systematic searches of the literature prior to the earlier search date (i.e., Feb 2009). All studies included in Berg 2010 and Berg 2014 reviews were assessed against inclusion criteria described below. A systematic search for studies published from Feb 2009 to Dec 2022 was conducted and studies were assessed against inclusion criteria.

### Eligibility criteria

For all searches, we considered for inclusion, studies with any design that allowed comparison of women exposed to FGM and those not exposed, including cohort studies, case–control studies, cross-sectional studies, but excluding studies where interventions had taken place. For immediate health complications, different criteria were applied and studies without comparison groups (i.e., population-based studies) were analyzed separately to arrive at the proportion of girls reported by their mothers to have undergone FGM having a particular health complication after the practice. Case studies, and studies with fewer than 2 participants were excluded. We assessed study design based on described features (as defined in the Cochrane glossary^1^), not necessarily how they were labelled in the publication, however methodological study quality was not the basis for inclusion.

Studies were excluded from the current analysis if they were qualitative studies; if the outcome was not defined; if the study was a randomized controlled trial testing intervention strategies since the purpose of this analysis was to assess associations independent of interventions; if the results were only available in letter format or conference abstracts without relevant data presented; if the results were from a case report; if studies did not report a reference group of women without FGM (with the exception of immediate health outcomes, which were analyzed differently as described below); if the population studied were recruited in the context of testing an intervention whether for medically indicated or cosmetic reasons; if the population studied captured data on someone other than the person being interviewed (i.e. sexual partners or babies) with the exception of the immediate health outcomes where the data relate to girls as reported by their mothers.

Papers that had contradictions in reported information were also excluded. Systematic reviews or literature reviews were not themselves eligible. However, reference lists of such reviews were assessed to identify potentially eligible studies that had not already been identified.

For the immediate health complications, a targeted search of Demographic and Health Surveys (DHS) was conducted to identify studies that included questions on mothers reporting health complications experienced by their daughters following FGM.

### Study population

The population of interest included girls and women who had undergone any type of FGM. No limitations on age, race/ethnicity, nationality, setting or other participant characteristics were applied. Where reported, this information was extracted and is summarized in Table 1.

**Table 1:** Studies included in the meta-analyses and outcomes analyzed

Study Authors	Country	Sampling	FGM measurement method	FGM Type(s) reported	Sample size: by FGM Type	Sample size: No FGM	Outcomes analyzed	Quality score^a^	Q1	Q2	Q3	Q4	Q5	Q6
Abdel-Aleem et al., 2016 [69]	Egypt	Referral level (public or private)	Clinical report based on medical records or physical examination	Any FGM	Any FGM: 376	54	Dyspareunia	3	1	1	-1	0	1	1
Abdullah, 2021 [86]	Iraq	Referral level (public or private)	Both self-reported and clinically reported	Any FGM	Any FGM: 145	145	Anxiety, Somatoform, Depression	3	1	1	-1	0	1	1
Adinma, 1997 [46]	Nigeria	Referral level (public or private)	Clinical report based on medical records or physical examination	Any FGM	Any FGM: 124	132	Episiotomy	0	-1	1	-1	1	1	-1
Ahmed et al., 2017 [88]	Egypt	Referral level (public or private)	Self-report	Any FGM	Any FGM: 135	69	Anxiety, Depression	5	1	1	1	0	1	1
Akpak et al., 2022 [49]	Sudan	Referral level (public or private)	Clinical report based on medical records or physical examination	Type I, II and III	110	110	Caesarean, Postpartum hemorrhage, Extended hospital stay, Episiotomy, Perineal tears,	4	1	1	-1	1	1	1
Almroth et al., 2005	Sudan	Referral level (public or private)	Both self-reported and clinically reported	Type I and II Type III	Type I: 19 Type II: 9 Type III: 243	7	Infertility	4	1	1	-1	1	1	1
Alsibiani & Rouzi, 2010	Saudi Arabia	Referral level (public or private)	Self-report	Any FGM	Any FGM: 130	130	Sexual dysfunction measured with FSFI	1	1	1	-1	0	1	-1
Andro et al., 2014 [35]	France	Referral level (public or private)	Both self-reported and clinically reported	Any FGM	Any FGM: 678	1706	Caesarean, Instrumental delivery, Obstetric tears/lacerations, Episiotomy, Genitourinary tract infections, Pregnancy loss, Urinary incontinence, Dyspareunia	4	1	1	1	1	1	-1
Anikwe et al. 2019 [15]	Nigeria	Referral level (public or private)	Clinical report based on medical records or physical examination	Any FGM Type I and II Type III	Any FGM: 248 Type I and II: 242 Type III: 6	248	Preterm delivery, Caesarean, Prolonged labor, Episiotomy, Obstetric tears/lacerations, Postpartum hemorrhage, Low birthweight	6	1	1	1	1	1	1
Anis et al., 2012 [75]	Egypt	Referral level (public or private)	Both self-reported and clinically reported	Any FGM	Any FGM: 333	317	Sexual dysfunction measured with the FSFI	3	1	1	-1	0	1	1
Applebaum et al., 2008 [84]	Israel	Not clearly described	Self-report	Any FGM	Any FGM: 19	18	PTSD	-1	-1	1	-1	0	1	-1
Arafa et al. 2018 [63]	Egypt	Community-based	Self-report	Any FGM	Any FGM: 815	908	Menstrual difficulties, difficulties urinating	3	1	1	1	0	1	-1
Ashry et al., 2018 [64]	Egypt	Community-based	Self-report	Any FGM	Any FGM: 1846	1507	Menstrual difficulties, difficulties urinating	1	1	1	-1	0	1	-1
Balachandran et al. 2018 [41]	United Kingdom of Great Britain and Northern Ireland	Referral level (public or private)	Clinical report based on medical records or physical examination	Any FGM	Any FGM: 121	121	Instrumental delivery, Episiotomy, Obstetric tear/lacerations, Low birthweight, Caesarean, Postpartum hemorrhage	6	1	1	1	1	1	1
Behrendt and Moritz, 2005 [83]	Senegal	Not clearly described	Self-report	Any FGM	Any FGM:23	24	Anxiety, PTSD	-1	-1	1	-1	0	1	-1
Benin DHS 2001	Benin	Community-based	Self-reported for others (e.g., children)	Any FGM Type I and II Type IV	Any FGM: 246 Type I and II:194 Type IV :13	-	Excessive bleeding, Wound healing problems	1	1	1	1	0	-1	-1
Benin DHS 2006	Benin	Community-based	Self-reported for others (e.g., children)	Any FGM	Any FGM 240	-	Excessive bleeding, Wound healing problems, Difficulties urinating/urinary retention, Swelling in genital area	1	1	1	1	0	-1	-1
Berardi et al., 1985 [43]	France	Referral level (public or private)	Clinical report based on medical records or physical examination	Any FGM (Type II)	Any FGM: 71	781	Fetal Distress, Caesarean, Episiotomy, Instrumental delivery, Obstetric tears/lacerations	1	1	0	-1	1	1	-1
Biglu et al., 2016 [80]	Iran	Referral level (public or private)	Both self-reported and clinically reported	Type I/II	Type I: 49, Type II:107	29	Sexual dysfunction measured with the FSFI	3	1	1	-1	0	1	1
Birge et al., 2017 [82]	Sudan	Referral level (public or private)	Clinical report based on medical records or physical examination	Type III	Type III: 140	140	Sexual dysfunction measured with the FSFI	-1	-1	1	-1	0	1	-1
Boghossian et al., 2019 [48]	Australia	Referral level (public or private)	Clinical report based on medical records or physical examination	Any FGM	Any FGM: 89	89	Pre-term delivery, Low birthweight, Episiotomy	4	1	1	1	1	1	-1
Bohoussou et al., 1996	Côte d'Ivoire	Referral level (public or private)	Clinical report based on medical records or physical examination	Any FGM	Any FGM: 1099	3836	Caesarian, Instrumental delivery, Obstetric tears/lacerations	0	-1	1	-1	1	1	-1
Brewer et al., 2007 [58]	Kenya	Community-based	Self-report	Any FGM	Any FGM: 2153	2153	STI, including HIV	0	1	1	-1	1	-1	-1
Burkina Faso DHS 2003	Burkina Faso	Community-based	Self-reported for others (e.g., children)	Any FGM Type I and II Type III	Any FGM 2380 Type I and II: 2227 Type III: 85	-	Excessive bleeding, Wound healing problems, Difficulties urinating/urinary retention, Swelling in genital area	1	1	1	1	0	-1	-1
Chad DHS 2004	Chad	Community-based	Self-reported for others (e.g., children)	Any FGM Type I and II Type III Type IV	Any FGM: 807 Type I and II:586 Type III: 32 Type IV:177	-	Difficulties urinating/urinary retention, Excessive bleeding, Infections, including fever (systemic; urinary; reproductive tract), Wound healing problems	1	1	1	1	0	-1	-1
Chibber et al., 2011 [24]	Not specified	Referral level (public or private)	Clinical report based on medical records or physical examination	Any FGM	Any FGM: 1842	2958	Caesarean, Extended maternal hospital stay, Fetal Distress, Resuscitation of the newborn, Genitourinary tract infection, Postpartum hemorrhage, Pre-term delivery, Prolonged labour	2	1	1	-1	1	1	-1
Chu et al., 2015	United States of America	Community-based	Self-report	Any FGM	Any FGM: 46	22	Genitourinary tract infections, Menstrual difficulties	1	1	1	0	1	1	-1
Davis and Jellins., 2019 [44]	Australia	Referral level (public or private)	Clinical report based on medical records or physical examination	Any FGM	Any FGM: 141	8421	Instrumental delivery, Caesarean, Episiotomy, Obstetric tear/lacerations	4	1	1	-1	1	1	1
De Silva., 1989 [19]	Saudi Arabia	Primary care	Clinical report based on medical records or physical examination	Any FGM	Any FGM: 167	1990	Caesarean, Instrumental delivery, Genitourinary tract infection, Postpartum hemorrhage, Prolonged labour; Obstetric tear/laceration, STIs, UTI	-2	1	-1	-1	1	-1	-1
Daneshkhah et al., 2017 [79]	Islamic Republic of Iran	Primary care	Self-report	Any FGM	Any FGM: 140	60	Sexual dysfunction, measured with the FSFI	2	1	-1	1	1	1	-1
El-Defrawi et al. 2001 [65]	Egypt	Primary care	Clinical report based on medical records or physical examination	Any FGM	Any FGM: 200	50	Menstrual difficulties, Dyspareunia	3	1	1	1	0	1	-1
Elnashar and Abdelhady, 2007 [33]	Egypt	Referral level (public or private)	Self-report	Any FGM	Any FGM: 200	64	Infertility, Caesarean, Episiotomy, Obstetric tears/lacerations, Pregnancy loss, Menstrual problems, Dyspareunia, Urinary difficulties	1	1	1	-1	0	1	-1
Essén et al., 2005 [20]	Sweden	Referral level (public or private)	Clinical report based on medical records or physical examination	Any FGM	Any FGM: 68	2486	Instrumental delivery, Prolonged labour, Low birthweight	3	1	0	-1	1	1	1
Frega et al., 2013 [14]	Burkina Faso	Referral level (public or private)	Clinical report based on medical records or physical examination	Type I and II	Any FGM: 123 (deliveries) Type I and II: 85	110 (deliveries) 95	Caesarean, Episiotomy, Prolonged labour, Resuscitation of the newborn, Pregnancy loss	2	1	1	-1	1	1	-1
Gebremichael et al., 2018 [22]	Ethiopia	Referral level (public or private)	Clinical report based on medical records or physical examination	Any FGM	Any FGM: 142	139	Caesarian, Prolonged labour, Postpartum hemorrhage	4	1	1	-1	1	1	1
Gudu and Abdulahi, 2017 [21]	Ethiopia	Referral level (public or private)	Self-report	Any FGM	Any FGM: 264	24	Caesarean, Prolonged labour, Instrumental delivery, Obstetric tears/lacerations, Prolonged labour, Episiotomy	3	1	1	-1	0	1	1
Guinea DHS 1999	Guinea	Community-based	Self-reported for others (e.g., children)	Any FGM Type I Type II Type IV	Any FGM:2305 Type I :1539 Type II :628 Type IV :110	-	Difficulties urinating/urinary retention, Excessive bleeding, Infections, including fever (systemic; urinary; reproductive tract)	-3	1	-1	-1	0	-1	-1
Guinea DHS 2005	Guinea	Community-based	Self-reported for others (e.g., children)	Any FGM Type I and II Type III Type IV	Any FGM:2825 Type I and II:2410 Type III :294 Type IV:57	-	Excessive bleeding, Wound healing problems, Difficulties urinating/urinary retention	-3	1	-1	-1	0	-1	-1
Hakim, 2001 [29]	Ethiopia	Referral level (public or private)	Both self-reported and clinically reported	Any FGM	Any FGM: 1225	256	Episiotomy, Pregnancy loss, Obstetric tears/lacerations, Postpartum hemorrhage, Urinary incontinence	2	1	1	-1	1	1	-1
Holmgren et al. 2003 [61]	Guinea-Bissau	Community-based	Self-report	Any FGM	Any FGM: 799	58	STI, including HIV	4	1	1	1	1	1	-1
Ibrahim et al., 2013 [72]	Egypt	Referral level (public or private)	Self-report	Any FGM	Any FGM: 365	144	Sexual dysfunction measured with the FSFI	3	1	1	-1	0	1	1
Johnson-Agbakwu et al., 2022	United States of America	Community-based	Self-report	Any FGM	Any FGM: 687	161	Dyspareunia, Sexual dysfunction, Infertility, Stillbirth, Fetal distress, Caesarean, Neonatal resuscitation, Perineal tears, Postpartum hemorrhage, Extended hospital stay, Depression, PTSD, Menstrual difficulties, Difficulty urinating, UTI, RTI, Genital complications, Fistula	3	1	1	-1	0	1	1
Kanki et al., 1992 [60]	Senegal	Community-based	Self-report	Any FGM	Any FGM: 276	1434	STI, including HIV	2	1	1	-1	1	1	-1
Kaplan et al., 2013 [13]	The Gambia	Referral level (public or private)	Clinical report based on medical records or physical examination	Type I and II	Type I: 326 Type II: 105	139	Genital tissue damage, Menstrual difficulties, Fetal distress, Pregnancy loss, Caesarean, Episiotomy, Dyspareunia, Obstetric tears/lacerations, Prolonged labour, UTI	3	1	1	-1	0	1	1
Kasim et al. 2012 [18]	Egypt	Primary care	Self-report	Any FGM	Any FGM: 200	200	Dyspareunia, Genitourinary tract infections, UTI, Infertility, Prolonged labour, Postpartum hemorrhage	3	1	1	-1	0	1	1
Kizilhan, 2011 [87]	Iraq	Primary care	Self-report	Any FGM	Any FGM: 79	30	Anxiety, depression, PTSD, Somatoform disorder	5	1	1	1	0	1	1
Laleh et al., 2022 [78]	Islamic Republic of Iran	Primary care	Not available	Any FGM	Any FGM: 281	269	Sexual dysfunction	3	1	1	-1	0	1	1
Larsen, 2002	Sudan	Community-based	Self-report	Type I Type II and III	Type I: 4679 Type II and III: 33,510	4572	Infertility	5	1	1	0	1	1	1
Larsen and Okonofua, 2002 [12]	Nigeria	Referral level (public or private)	Both self-reported and clinically reported	Type I and II	Type I: 590 Type II: 202	827	Pregnancy loss, Episiotomy, Prolonged labour, Pregnancy loss, Obstetric tears/lacerations	3	1	1	1	0	1	-1
Larsen and Yan, 2000 [50]	Côte d’Ivoire, United Republic of Tanzania and Central African Republic	Community-based	Self-report	Any FGM	Any FGM CAR: 15,593 Côte d’Ivoire: 18,076 Tanzania: 8033	CAR: 15,615 Côte d’Ivoire: 19,891 Tanzania: 33,463	Infertility	3	1	1	1	0	1	-1
Maheu-Giroux et al., 2016 [68]	Multi-country study	Community-based	Self-report	Any FGM	Any FGM: 102,746	140,618	Fistula	5	1	1	1	0	1	1
Mahmoud, 2016 [45]	Egypt	Primary care	Self-report	Any FGM	Any FGM: 272	272	Menstrual difficulties, Dyspareunia, Prolonged labour, Postpartum hemorrhage	3	1	1	1	0	1	-1
Mahmoudi & Hosseini, 2017 [76]	Islamic Republic of Iran	Primary care	Self-report	Any FGM	Any FGM: 92	87	Sexual dysfunction measured with the FSFI	3	1	1	-1	0	1	1f
Mali DHS 2001	Mali	Community-based	Self-reported for others (e.g., children)	Any FGM Type I and II Type III Type IV	Any FGM:5999 Type I and II:5219 Type III :272 Type IV :137	-	Excessive bleeding, Wound healing problems	0	1	-1	1	0	1	-1
Mali DHS 2006	Mali	Community-based	Self-reported for others (e.g., children)	Any FGM Type I and II Type III Type IV	Any FGM:6256 Type I and II:4860 Type III :996 Type IV :234	-	Excessive bleeding, Wound healing problems, Difficulties urinating/urinary retention	-1	1	-1	1	0	-1	-1
Maslovskaya et al., 2009 [56]	Kenya	Community-based	Self-report	Any FGM	Any FGM: 962	2152	STI, including HIV	6	1	1	1	1	1	1
Matanda et al., 2019 [67]	Burkina Faso	Community-based	Self-report	Any FGM	Any FGM: 12,940	4062	Fistula	3	1	1	-1	0	1	1
	Chad	Community-based	Self-report	Any FGM	Any FGM: 4427	4830		3	1	1	-1	0	1	1
	Côte d’Ivoire	Community-based	Self-report	Any FGM	Any FGM: 3813	5530		3	1	1	-1	0	1	1
	Ethiopia	Community-based	Self-report	Any FGM	Any FGM: 2303	822		3	1	1	-1	0	1	1
	Guinea	Community-based	Self-report	Any FGM	Any FGM: 8852	267		3	1	1	-1	0	1	1
	Kenya	Community-based	Self-report	Any FGM	Any FGM: 3066	11,086		3	1	1	-1	0	1	1
	Mali	Community-based	Self-report	Any FGM	Any FGM: 9531	712		3	1	1	-1	0	1	1
	Nigeria	Community-based	Self-report	Any FGM	Any FGM: 9876	10,383		3	1	1	-1	0	1	1
	Senegal	Community-based	Self-report	Any FGM	Any FGM: 4025	10,296		3	1	1	-1	0	1	1
	Sierra Leone	Community-based	Self-report	Any FGM	Any FGM: 14,816	1694		3	1	1	-1	0	1	1
Mauritania DHS 2001	Mauritania	Community-based	Self-reported for others (e.g., children)	Any FGM Type I and II Type IV	Any FGM 2574 Type I and II: 2073 Type IV: 380	-	Excessive bleeding, Wound healing problems, Difficulties urinating/urinary retention	4	1	1	1	1	1	-1
Millogo-Traore et al., 2007 [23]	Burkina Faso	Referral level (public or private)	Clinical report based on medical records or physical examination	Any FGM	Any FGM: 227	227	Prolonged labour, Episiotomy, Postpartum hemorrhage, Instrumental delivery, Obstetric tears/lacerations, Prolonged labour, Pregnancy loss	2	1	1	-1	1	1	-1
Minsart et al., 2015 [38]	Djibouti	Referral level (public or private)	Both self-reported and clinically reported	Type I and II Type III	Type I and II: 376 Type III: 238	29	Obstetric tears/lacerations, Low birthweight, Caesarean, Preterm delivery, Fetal distress, Pregnancy loss, Instrumental delivery	2	-1	1	-1	1	1	1
Morison et al., 2001 [34]	The Gambia	Referral level (public or private)	Both self-reported and clinically reported	Any FGM	Any FGM: 668	489	Pregnancy loss, Genitourinary infection, STI, Infertility, Menstrual difficulties, Dyspareunia	6	1	1	1	1	1	1
Msuya et al., 2002 [57]	United Repuclic of Tanzania	Primary care	Clinical report based on medical records or physical examination	Any FGM	Any FGM:63	316	Genitourinary tract infections, STIs	6	1	1	1	1	1	1
Ndiaye et al., 2010 [31]	Burkina Faso	Referral level (public or private)	Clinical report based on medical records or physical examination	Any FGM	Any FGM: 210	144	Caesarean, Episiotomy, Postpartum hemorrhage, Resuscitation of the newborn, Obstetric tears/lacerations, Pregnancy loss	2	1	1	-1	1	1	-1
Nonterah et al., 2019	Ghana	Referral level (public or private)	Clinical report based on medical records or physical examination	Any FGM	Any FGM: 1647	7659	Stillbirth, Low birth weight, Perineal tears, Episiotomy, Postpartum hemorrhage, Caesarean, Instrumental delivery	5	1	1	0	1	1	1
Obaid et al., 2019 [77]	Egypt	Referral level (public or private)	Not available	Any FGM	Any FGM: 100	100	Sexual dysfunction, Anxiety, Depression, PTSD	1	1	1	-1	0	1	-1t
Odoi et al., 1997 [71]	Ghana	Community-based	Both self-reported and clinically reported	Any FGM	Any FGM: 76	119	Dyspareunia	-1	-1	1	-1	0	1	-1
Oduro et al., 2006 [30]	Ghana	Referral level (public or private)	Clinical report based on medical records or physical examination	Any FGM	Any FGM: 1466	3605	Caesarean, Low birthweight, Pregnancy loss	4	1	1	-1	1	1	1
Okonofua et al., 2002 [53]	Nigeria	Referral level (public or private)	Both self-reported and clinically reported	Any FGM	Any FGM: 827	1009	Genital tissue damage, Dyspareunia	3	1	1	-1	0	1	1
Oyefara, 2015 [70]	Nigeria	Community-based	Self-report	Any FGM	Any FGM: 266	84	Dyspareunia	1	1	1	-1	0	1	-1
Pépin et al. 2006 [59]	Guinea-Bissau	Community-based	Self-report	Any FGM	Any FGM: 488	538	STI, including HIV	0	-1	1	-1	1	1	-1
Piroozi et al., 2020 [85]	Islamic Republic of Iran	Referral level (public or private)	Both self-reported and clinically reported	Any FGM	Any FGM: 122	125	Anxiety, Somatoform, Depression	3	1	1	-1	0	1	1
Raheem et al., 2018 [73]	Egypt	Primary care	Both self-reported and clinically reported	Any FGM	Any FGM: 300	36	Sexual dysfunction	0	-1	1	1	-1	1	-1
Rodriguez et al., 2017 [40]	Multi-country study	Primary care	Clinical report based on medical records or physical examination	Any FGM	Any FGM: 1,179	480	Caesarean section	2	1	1	-1	1	1	-1
Saleh et al., 2018 [17]	Egypt	Referral level (public or private)	Clinical report based on medical records or physical examination	Type I and II	Type I and II: 300	150	Prolonged labour 2nd stage, Instrumental delivery, Caesarean, Episiotomy, Obstetric tears/lacerations Postpartum hemorrhage, Low birthweight	4	1	1	-1	1	1	1
Senegal DHS 2005	Senegal	Community-based	Self-reported for others (e.g., children)	Any FGM Type I and II Type III	Any FGM 1445 Type I and II 1245 Type III 139	-	Excessive bleeding, Wound healing problems, Difficulties urinating/urinary retention	-1	1	-1	1	0	-1	-1
Shandall, 1967 [54]	Sudan	Referral level (public or private)	Clinical report based on medical records or physical examination	Type I and Type III	TypeI: 807 Type III: 3013	204	Genital tissue damage, UTI	2	1	1	-1	1	1	-1
Shiferaw et al., 2014 [66]	Ethiopia	Community-based	Self-report	Any FGM	Any FGM: 209	261	Menstrual difficulties	3	1	1	1	0	1	-1
Slanger et al., 2002 [25]	Nigeria	Referral level (public or private)	Both self-reported and clinically reported	Any FGM	Any FGM: 486	621	Caesarean, Episiotomy, Postpartum hemorrhage, Instrumental delivery, Prolonged labour, Obstetric tears/lacerations	5	1	1	1	0	1	1
Suleiman et al., 2021 [32]	United Republic of Tanzania	Referral level (public or private)	Clinical report based on medical records or physical examination	Any FGM	Any FGM: 4675	25,611	Caesarean, Instrumental delivery, Preterm labor, Low birth weight, Stillbirth/Neonatal death	6	1	1	1	1	1	1
Taraldsen et al., 2021 [16]	Norway	Referral level (public or private)	Clinical report based on medical records or physical examination	Type III, Type I and II	Type III: 886, Type I and II: 128	74	Caesarean, Instrumental delivery, Induction of labour, 5-min APGAR < 7, Asphyxia, Stillbirth/Perinatal death, Fetal distress, Prolonged labour	6	1	1	1	1	1	1
Théra et al., 2014	Mali	Referral level (public or private)	Clinical report based on medical records or physical examination	Any FGM	Any FGM: 140	140	Caesarean, obstetric tear, Instrumental delivery, Prolonged labour, Pregnancy loss	2	1	1	-1	1	1	-1
Umbeli et al., 2013 [27]	Sudan	Referral level (public or private)	Clinical report based on medical records or physical examination	Any FGM	Any FGM: 629	1332	Prolonged labour, Instrumental delivery, Obstetric tears/lacerations, Postpartum hemorrhage, Extended maternal hospital stay	2	1	1	-1	1	1	-1
Varol et al., 2016 [39]	Australia	Referral level (public or private)	Clinical report based on medical records or physical examination	Type I and II, Type III	Type I and II: 131 Type III: 65	8852	Obstetric tear/laceration, Caesarean, Episiotomy, Instrumental delivery, Low birthweight, Postpartum hemorrhage, Pregnancy loss	3	1	0	-1	1	1	1
WHO study group, 2006 [37]	Burkina Faso, Ghana, Kenya, Nigeria, Senegal and Sudan	Referral level (public or private)	Clinical report based on medical records or physical examination	Type I and II, Type III	Type I: 6856 Type II: 7771 Type III: 6595	7171	Low birthweight, Caesarean, Episiotomy, Extended maternal hospital stay, Resuscitation of the newborn, Pregnancy loss, Obstetric tears/lacerations, Postpartum hemorrhage	4	1	1	-1	1	1	1
Yassin, Idris and Ali., 2018 [47]	Sudan	Referral level (public or private)	Clinical report based on medical records or physical examination	Any FGM	Any FGM: 230	190	Dyspareunia, Episiotomy, Postpartum hemorrhage	4	1	1	-1	1	1	1
Yemen DHS 1997	Yemen	Community-based	Self-reported for others (e.g., children)	Any FGM	Any FGM 1546	-	Excessive bleeding, Difficulties urinating/urinary retention, Infections, including fever (systemic; urinary; reproductive tract)	1	1	1	1	0	-1	-1
Yount and Abraham, 2007	Kenya	Community-based	Self-report	Any FGM	Any FGM: 1071	2096	STI, including HIV	6	1	1	1	1	1	1

Q1 assesses the sampling: 1 = Sampling was described and representative of population sampled; -1 = Sampling was not described or not representative

Q2 assesses the selection: 1 = Exposed group (FGM +) and non-exposed group (FGM-) were sampled from the same population; 0 = Exposed group and non-exposed group were not sampled from the same population; -1 = Not specified in the study

Q3 assesses the response rate: 1 = Response rate was above 80%; 0 = response rate was 80% or below; -1 = Not specified in the study

Q4 assesses the outcome assessment: 1 = Outcome was assessed by clinical report; -1 = Outcome was assessed using self-report; 0 = Outcome was assessed using other method or assessment method is not specified

Q5 assesses whether exposure and outcome were measured in the same way in both groups: 1 = Exposure and outcome were measured in the same way in both groups; -1 = Exposure and outcome were not measured in the same way in both groups or information on assessment is not reported

Q6 assesses overall biases in the study: 1 = Authors discuss sources of potential biases and/or imprecision in the study, and no major biases are present; -1 = Authors do not discuss potential biases and limitations, or major biases in the study are noted

^a^Total quality scores are based on assessment of six criteria of methodological quality using the scoring described above

### Event

FGM type was classified as the authors reported it and as it relates to the WHO typology. When studies reported the type(s) as Type I, Type II, Type III and Type IV, these classifications were used.

### Comparison

Included studies were comparative, i.e., reporting health outcomes for women with FGM as compared to women without FGM, with the exception of the immediate health outcomes where DHS data on health events amongst women and girls who reported having undergone FGM were analyzed.

### Outcomes

Included studies reported on at least one FGM-related health complication as reported by the study authors. All health complications for which girls or women could seek care were considered for inclusion, if the study met the inclusion criteria described above and in the review protocol [10]. Available data were extracted for all outcomes by FGM type as reported by the authors. The list of outcomes includes those included in the previous review by Berg et al. as well as several additional outcomes that emerged in the literature.

### Languages

No language restrictions were applied. Where studies were not in English, Google Translate was used to assess eligibility. If eligible, translation assistance was sought for data extraction.

### Search strategy

To ensure consistency, the search strategy and terms are aligned with the methods used by Berg et al.. The following databases were searched for the period 2009–2022^2^: African Index Medicus, Anthropology Plus, CINAHL, Cochrane Library, Cochrane Database of Systematic Reviews (CDSR), Cochrane Central Register of Controlled Trials (CENTRAL), EMBASE (Ovid EMBASE), MEDLINE, PILOTS, POPLINE, Social Services Abstracts, Sociological Abstracts, Index Medicus for Eastern Mediterranean Region, Scopus as well as DHS (www.dhs.org). We also searched reference lists of relevant reviews and contacted authors when needed. These strategies identified published and grey literature for screening. The search strategy is described in Supplementary file 1.

### Screening, data selection and collection process

**Fig. 1:**
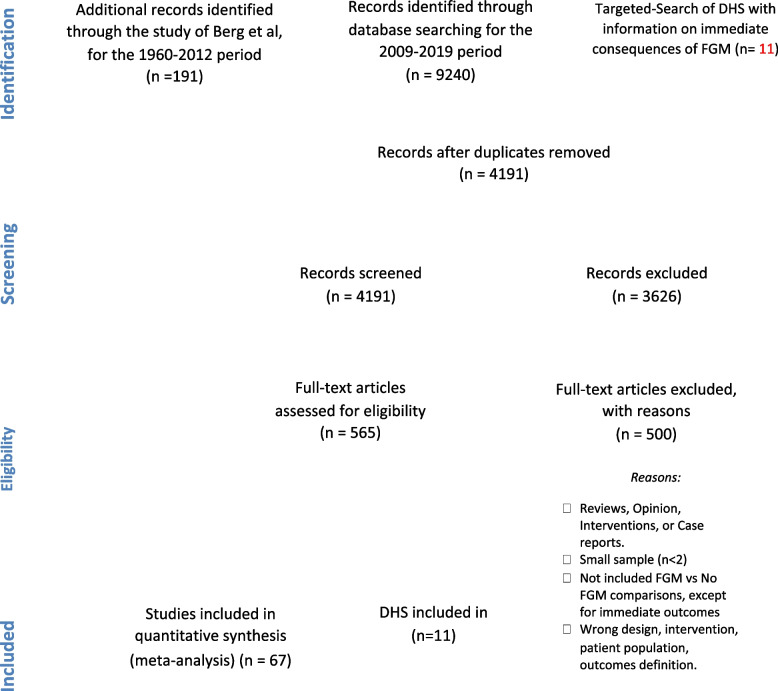
PRISMA flow diagram

Two reviewers independently screened all citations (title and abstract) identified through the searches to assess for potential eligibility. In the case of disagreement or where the information was not sufficient for decision on inclusion/exclusion, the article was included for full text review. Full texts of potentially eligible studies or sources were retrieved and independently assessed for inclusion by two reviewers. Any discrepancies were resolved by discussion and consensus by the two reviewers or through consultation with a third reviewer. Where citations were excluded, the reason for exclusion was documented. All data inclusion and exclusion were reported according to the PRIMSA checklist (Fig. 1).

### Assessing methodological quality

In this review we assessed the quality of included studies using a six-point checklist, adapted from items within the Cochrane’s Effective Practice and Organization of Care (EPOC) risk of bias reporting tool [11]. Checklist items include assessment and scoring (score of -1 to 1) of the following criteria: whether the sampling method was described and representative, whether the exposed and unexposed groups were drawn from the same population, whether the response rate was described and above 80%, predominant method of outcome assessment (self-assessment, clinical assessment or not specified), whether the exposure (FGM) and outcome were measured the same way in both groups, and whether the authors discuss potential sources of bias (or other limitations) and whether significant biases are present. By summing the scores on these six criteria, each study was assigned a total quality rating score. Studies with scores of 5 or 6 were of higher quality (i.e., all or almost all the criteria from the checklist were met), while studies with a quality score of 3 or 4 had some limitations. Studies with scores of 2 or less were considered low quality, given the serious methodological limitations (i.e., few or no criteria were met and/or the study did not adequately report the criteria). Quality scores are summarized in Table 1.

### Data extraction

Data were extracted from each paper on the following variables: health field (i.e., the category of health complication); outcome (i.e., health outcome as defined by the study authors); author(s) and publication date; country; country income level; study design; sampling location: (i.e., facility-based, community based); outcome time (i.e., whether the outcome reflected a one-time event, or a repeat event as defined by the authors); risk period (i.e., whether the study captured events in a defined period or at any point in the life course); whether or not the study involved a pediatric population; FGM type as classified by the study author; FGM assessment method (i.e., self-report, clinical report, or both); outcome assessment method (i.e., self-report, clinical report, or both); whether the estimates provided in the analysis were adjusted or not; comparison statistic (i.e., type of statistical assessment reported by authors (e.g., hazard ratio, crude odds ratio (OR)/relative risk (RR)/coefficient, adjusted OR/RR/coefficient, mean comparison, proportion/percentage comparison)); sample size in exposed and unexposed groups and numbers with outcome in each group. Data from the immediate health complications were the exception and did not include a comparison group since these data were based on questions asked only of mothers who had a daughter who had undergone FGM.

### Statistical analysis

Studies were grouped by health field and separate analyses were conducted for each outcome. Pooled analyses were conducted for outcomes with data from two or more studies. For health outcomes with one study, results are reported directly. Data were analysed using Stata version 17 (StataCorp LLC, College Station, Texas, USA). Odds ratios and 95% confidence intervals were calculated by pooling results from included studies. Adjusted estimates were pooled when available, otherwise, unadjusted estimates were calculated using crude data from individual studies. It was anticipated there would be diversity in the included studies, in terms of geographical settings, outcomes measured and research methodology. Pooled estimates were calculated using random effects models. This method captures both within study variance and between study variation. Heterogeneity was assessed using chi-squared test for heterogeneity. I-squared statistics were reported, where roughly < 70% indicates low heterogeneity, 70%-90% indicates medium heterogeneity, and > 90% indicates high heterogeneity. Pooled estimates were calculated for risk of immediate health complications without comparison groups since these data were only available for girls who had undergone FGM as reported in the DHS.

## Results

A total of 4,191 unique citations were identified and screened. Of these, a total of 78 studies were included (Fig. 1). Details of all included studies are shown in Table 1. Of the 78 studies, 67 were comparative studies (e.g., studies that compared women with FGM with women with no FGM), including 38 cross-sectional comparative studies, 21 case–control studies and eight cohort studies. For the immediate health outcomes 11 cross-sectional population-based studies without comparison groups were analyzed. Girls and women in 68 of the included studies originated from FGM high prevalence countries while 10 studies reported data on girls and women from countries that are home to diaspora communities affected by FGM (i.e., Australia, France, Sweden, Norway, United Kingdom of Great Britain and Northern Ireland, and United States of America). In one study, the origin of the participants was not clearly identifiable. The methodological quality of the included studies was variable, with 12 (15%) studies having a high-quality score, 31 (40%) having a medium score, and 35 (45%) having a low-quality score (Table 1).

### Obstetric and neonatal complications

Thirty six studies (*n* = 124,253) reported on FGM-related obstetric and neonatal complications including prolonged/obstructed labour, obstetric tears/lacerations, caesarean birth, post-partum haemorrhage (PPH), episiotomy, preterm delivery, instrumental delivery, extended maternal hospital stay, neonatal asphyxia, low birthweight (LBW) and stillbirth/neonatal death. All results are summarized in Table 2.

**Table 2:** Results of the meta-analyses of FGM-related health complications

Health complication	FGM type as reported	No. of studies/ estimates	OR (95% CI)	Heterogeneity score I^2^
**OBSTETRIC**				
**Prolonged/obstructed labour**				
	Any FGM	14	2,61 (1,61—4,23)	91,5%
	Type I/II	14	1,96 (1,46—2,63)	46.40%
	Type II/III	-	-	-
**Obstetric tears/lacerations**				
	Any FGM	27	2,45 (1,83—3,28)	73,5%
	Type I/II	19	2,07 (1,59 – 2,68)	72.10%
	Type II/III	5	2,17 (1,08 -4,36)	64.70%
**Caesarean birth**				
	Any FGM	27	1,36 (1,09 – 1,69)	94.10%
	Type I/II	27	0,96 (0,85—1,09)	57,60%
	Type II/III	11	0,79 (0,62—1,00)	69.30%
**Postpartum hemorrhage**				
	Any FGM	18	2,24 (1,72 – 2,92)	77,8%
	Type I/II	12	1,28 (1,14—1,44)	50.60%
	Type II/III	6	1,15 (0,91—1,45)	65.10%
**Episiotomy**				
	Any FGM	17	1,70 (1,21—2,38)	92,5%
	Type I/II	13	2,02 (1,22 – 3,32)	88.80%
	Type II/III	3	6,58 (4,19 -10,33)	94.00%
**Preterm birth**				
	Any FGM	3	1,60 (0,80 – 3,23)	96,2%
	Type I/II	4	1,00 (0,65—1,52)	0.00%
	Type II/III	-	-	-
**Instrumental delivery**				
	Any FGM	18	1,23 (0,96—1,57)	64.50%
	Type I/II	4	1,00 (0,65 – 1,52)	0.0%
	Type II/III	3	1,10 (0,30 – 3,99)	55.20%
**Induction of Labor**				
	Type I/II	2	0,96 (0,60 – 1,54)	0.0%
**Fetal distress**				
	Any FGM	3	1,94 (1,56 – 2,42)	0.0%
	Type I/II	6	2,32 (1,01 – 5,32)	64.60%
	Type II/III	-	-	-
**Extended maternal hospital stay**				
	Any FGM	4	2,80 (1,52 – 5,16)	98.2%
	Type I/II	6	1,29 (1,08—1,53)	86.3%
	Type II/III	3	0.92 (0,74 – 1,13)	75,70%
**Neonatal asphyxia**				
	Any FGM	9	1,79 (1,36 – 2,37)	54,5%
	Type I/II	12	1,32 (1,14—1,53)	42.00%
	Type II/III	6	0,96 (0,67—1,39)	69.20%
**Low birthweight**				
	Any FGM	10	1,14 (1,00—1,29)	66.30%
	Type I/II	11	1,15 (1,05—1,26)	48.3%
	Type II/III	6	0,82 (0,75—0,89)	0.00%
**Stillbirth/Neonatal death**				
	Any FGM	19	1,58 (1,34 – 1,86)	55,00%
	Type I/II	16	1,51 (1,41 – 1,63)	0.0%
	Type II/III	6	0,73 (0,57 – 0.94)	52.00%
**GYNECOLOGICAL**				
**Infertility**				
	Any FGM	14	0,98 (0,76—1,27)	97.40%
	Type I/II	4	0,79 (0,70—0,90)	0.00%
	Type II/III	3	1,22 (0,94—1,58)	78.80%
**Genital tissue damage**				
	Any FGM	3	2,23 (1,08 – 4,61)	22,5%
	Type I/II	8	9,02 (2,76—29,48)	58.20%
	Type II/III	-	-	-
**Sexually transmitted infections, including HIV**				
	Any FGM	17	0,74 (0,58—0,94)	65.80%
	Type I/II	-	-	-
	Type II/III	-	-	-
**Reproductive tract infections**				
	Any FGM	9	1,88 (1,11 – 3,18)	87.6%
	Type I/II	-	-	-
	Type II/III	8	1,80 (1,05—3,11)	89.00%
**Menstrual difficulties**				
	Any FGM	11	1,73 (1,11—2,69)	87.70%
	Type I/II	3	3,41 (2,41 – 4,84)	30,6%
	Type II/III	-	-	-
**Fistulae**				
	Any FGM	13	1,01 (0,80 – 1,28)	69.10%
	Type I/II	-	-	-
	Type II/III	-	-	-
**UROLOGICAL**				
**Urinary tract infections**				
	Any FGM	6	3,59 (2,22—5,81)	84,5%
	Type I/II	5	3,47 (2,02 – 5,96)	68,6%
	Type II/III	3	1,00 (0,64 – 1,56)	0.00%
**Urinary incontinence**				
	Any FGM	4	1,02 (0,53—1,96)	37.00%
	Type I/II	-	-	-
	Type II/III	-	-	-
**Difficulty urinating**				
	Any FGM	4	1,79 (1,19—2,67)	68,8%
	Type I/II	-	-	-
	Type II/III	-	-	-
**SEXUAL**				
**Dyspareunia**				
	Any FGM	11	3,88 (2,32 – 6,51)	90.90%
	Type I/II	-	-	-
	Type II/III	-	-	-
**Sexual dysfunction**	Any FGM	5	3,20 (1,75 – 5,84)	75.20%
	Type I/II	-	-	-
	Type II/III	-	-	-
**MENTAL**				
**Depression or Anxiety**				
	Any FGM	8	2,90 (1,53 – 5,51)	78.80%
	Type I/II	-	-	-
	Type II/III	-	-	-
**Post-traumatic stress disorder**				
	Any FGM	3	4,42 (0,77 – 25,37)	42.80%
	Type I/II	1	0,10 (0,00 – 2,47)	-
	Type II/III	1	3,83 (0,48—30,93)	-
**Somatoform**				
	Any FGM	3	1,72 (1,24 – 2,37)	0.0%
	Type I/II	-	-	-
	Type II/III	1	5,56 (0,31—98,75)	
**IMMEDIATE**				
**Excessive bleeding**				
	Any FGM	11	0,20 (0,15—0,26)	99.20%
	Type I/II	10	0,22 (0,16–0,28)	99.20%
	Type II/III	6	0,31 (0,20—0,41)	95.20%
	Type IV	7	0,22 (0,05—0,39)	98.50%
**Wound healing problems**				
	Any FGM	7	0,11 (0,07—0,15)	99.10%
	Type I/II	7	0,08 (0,05—0,12)	98.40%
	Type II/III	5	0.14 (0.02 – 0.25)	97.70%
	Type IV	5	0,20 (0,04—0,09)	99.00%
**Difficulties urinating/Urine retention**				
	Any FGM	8	0,23 (0,14—0,32)	99.70%
	Type I/II	7	0,25 (0,17—0,32)	99.30%
	Type II/III	4	0,35 (0,28—0,42)	77.60%
	Type IV	5	0,20 (0,04—0,36)	98.30%
**Infections including fever (systemic, urinary, reproductive tract)**				
	Any FGM	5	0,10 (0,04—0,17)	99.30%
	Type I/II	5	0,14 (0,08—0,21)	98.10%
	Type II/III	2	0,06 (0,02 -0,09)	0.00%
	Type IV	3	0,12 (0,00—0,23)	95.20%
**Swelling in genital area**				
	Any FGM	2	0,09 (0,04—0,14)	84.40%
	Type I/II	1	0,06 (0,05—0,07)	-
	Type II/III	1	0,12 (0,05—0,20)	-
	Type IV			

Compared to women without FGM, women who had any form of FGM [OR = 2,61 (95% CI = 1,62 – 4,23), *n* = 14,282] and those with types I/II [OR = 1,96 (95% CI = 1,46—2,63), *n* = 4,884] [12–27] were significantly more likely to experience prolonged/obstructed labour. Women with any form of FGM [OR = 1,58 (95% CI = 1,34 – 1,86), *n* = 90,586] [22, 23, 26, 28–36] and those with types I/II [OR = 1,51 (95% CI = 1,41—1,63), *n* = 41,774] [12–14, 16, 37–39] were also significantly more likely to experience stillbirth/neonatal death. We did not find a significant association between this outcome and women with types II/III [OR = 0,73 (95% CI = 0,57—0,94), *n* = 39,172] [16, 37–39]. There was a trend towards a significantly increased likelihood of undergoing a caesarean birth [OR = 1,36 (95% CI = 1,09 – 1,69), *n* = 77,088] [19, 21, 22, 24–26, 28, 30–33, 35, 36, 40–44] among women with any form of FGM while no association was found for women with FGM types I/II [OR = 0,96 (95% CI = 0,85—1,09)] [12–17, 37–39] or those with types II/III [OR = 0,79 (95% CI = 0,62—1,00)] [15, 16, 37–39].

Women with any type of FGM [OR = 2,45 (95% CI = 1,83—3,28), *n* = 36,300] [19, 21–23, 25–29, 31, 33, 35, 36, 41–45], those with FGM types I/II [OR = 2,07 (95% CI = 1,59 – 2,68), *n* = 41,467] [12, 13, 15, 17, 37–39] and those with types II/III [OR = 2,17 (95% CI = 1,08 – 4,36), *n* = 38,084] [37–39] were significantly more likely to suffer obstetric perineal tears/lacerations compared to those without. Further, women with any type of FGM [OR = 1,70 (95% CI = 1,21 – 2,38), *n* = 58,395] [21, 23, 25, 27, 29, 31–33, 35, 36, 41, 43, 44, 46–48]; those with types I/II [OR = 2,02 (95% CI = 1,22 – 3,32), *n* = 12,844] [12–15, 17, 39] and those with types II/III [OR = 6,58 (95% = 4,19 – 10,33), *n* = 37,441] [37, 39] were significantly more likely to require an episiotomy during child birth.

Women with any type of FGM [OR = 2,24 (95% CI = 1,72 – 2,92), *n* = 56,122] (18, 19, 22–25, 27–29, 31, 32, 36, 41, 45, 47, and those with types I/II [OR = 1,28 (95% CI = 1,14 – 1,44), *n* = 39,278] [15, 17, 37, 38] were significantly more likely to experience PPH compared to those without. Although not statistically significant, women with FGM types II/III were also significantly more likely to suffer PPH [OR = 1,15 (95% CI = 0,91—1,45), *n* = 38,828] [15, 37–39]. Similarly, women with any type of FGM [OR = 2,80 (95% CI = 1,52 – 5,16), *n* = 37,895] [24, 27, 28, 32, 50] and those with types I/II [OR = 1,28 (95% CI = 1,08—1,53), *n* = 28,393] [37] were significantly more likely to have an extended hospital stay after childbirth compared to those without FGM. Additionally, women with any type of FGM [OR = 1,79 (95% CI = 1,36 – 2,37), *n* = 47,265] [23, 24, 27, 28, 31, 32, 44] and those with types I/II [OR = 1,32 (95% CI = 1,14—1,53), *n* = 40,035] [14, 16, 17, 37–39] were significantly more likely to give birth to a newborn with asphyxia. We did not find a statistically significant association for this outcome among women with FGM types II/III [OR = 0,96 (95% CI = 0,67—1,39), *n* = 39,172] [16, 37–39].

There was a trend towards a significant association between women with any type of FGM [OR = 1,14 (95% CI = 1,00—1,30), *n* = 47,395] [20, 30, 32, 36, 49] and giving birth to a LBW baby, while women with types I/II [OR = 1,15 (95% CI = 1,05—1,26), *n* = 38,828] [15, 37–39] had a significantly increased likelihood of giving birth to a LBW baby. Conversely, women with types II/III [(OR = 0,82 (95% CI = 0,75 – 0,89), *n* = 38,828] had a significantly reduced likelihood of giving birth to a LBW baby [15, 37–39]. We did not find a significant association between women with any type of FGM [OR = 1,23 (95% CI = 0,96 – 1,57), *n* = 65,368] [19–21, 23, 25–27, 32, 35, 36, 41–44]; those with types I/II [OR = 1,35 (95% CI = 0,77 – 2,36), *n* = 11,229] [16, 17, 38, 39] or those with types II/III [OR = 1,10 (95% CI = 0,30 – 3,99), *n* = 10,779] [16, 38, 39] and undergoing an instrumental delivery. No association was found between induction of labor and FGM types I/II [(OR = 0,96 (95% CI = 0,60 – 1,54), *n* = 10,068] [16, 38]. Similarly, we did not find a significant association between women with any type of FGM [OR = 1.60 (95% CI = 0,80 – 3,23), *n* = 35,264] [24, 32, 48] and those with types I/II [OR = 1,00 (95% CI = 0,65 – 1,52), *n* = 1,387] [15, 38] and experiencing a preterm birth. Conversely, women with any type of FGM [OR = 1.94 (95% CI = 1,56 – 2,42), *n* = 6,500] [24, 28, 43] and those with types I/II [OR = 2,32 (95% CI = 1,01 – 5,32), *n* = 2,301] [13, 16, 38] had a significantly increased likelihood of experiencing fetal distress.

### Gynecological complications

Twenty-seven studies (*n* = 316,191) provided data on the gynecological complications of FGM including infertility; genital tissue damage (keloids, clitoral neuroma, and vulvar cysts); sexually transmitted infections (STIs) including chlamydia trachomatis, syphilis, trichomoniasis and HIV; reproductive tract infections; menstrual difficulties and fistulae.

While there was no significant association between infertility and women with any type of FGM [OR = 0,98 (95% CI = 0,76 – 1,27), *n* = 113,340] [18, 28, 33, 34, 50] or those with types II/III [OR = 1,22 (95% CI = 0,94—1,58), *n* = 43,039] [51, 52], we found a significantly reduced likelihood of infertility [OR = 0,79 (95% CI = 0,70—0,90), *n* = 43,039] among women with FGM types I/II [51, 52]. Women with any type of FGM [OR = 2,23 (95% CI = 1,08 – 4,61), *n* = 3,841] [28, 34, 53] and those with types I/II [OR = 9,02 (95% CI = 2,76 – 29,29), *n* = 4,594] [13, 54] had a significantly increased likelihood of genital tissue damage. Conversely, women with any type of FGM [OR = 0,74 (95% CI = 0,58—0,94), *n* = 17,873] [19, 34, 55–61] had a significantly reduced likelihood of presenting with an STI including HIV, while those with any type of FGM [OR = 1,88 (95% CI = 1,11—3,18), *n* = 5,009] [18, 19, 28, 34, 57, 62] had a significantly increased likelihood of presenting with a reproductive tract infection. Women with any type of FGM [OR = 1,73 (95% CI = 1,11—2,69), *n* = 8,677] [28, 33, 34, 45, 62–66] and those with types I/II [OR = 3,41 (95% CI = 2,41 – 4,84), *n* = 570] [13] had a significantly increased likelihood of suffering from menstrual difficulties compared to those without. No association was found when comparing women with any type of FGM to women with no FGM in terms of risk of fistulae [OR = 1,01 (95% CI = 0,80 – 1,28), *n* = 124,179] [28, 34, 67, 68].

### Urological complication

Nineteen studies provided data on urological complications of FGM including urinary tract infections (UTIs), urinary incontinence and urinary retention.

There was a significantly increased likelihood of UTIs among women with any type of FGM [OR = 3,59 (95% CI = 2,21—5,81), *n* = 10,589] [18, 19, 24, 28, 35] and those with types I/II [OR = 3,47 (95% CI = 2,02 – 5,96), *n* = 4,594] [13, 54]. However, we did not find an association [OR = 1,00 (95% CI = 0,64 – 1,56), *n* = 4,024] [54] between women with FGM types II/III and UTIs. Similarly, we did not find an association between women with any type of FGM [OR = 1,02 (95% CI = 0,53—1,96), *n* = 4,129] [29, 33, 35] and urinary incontinence. Conversely, we found a significantly increased likelihood of urinary difficulties, including urinary retention and dysuria, among women with any type of FGM [OR = 1,79 (95% CI = 1,19—2,67), *n* = 6,205] [22, 28, 63, 64].

### Sexual health complications

Fourteen studies examined the association between FGM status and sexual health complications including painful sexual intercourse (dyspareunia) and sexual dysfunction. Women with any type of FGM had a significantly increased likelihood of dyspareunia [OR = 3,88 (95% CI = 2,32 – 6,50), *n* = 8,534] [18, 28, 33–35, 47, 53, 65, 69–71] and sexual dysfunction [OR = 3,20 (95% CI = 1,75 – 5,84), *n* = 2,118] [28, 33, 46, 72, 73]. In an analysis of mean scores on the Female Sexual Function Index (FSFI) by FGM status, women with any type of FGM had lower FSFI scores as compared to women without FGM [MD = -0,06 (95% CI = -0,14, 0,02), *n* = 4,541] [45, 74–79]. Likewise, women with types I/II [MD = -0,16 (95% CI = -0,27, 0,04), *n* = 4,541] had significantly lower mean FSFI scores compared to women without FGM [80, 81]. The mean difference in FSFI scores for women with type III FGM compared to women without FGM was 7,74 (95% CI = 5,13, 10,35) [82].

### Mental health complications

Seven studies (*N* = 2,119) examined the association between FGM status and mental health complications including post-traumatic stress disorder (PTSD), anxiety, depression, and somatoform disorders. Women with any type of FGM had an increased likelihood of PTSD [OR = 1,83 (95% CI = 1,27 – 2,64), *n* = 905] [28, 83–87]; depression and/or anxiety disorders [OR = 2,90 (95% CI = 1,53 – 5,51), *n* = 1,745] [28, 83, 85–88] as well as somatoform disorders [OR = 1,72 (95% CI = 1,24—2,37), *n* = 646] [85–87] compared to women without FGM.

### Immediate health complications

Data on the immediate health complications following FGM were extracted from 11 DHS reports (*n* = 49,990) using responses on mothers’ assessment of the health complications of their daughters following FGM [89–99]. No comparison group is available for these immediate outcomes since these questions were only asked of mothers who responded affirmatively when asked if their daughter(s) had undergone FGM. These immediate health complications included difficulties with urinating/urine retention, excessive bleeding (hemorrhage), infections including fever, swelling in the genital area and wound healing problems/infection of the wound.

From the data, an estimated 23,3% of girls who underwent some form of FGM experienced difficulties urinating; 20,4% presented with excessive bleeding; 10,9% had wound healing problems; 10,3% had an infection and 9% had swelling in the genital area. Among girls whose mothers reported that they underwent FGM type I/II, 25% presented with difficulties with urination; 22,5% had excessive bleeding of the wound; 14,7% had an infection; 8,9% had wound healing problems; and 6,5% had swelling of the genital area. Of girls whose mothers reported they were stitched closed (corresponding to FGM type III), 35,7% encountered difficulties urinating; 31,1% had excessive bleeding; 13,8% had wound healing problems; 12,9% had severe genital tissue swelling; and 6,2% had an infection. Girls whose mothers reported the equivalent of type IV FGM also experienced complications: 22,3% suffered from excessive bleeding; 20,4% had wound healing problems; 20,4% had difficulties urinating; and 12% had an infection after undergoing the procedure.

## Discussion

The results of this systematic review and meta-analyses provide evidence that FGM is associated with several health complications for girls and women over the course of their lifespan. As has been previously reported, women who have undergone FGM have significantly higher likelihood of multiple obstetric, neonatal, gynecological, urological, sexual and mental health complications compared to those who have not. Between 9 and 24% of girls whose mothers reported that they had undergone FGM also experienced immediate health complications. These findings are consistent with previous reviews on the health complications of FGM [4, 5, 8], providing pooled estimates with additional comparisons for women with different types of FGM. To assess sexual satisfaction, we provide data in the form of pooled mean FSFI scores which indicate that women who have undergone FGM have significantly lower mean FSFI scores compared to those who have not. Importantly, this additional evidence builds on the inconclusive findings from a previous review [4] regarding the effect of FGM on sexual health complications.

These findings are relevant for health policy makers, health workers, advocates and women and girls affected by or at risk of FGM. By identifying the health complications that are significantly associated with FGM, these findings can inform the development and update of clinical guidelines, clinical management tools and health worker training content, while also serving as the basis for building relevant capacities of health workers in assessing for and managing these health complications. Indeed, previous reviews on health outcomes of FGM informed the development of WHO’s guidelines and clinical handbook, which aim to ensure that women and girls affected by FGM receive the highest quality care possible. The adaptation and implementation of these clinical management tools in high prevalence countries is a key component of WHO’s health system strengthening approach to FGM.

The evidence base on health complications ensures the integration of appropriate health interventions into essential sexual and reproductive health service packages at a global and national level, such as the universal health coverage (UHC) Compendium [100], a database that assists countries in developing UHC packages relevant to context and need, itemizing the key interventions needed to prevent and address health complications throughout the life course. In addition, the findings of this research informed the study on the global and national costs of treating FGM-related complications throughout the life course in 27 high prevalence countries, which found that treating FGM-related complications costs health systems 1.4 billion USD per year [101]. Health conditions identified in this meta-analysis as being significantly associated with FGM were included in the economic models and were the basis for quantifying the relative health risk of women who have undergone FGM as compared to those who have not, by FGM type.

While these findings provide critical information to inform a public health response to FGM, limitations in the available evidence and the analysis must be noted. First, the analyses categorized results by FGM type as reported in the included studies. Some authors did not consistently categorize FGM, using a broad definition of any FGM or did not specify how they categorized it, while other studies explored health complications among women with only specific FGM types. Analytically, we conducted separate analyses for health outcomes based on the categorizations used in the studies recognizing that some studies only captured data on one type of FGM, and some used a joint categorization. We were limited by these categorizations but ensured that we did not analyze FGM Type II in multiple analyses for the same outcome when this group was unclearly defined. The default option when faced with lack of specificity was to include it in the Type II/III category. Table 1 details the types of FGM captured in the included studies and how they were analyzed in the meta-analyses.

Second, the definitions of health complications were categorized by the authors, and there was considerable variability in the outcomes measured and the definitions used for these outcomes. Overly general and overly specific categorizations present analytical challenges in meta-analyses such as this, making it difficult to combine studies that assess for a specific and uncommon outcome with studies that include a broader categorization that would be more likely to occur. Table 1 shows the outcomes reported in the studies, and the forest plots (Supplementary file 2) show which studies contributed data to each analysis. The lack of consistency in definitions and variability in study designs explain the high heterogeneity reported for the meta-analyses. It is also important to note that roughly half the included studies were considered low quality and less than 15% were considered high quality, raising questions about the generalizability of the findings.

Third, this systematic review and meta-analyses included observational data, that do not adequately account for potential confounding. The findings on several obstetric and neonatal consequences showed unstable and conflicting findings, such as in the case of caesarean birth, which varied by FGM type. While FGM might increase risk of intrapartum complications requiring a caesarean birth, health workers attending to pregnant women or girls might also be more inclined to use such an approach to delivery to avoid addressing women's FGM status. Additionally, we cannot account for the the influence of factors related to socioeconomic status, gender dynamics, health seeking behaving and other confounding factors that might increase the risk of health complications and also be associated with increased FGM risk.

The methodological and analytical choices used in this study ensured some consistency in inclusion across studies but excluded studies with relevant findings about the reported or additional health complications. For example, no studies reported on FGM-related mortality, which is admittedly a rare event, and one that would be difficult to capture through comparative study designs. However, the severity of the outcome, the fact that it affects girls early in life and its devastating impact on families, necessitates its consideration in any review of evidence on the health impacts of FGM. Its absence in this analysis also serves as a reminder of the many unquantifiable ways that FGM impacts the lives of girls, women and families. While this review is focused on the health burden based on studies including reference groups, there is a large body of literature detailing the psycho-social impacts of FGM using qualitative methods or case reports, showing that FGM can have lasting effects on women’s sense of identity, self-esteem, well-being and can even affect their participation in society [102]. A recent review [103] and call to action [104] highlighted the importance of not only understanding the health complications of FGM but applying that knowledge to improve awareness and knowledge of women at-risk, affected communities, health workers, and policy makers to ensure concerted actions to end the practice and support those affected.

Findings regarding the immediate health outcomes were informed by DHS data from seven countries. While meta-analyses were conducted for the health complications included in those studies (e.g. excessive bleeding, wound healing problems, infections, difficulties urinating, urinary retention, infections, including fever, and swelling of the genitals), it was not possible to calculate ORs and to make comparisons to unexposed groups because data on these outcomes were only available for girls whose mothers reported that their daughters had undergone FGM and there was no comparison group. The outcomes measured would not be expected in unexposed groups, but nonetheless this represents a limitation of the findings on the immediate health outcomes. Despite there not being a comparison group, these data provide important information about the health complications of FGM.

In general, this review provides clear evidence of the harm caused by FGM, but it also highlights the methodological limitations of the existing research in terms of lack of comparability in definitions of FGM and health outcomes. Many studies were excluded because of the lack of comparison groups, and most included studies fail to control for confounding factors. While there are gaps in evidence revealed in this analysis, we do not necessarily promote generation of more evidence on health risk through clinical studies. Rather, what is needed is more evidence on what the health sector and other sectors can do to prevent the practice and respond to existing complications. More observational studies of association will not result in significant improvement in care – the available evidence demonstrates sufficiently the many health improvements that could be achieved if FGM were prevented in the first place.

## Conclusion

This study complements previous reviews summarizing the health complications associated with FGM over the life course. It is hoped that this comprehensive summary of the evidence provides a definitive answer to the question of how FGM can harm women’s physical, psychological and mental health and creates a sense of urgency in the need for action to prevent the practice. FGM is entirely preventable, and the health complications attributable to FGM are likewise preventable. These results can also guide clinical management priorities, training materials and protocols for health workers caring for women and girls who have undergone the practice to ensure they have adequate information and skills to provide quality care in managing complications and preventing the practice.

## Supplementary Information

Supplementary Material 1.

Supplementary Material 2.

## Data Availability

The dataset will be retained in the WHO/HRP electronic archival system. Any use of the dataset for secondary research purposes will be governed by the WHO data use regulation. Requests for data may be sent to pallittoc@who.int.

## Footnotes

1 http://www.cochrane.org/glossary

2 Previous reviews included the Ovid British Nursing Index and Archive; however, this was no longer updated from January 2012. Similarly, the Cochrane EPOC register is no longer maintained and thus was not searched.

## References

[1] World Health Organization. Eliminating female genital mutilation: an interagency statement-OHCHR, UNAIDS, UNDP, UNECA, UNESCO, UNFPA, UNHCR, UNICEF, UNIFEM. WHO: World Health Organization; 2008.

[2] World Health Organization. 2018. Care of girls and women living with female genital mutilation: a clinical handbook. WHO: World Health Organization. https://iris.who.int/handle/10665/272429.

[3] UNICEF. Female Mutilation/Cutting: a global concern:2024 Update. https://data.unicef.org/resources/female-genital-mutilation-a-global-concern-2024/. Accessed 20 Dec 2024.

[4] Berg RC, Denison E, Fretheim A. NIPH Systematic Reviews. Psychological, Social and Sexual Consequences of Female Genital Mutilation/Cutting (FGM/C): A Systematic Review of Quantitative Studies. 2010. 29320049

[5] Berg RC, Underland V. The obstetric consequences of female genital mutilation/cutting: a systematic review and meta-analysis. Obstet Gynecol Int. 2013;2013:496564. 23878544 10.1155/2013/496564 PMC3710629

[6] Berg RC, Vigdis U, Jan O-J, Atle F, Gunn EV. Effects of female genital cutting on physical health outcomes: a systematic review and meta-analysis. BMJ Open. 2014;4(11):e006316. 25416059 10.1136/bmjopen-2014-006316 PMC4244458

[7] Obermeyer CM. The consequences of female circumcision for health and sexuality: an update on the evidence. Cult Health Sex. 2005;7(5):443–61. 16864215 10.1080/14789940500181495

[8] Sarayloo K, Latifnejad Roudsari R, Elhadi A. Health Consequences of the Female Genital Mutilation: A Systematic Review. Galen Med J. 2019;1(8):e1336. 10.22086/gmj.v8i0.1336. 10.22086/gmj.v8i0.1336 PMC8343977 34466496

[9] Moher D, Liberati A, Tetzlaff J, Altman DG; PRISMA Group. Preferred reporting items for systematic reviews and meta-analyses: the PRISMA statement. PLoS Med. 2009;6(7):e1000097. 10.1371/journal.pmed.1000097 PMC2707599 19621072

[10] Gianna R, Vogel JP, Vallejo FR, Pallitto C. The health consequences of female genital mutilation: protocol for a systematic review and meta-analysis. PROSPERO 2020 CRD42020164693 Available from: https://www.crd.york.ac.uk/prospero/display_record.php?ID=CRD42020164693

[11] Cochrane Effective Practice and Organisation of Care (EPOC). EPOC Resources for review authors, 2017. Available at: epoc.cochrane.org/resources/epoc-resources-review-authors.

[12] Larsen U, Okonofua FE. Female circumcision and obstetric complications. Int J Gynaecol Obstet. 2002;77(3):255–65. 12065141 10.1016/s0020-7292(02)00028-0

[13] Kaplan A, Forbes M, Bonhoure I, Utzet M, Martín M, Manneh M, et al. Female genital mutilation/cutting in The Gambia: long-term health consequences and complications during delivery and for the newborn. Int J Womens Health. 2013;5:323–31. 23843705 10.2147/IJWH.S42064 PMC3702244

[14] Frega A, Puzio G, Maniglio P, Catalano A, Milazzo GN, Lombardi D, et al. Obstetric and neonatal outcomes of women with FGM I and II in San Camillo Hospital. Burkina Faso Arch Gynecol Obstet. 2013;288(3):513–9. 23471548 10.1007/s00404-013-2779-y

[15] Anikwe CC, Ejikeme BN, Obiechina NJ, Okorochukwu BC, Obuna JA, Onu FA, et al. Female genital mutilation and obstetric outcome: A cross-sectional comparative study in a tertiary hospital in Abakaliki South East Nigeria. Eur J Obstet Gynecol Reprod Biol X. 2019;1:100005. 31396592 10.1016/j.eurox.2019.100005 PMC6683977

[16] Taraldsen S, Vangen S, Oian P, Sorbye IK. Female genital mutilation/cutting, timing of deinfibulation, and risk of cesarean section. Acta Obstet Gynecol Scand. 2021;100(4):587–95. 33719034 10.1111/aogs.14111

[17] Saleh WF, Torky HA, Youssef MA, Ragab WS, Ahmed MAS, Eldaly A. Effect of female genital cutting performed by health care professionals on labor complications in Egyptian women: a prospective cohort study. J Perinat Med. 2018;46(4):419–24. 28753544 10.1515/jpm-2016-0429

[18] Kasim K, Shaaban S, El Sadak A, Hassan H. Impacts of female genital mutilation on women’s reproductive health. J Community Med Health Edu. 2012;2(137):2.

[19] De Silva S. Obstetric sequelae of female circumcision. Eur J Obstet Gynecol Reprod Biol. 1989;32(3):233–40. 2792544 10.1016/0028-2243(89)90041-5

[20] Essén B, Sjöberg NO, Gudmundsson S, Ostergren PO, Lindqvist PG. No association between female circumcision and prolonged labour: a case control study of immigrant women giving birth in Sweden. Eur J Obstet Gynecol Reprod Biol. 2005;121(2):182–5. 16054959 10.1016/j.ejogrb.2004.12.010

[21] Gudu W, Abdulahi M. Labor, delivery and postpartum complications in nulliparous women with female genital mutilation admitted to Karamara Hospital. Ethiop Med J. 2017;55(1):11–7. 29148634

[22] Gebremicheal K, Alemseged F, Ewunetu H, Tolossa D, Ma’alin A, Yewondwessen M, et al. Sequela of female genital mutilation on birth outcomes in Jijiga town, Ethiopian Somali region: a prospective cohort study. BMC Pregnancy Childbirth. 2018;18(1):305. 30029634 10.1186/s12884-018-1937-4 PMC6053719

[23] Millogo-Traore F, Kaba ST, Thieba B, Akotionga M, Lankoande J. Maternal and foetal prognostic in excised women delivery. J Gynecol Obstet Biol Reprod (Paris). 2007;36(4):393–8. 17418983 10.1016/j.jgyn.2007.03.002

[24] Chibber R, El-Saleh E, El Harmi J. Female circumcision: obstetrical and psychological sequelae continues unabated in the 21st century. J Matern Fetal Neonatal Med. 2011;24(6):833–6. 21121711 10.3109/14767058.2010.531318

[25] Slanger TE, Snow RC, Okonofua FE. The impact of female genital cutting on first delivery in southwest Nigeria. Stud Fam Plann. 2002;33(2):173–84. 12132637 10.1111/j.1728-4465.2002.00173.x

[26] Théra T, Kouma A, Touré M, Coulibaly A, Sima M, Ongoiba I, et al. Obstetrical complications of genital mutilation in Malian rural environment. J Gynecol Obstet Biol Reprod (Paris). 2015;44(3):276–9. 24461341 10.1016/j.jgyn.2013.12.004

[27] Umbeli TA, Salah I, AK, Rabaa A, Najwa A, Sulman MM. Impact of female genital mutilation on second stage of labour in primigravida at Omdurman Maternity Hospital, Sudan 2010. Sudan JMS. 2013;(1):1–4.

[28] Johnson-Agbakwu CE, Fox KA, Banke-Thomas A, Michlig GJ. Influence of Female Genital Mutilation/Cutting on Health Morbidity, Health Service Utilization and Satisfaction with Care among Somali Women and Teenage Girls in the United States. J Racial Ethn Health Disparities. 2023;10(2):788–96. 35258838 10.1007/s40615-022-01266-x PMC9988763

[29] Hakim LY. Impact of female genital mutilation on maternal and neonatal outcomes during parturition. East Afr Med J. 2001;78(5):255–8. 12002086

[30] Oduro A, Ansah P, Hodgson A, Afful T, Baiden F, Adongo P, et al. Trends in the prevalence of female genital mutilation and its effect on delivery outcomes in the kassena-nankana district of northern ghana. Ghana Med J. 2006;40(3):87–92. 17299573 10.4314/gmj.v40i3.55258 PMC1790849

[31] Ndiaye P, Diongue M, Faye A, Ouedraogo D, Tal DA. Female genital mutilation and complications in childbirth in the province of Gourma (Burkina Faso). Sante Publique. 2010;22(5):563–70. 21360865

[32] Suleiman IR, Maro E, Shayo BC, Alloyce JP, Masenga G, Mahande MJ, et al. Trend in female genital mutilation and its associated adverse birth outcomes: A 10-year retrospective birth registry study in Northern Tanzania. PLoS ONE. 2021;16(1):e0244888. 33406158 10.1371/journal.pone.0244888 PMC7787528

[33] Elnashar A, Abdelhady R. The impact of female genital cutting on health of newly married women. Int J Gynaecol Obstet. 2007;97(3):238–44. 17445819 10.1016/j.ijgo.2007.03.008

[34] Morison L, Scherf C, Ekpo G, Paine K, West B, Coleman R, et al. The long-term reproductive health consequences of female genital cutting in rural Gambia: a community-based survey. Tropical Med Int Health. 2001;6(8):643–53. 10.1046/j.1365-3156.2001.00749.x 11555430

[35] Andro A, Cambois E, Lesclingand M. Long-term consequences of female genital mutilation in a European context: Self perceived health of FGM women compared to non-FGM women. Soc Sci Med. 2014;106:177–84. 24565761 10.1016/j.socscimed.2014.02.003

[36] Nonterah EA, Kanmiki EW, Agorinya IA, Sakeah E, Tamimu M, Kagura J, et al. Prevalence and adverse obstetric outcomes of female genital mutilation among women in rural Northern Ghana. Eur J Pub Health. 2020;30(3):561–7. 31637426 10.1093/eurpub/ckz195

[37] WHO study group on female genital mutilation and obstetric outcome, Banks E, Meirik O, Farley T, Akande O, Bathija H, Ali M. Female genital mutilation and obstetric outcome: WHO collaborative prospective study in six African countries. Lancet. 2006;367(9525):1835–41. 10.1016/S0140-6736(06)68805-3 16753486

[38] Minsart AF, N’Guyen TS, Ali Hadji R, Caillet M. Maternal infibulation and obstetrical outcome in Djibouti. J Matern Fetal Neonatal Med. 2015;28(14):1741–6. 25234101 10.3109/14767058.2014.967676

[39] Varol N, Dawson A, Turkmani S, Hall JJ, Nanayakkara S, Jenkins G, et al. Obstetric outcomes for women with female genital mutilation at an Australian hospital, 2006–2012: a descriptive study. BMC Pregnancy Childbirth. 2016;16(1):328. 27793119 10.1186/s12884-016-1123-5 PMC5084319

[40] Rodriguez MI, Say L, Abdulcadir J, Hindin MJ. Clinical indications for cesarean delivery among women living with female genital mutilation. Int J Gynaecol Obstet. 2017;139(1):21–7. 28602042 10.1002/ijgo.12234 PMC5697596

[41] Balachandran AA, Duvalla S, Sultan AH, Thakar R. Are obstetric outcomes affected by female genital mutilation? Int Urogynecol J. 2018;29(3):339–44. 28889193 10.1007/s00192-017-3466-5

[42] Bohoussou K, Anongba S, Djanhan Y, Bonis S, Ble B, Sangaret M. Complications gynecologiques, medicales et obstetricales de l’excision rituelle. Afr Med. 1986;25:160–2.

[43] Berardi JC, Teillet JF, Godard J, Laloux V, Allane P, Franjou MH. Obstetrical consequences of female circumcision. Study in 71 circumcised African women. J Gynecol Obstet Biol Reprod (Paris). 1985;14(6):743–6. 3001171

[44] Davis G, Jellins J. Female genital mutilation: Obstetric outcomes in metropolitan Sydney. Aust N Z J Obstet Gynaecol. 2019;59(2):312–6. 30734267 10.1111/ajo.12954

[45] Mahmoud MIH. Effect of female genital mutilation on female sexual function, Alexandria Egypt. Alexandria J Med. 2016;52(1):55–9.

[46] Adinma JI. Current status of female circumcision among Nigerian Igbos. West Afr J Med. 1997;16(4):227–31. 9473958

[47] Yassin K, Idris HA, Ali AA. Characteristics of female sexual dysfunctions and obstetric complications related to female genital mutilation in Omdurman maternity hospital, Sudan. Reprod Health. 2018;15(1):7. 29310689 10.1186/s12978-017-0442-y PMC5759286

[48] Boghossian AS, Freebody J, Moses R, Jenkins G. Risk assessment for antenatal depression among women who have undergone female genital mutilation or cutting: Are we missing the mark? Aust N Z J Obstet Gynaecol. 2020;60(1):76–81. 31197824 10.1111/ajo.13001

[49] Akpak YK, Yilmaz I. The impact of female genital mutilation/cutting on obstetric outcomes and its management. J Matern Fetal Neonatal Med. 2022;35(5):927–32. 32131659 10.1080/14767058.2020.1734925

[50] Larsen U, Yan S. Does female circumcision affect infertility and fertility? A study of the central African Republic, Côte d’Ivoire, and Tanzania. Demography. 2000;37(3):313–21. 10953806

[51] Almroth L, Elmusharaf S, El Hadi N, Obeid A, El Sheikh MA, Elfadil SM, et al. Primary infertility after genital mutilation in girlhood in Sudan: a case-control study. Lancet. 2005;366(9483):385–91. 16054938 10.1016/S0140-6736(05)67023-7

[52] Larsen U. The effects of type of female circumcision on infertility and fertility in Sudan. J Biosoc Sci. 2002;34(3):363–77. 10.1017/s0021932002003632. 12117215 10.1017/s0021932002003632

[53] Okonofua FE, Larsen U, Oronsaye F, Snow RC, Slanger TE. The association between female genital cutting and correlates of sexual and gynaecological morbidity in Edo State. Nigeria BJOG. 2002;109(10):1089–96. 10.1111/j.1471-0528.2002.01550.x. PMID: 12387460. 12387460 10.1111/j.1471-0528.2002.01550.x

[54] Shandall AA. Circumcision and infibulation of females: a general consideration of the problem and a clinical study of the complications in Sudanese women. Sudan Med J. 1967;5(4):178–212. 12259304

[55] Yount KM, Abraham BK. Female genital cutting and HIV/AIDS among Kenyan women. Stud Fam Plann. 2007;38(2):73–88. 17642409 10.1111/j.1728-4465.2007.00119.x

[56] Maslovskaya O, Brown JJ, Padmadas SS. Disentangling the complex association between female genital cutting and HIV among Kenyan women. J Biosoc Sci. 2009;41(6):815–30. 19607733 10.1017/S0021932009990150

[57] Msuya SE, Mbizvo E, Hussain A, Sundby J, Sam NE, Stray-Pedersen B. Female genital cutting in Kilimanjaro, Tanzania: changing attitudes? Tropical Med Int Health. 2002;7(2):159–65. 10.1046/j.1365-3156.2002.00838.x 11841706

[58] Brewer DD, Potterat JJ, Roberts JM Jr, Brody S. Male and female circumcision associated with prevalent HIV infection in virgins and adolescents in Kenya, Lesotho, and Tanzania. Ann Epidemiol. 2007;17(3):217. e1-e12. 17320788 10.1016/j.annepidem.2006.10.010

[59] Pépin J, Plamondon M, Alves AC, Beaudet M, Labbé AC. Parenteral transmission during excision and treatment of tuberculosis and trypanosomiasis may be responsible for the HIV-2 epidemic in Guinea-Bissau. AIDS. 2006;20(9):1303–11. 16816560 10.1097/01.aids.0000232239.05545.33

[60] Kanki P, M’Boup S, Marlink R, Travers K, Hsieh CC, Gueye A, et al. Prevalence and risk determinants of human immunodeficiency virus type 2 (HIV-2) and human immunodeficiency virus type 1 (HIV-1) in west African female prostitutes. Am J Epidemiol. 1992;136(7):895–907. 1442755 10.1093/aje/136.7.895

[61] Holmgren B, da Silva Z, Larsen O, Vastrup P, Andersson S, Aaby P. Dual infections with HIV-1, HIV-2 and HTLV-I are more common in older women than in men in Guinea-Bissau. AIDS. 2003;17(2):241–53. 12545085 10.1097/00002030-200301240-00015

[62] Chu T, Akinsulure-Smith AM. Health outcomes and attitudes toward female genital cutting in a community-based sample of West African immigrant women from high-prevalence countries in New York City. J Aggress Maltreatment Trauma. 2016;25(1):63–83.

[63] Arafa AE, Elbahrawe RS, Shawky SM, Mostafa AM, Ahmed SS, El-Houfey A, et al. Risk factors associated with female sexual dysfunction among married women in Upper Egypt; a cross sectional study. Int J Community Med Public Health. 2018;5:449–53.

[64] Ashry Abd El Wahed Ali H, Arafa AE, Abd Allah NAEF, Fahim AS. Prevalence of female circumcision among young women in Beni-Suef, Egypt: a cross-sectional study. J Pediatr Adolesc Gynecol. 2018;31(6):571–4. 10.1016/j.jpag.2018.07.010 30064003

[65] El-Dafrawi M, Lotfy G, Dandash K, Refaat A, Eyada M. Female genital mutilation and its psychosocial impact. J Sex Marital Ther. 2001;27(5):453–8. 11554206 10.1080/713846799

[66] Shiferaw MT, Wubshet M, Tegabu D. Menstrual problems and associated factors among students of Bahir Dar University, Amhara National Regional State, Ethiopia: a cross-sectional survey. Pan Afr Med J. 2014;17:246. 10.11604/pamj.2014.17.246.2230 PMC4189866 25309646

[67] Matanda DJ, Sripad P, Ndwiga C. Is there a relationship between female genital mutilation/cutting and fistula? A statistical analysis using cross-sectional data from Demographic and Health Surveys in 10 sub-Saharan Africa countries. BMJ Open. 2019;9(7):e025355. 31362960 10.1136/bmjopen-2018-025355 PMC6678015

[68] Maheu-Giroux M, Filippi V, Maulet N, Samadoulougou S, Castro MC, Meda N, et al. Risk factors for vaginal fistula symptoms in Sub-Saharan Africa: a pooled analysis of national household survey data. BMC Pregnancy Childbirth. 2016;16:1–10. 27098261 10.1186/s12884-016-0871-6 PMC4839076

[69] Abdel-Aleem MA, Elkady MM, Hilmy YA. The relationship between female genital cutting and sexual problems experienced in the first two months of marriage. Int J Gynecol Obstet. 2016;132(3):305–8. 10.1016/j.ijgo.2015.07.030 26686766

[70] Oyefara JL. Female genital mutilation (FGM) and sexual functioning of married women in Oworonshoki Community, Lagos State. Nigeria Afr Popul Stud. 2015;29(1):1526–40.

[71] Odoi A, Brody S, Elkins T. Female genital mutilation in rural Ghana. West Africa Int J Gynaecol Obstet. 1997;56(2):179–80. 9061394 10.1016/s0020-7292(96)02800-7

[72] Ibrahim ZM, Ahmed MR, Ahmed WAS. Prevalence and risk factors for female sexual dysfunction among Egyptian women. Arch Gynecol Obstet. 2013;287:1173–80. 23274790 10.1007/s00404-012-2677-8

[73] Raheem TA, Eltahalawi SM, Raia NA, Elsary AY, Ibrahem KM. The effect of female genital mutilation on couple sexual function. Int J Commun Med Public Health. 2018;5(3):905–11.

[74] Alsibiani SA, Rouzi AA. Sexual function in women with female genital mutilation. Fertil Steril. 2010;93(3):722–4. 19028385 10.1016/j.fertnstert.2008.10.035

[75] Anis TH, Aboul Gheit S, Awad HH, Saied HS. Effects of female genital cutting on the sexual function of Egyptian women A cross-sectional study. J Sex Med. 2012;9(10):2682–92. 22897582 10.1111/j.1743-6109.2012.02866.x

[76] Mahmoudi O, Hosseini E. Psychosexual complications of female genital mutilation for couples: A comparative study. J Kermanshah Univ Med Sci. 2017;20(4):e69660.

[77] Obaid ZM, Amer AW-A, El Mahdy MAF, Mohammed AEB. Evaluation of Psychological and Sexual Effects of Female Genital Mutilation (Circumcision). Egypt J Hosp Med. 2019;74(4):726–34.

[78] Laleh S, Maleki A, Samiei V, Roshanaei G, Soltani F. The comparison of sexual function in women with or without experience of female genital circumcision: A case-control study in a Kurdish region of Iran. Health Care Women Int. 2022;43(1–3):194–206. 33797319 10.1080/07399332.2021.1887193

[79] Daneshkhah F, Allahverdipour H, Jahangiri L, Andreeva T. Sexual Function, Mental Well-being and Quality of Life among Kurdish Circumcised Women in Iran. Iran J Public Health. 2017;46(9):1265–74. 29026793 PMC5632329

[80] Biglu M-H, Farnam A, Abotalebi P, Biglu S, Ghavami M. Effect of female genital mutilation/cutting on sexual functions. Sex Reprod Healthc. 2016;10:3–8. 27938869 10.1016/j.srhc.2016.07.002

[81] Mohammed GF, Hassan MM, Eyada MM. Female Genital Mutilation/Cutting: Will It Continue? J Sex Med. 2014;11(11):2756–63. 25123710 10.1111/jsm.12655

[82] Birge O, Arslan D, Ozbey EG, Adiyeke M, Kayar I, Erkan MM, et al. Which type of circumcision is more harmful to female sexual functions? CEOG. 2017;44(5):691–4.

[83] Behrendt A, Moritz S. Posttraumatic stress disorder and memory problems after female genital mutilation. Am J Psychiatry. 2005;162(5):1000–2. 15863806 10.1176/appi.ajp.162.5.1000

[84] Applebaum J, Cohen H, Matar M, Abu Rabia Y, Kaplan Z. Symptoms of posttraumatic stress disorder after ritual female genital surgery among bedouin in Israel: myth or reality? Prim Care Companion J Clin Psychiatry. 2008;10(6):453–6. 19287554 10.4088/pcc.v10n0605 PMC2644466

[85] Piroozi B, Alinia C, Safari H, Kazemi-Karyani A, Moradi G, Farhadifar F, et al. Effect of female genital mutilation on mental health: a case–control study. Eur J Contracept Reprod Health Care. 2020;25(1):33–6. 31922433 10.1080/13625187.2019.1709815

[86] Abdullah FZ. The effect of female genital mutilation/cutting (FGM/C) on girls/women’s mental health: a case-control study in Kurdistan Region of Iraq. Arch Womens Ment Health. 2021;24(5):721–6. 33829321 10.1007/s00737-021-01125-4

[87] Jl K. Impact of psychological disorders after female genital mutilation among Kurdish girls in Northern Iraq. Eur J Psychiatr. 2011;25(2):92–100.

[88] Ahmed MR, Shaaban MM, Meky HK, Amin Arafa ME, Mohamed TY, Gharib WF, et al. Psychological impact of female genital mutilation among adolescent Egyptian girls: a cross-sectional study. Eur J Contracept Reprod Health Care. 2017;22(4):280–5. 28816075 10.1080/13625187.2017.1355454

[89] Institut National de la Statistique et de l'Analyse Économique (INSAE) et ORC Macro. 2002. Enquête Démographique et de Santé, Bénin 2001. Calverton, Maryland, USA: INSAE et ORC Macro International. https://microdata.worldbank.org/index.php/catalog/1329. Accessed 26 Dec 2024.

[90] Institut National de la Statistique et de l’Analyse Économique (INSAE) et Macro International Inc. 2007. Enquête Démographique et de Santé, Bénin 2006. Calverton, Maryland, USA: INSAE et Macro International. https://microdata.worldbank.org/index.php/catalog/1330. Accessed 26 Dec 2024.

[91] Institut National de la Statistique et de la Démographie (INSD) et ORC Macro. 2004. Enquête Démographique et de Santé, Burkina Faso 2003. Calverton, Maryland, USA: INSD et ORC Macro International. https://microdata.worldbank.org/index.php/catalog/1333. Accessed 26 Dec 2024.

[92] Institut National de la Statistique, des Études Économiques et Démographiques (INSEED) et ORC Macro. 2005. Enquête Démographique et de Santé, Tchad 2004. Calverton, Maryland, USA: INSEED et ORC Macro International. https://microdata.worldbank.org/index.php/catalog/1494. Accessed 26 Dec 2024.

[93] Direction Nationale de la Statistique et de l'Information (DNSI) et ORC Macro. 2000. Enquête Démographique et de Santé, Guinée 1999. Calverton, Maryland, USA: DNSI et ORC Macro International. https://microdata.worldbank.org/index.php/catalog/1389. Accessed 26 Dec 2024.

[94] Direction Nationale de la Statistique, Ministère du Plan [Guinea] et ORC Macro. 2006. Enquête Démographique et de Santé, Guinée 2005. Calverton, Maryland, USA: DNS et ORC Macro International. https://microdata.worldbank.org/index.php/catalog/1390. Accessed 26 Dec 2024.

[95] Cellule de Planification et de Statistique (CPS), Direction Nationale de la Statistique et de l'Informatique (DNSI) et ORC Macro. 2002. Enquête Démographique et de Santé, Mali 2001. Calverton, Maryland, USA: CPS, DNSI et ORC Macro International. https://microdata.worldbank.org/index.php/catalog/1441. Accessed 26 Dec 2024.

[96] Cellule de Planification et de Statistique (CPS), Direction Nationale de la Statistique et de l'Informatique (DNSI) et Macro International Inc. 2007. Enquête Démographique et de Santé, Mali 2006. Calverton, Maryland, USA: CPS, DNSI et Macro International. https://microdata.worldbank.org/index.php/catalog/1442. Accessed 26 Dec 2024.

[97] Office National de la Statistique (ONS) et ORC Macro. 2001. Enquête Démographique et de Santé, Mauritanie 2000–2001. Calverton, Maryland, USA: ONS et ORC Macro International. https://dhsprogram.com/publications/publication-FR127-DHS-Final-Reports.cfm. Accessed 26 Dec 2024.

[98] Centre de Recherche pour le Développement Humain (CRDH) et ORC Macro. 2006. Enquête Démographique et de Santé, Sénégal 2005. Calverton, Maryland, USA: CRDH et ORC Macro International. https://microdata.worldbank.org/index.php/catalog/1487. Accessed 26 Dec 2024.

[99] Central Statistical Organization (CSO) [Yemen] and Macro International Inc. 1998. Yemen Demographic and Maternal and Child Health Survey 1997. Calverton, Maryland, USA: CSO and Macro International. https://www.dhsprogram.com/publications/publication-FR94-DHS-Final-Reports.cfm. Accessed 26 Dec 2024.

[100] World Health Organization. Universal Health Coverage Compendium: Health Interventions for UHC. https://www.who.int/universal-health-coverage/compendium

[101] Tordrup D, Bishop C, Green N, Petzold M, Vallejo FR, Vogel JP, et al. Economic burden of female genital mutilation in 27 high-prevalence countries. BMJ Glob Health. 2022;7(2):e004512. 10.1136/bmjgh-2020-004512 PMC8744099 35105556

[102] O’Neill S, Pallitto C. The Consequences of Female Genital Mutilation on Psycho-Social Well-Being: A Systematic Review of Qualitative Research. Qual Health Res. 2021;31(9):1738–50. 34098783 10.1177/10497323211001862 PMC8438768

[103] Libretti A, Corsini C, Remorgida V. What’s new on female genital mutilation/cutting? Recent findings about urogynecologic complications, psychological issues, and obstetric outcomes. Minerva Obstet Gynecol. 2023. 10.23736/S2724-606X.23.05344-7. 37539674 10.23736/S2724-606X.23.05344-7

[104] Libretti A, Bianco G, Corsini C, Remorgida V. Female Genital Mutilation/Cutting: an urgent call to action. Acta Biomed. 2023;94(2):e2023064. 10.23750/abm.v94i2.14205. PMID:37092620;PMCID:PMC10210570. 37092620 10.23750/abm.v94i2.14205 PMC10210570

