# Supplementary material for: Exploring the health complications of female genital mutilation through a systematic review and meta-analysis
Source: BMC Public Health. 2025 Apr 14;25:1387. doi: 10.1186/s12889-025-21584-z (PMC11995580; doi:10.1186/s12889-025-21584-z)
Supplement: Supplementary file 1 — Supplementary Material 1. [file 12889_2025_21584_MOESM1_ESM.pdf]

Database	Search terms
African Index Medicus	“female genital mutilation” OR “female genital cutting”
Anthropology plus	((kw: female* or kw: wom#n) or kw: girl*) and ((kw: mutilation* or kw: infibulate*) or kw: cutting*) or kw: infibulate*
CINAHL	(MH ”Circumcision, Female”) OR (TI ((female* or wom#n or girl*) N3 (mutilation* or circumcis* or cutting*)) OR AB ((female* or wom#n or girl*) N3 (mutilation* or circumcis* or cutting*))) OR (TI "fgm/c" OR AB "fgm/c") OR (TI ((removal* or alteration* or excision*) N6 (female W0 genital*)) OR AB ((removal* or alteration* or excision*) N6 (female W0 genital*))) OR (TI pharaonic W0 circumcision* OR AB pharaonic W0 circumcision*) OR (TI (sunna or clitoridectom* or clitorectom* or infibulat* reinfibulat* or deinfibulat*) OR AB (sunna or clitoridectom* or clitorectom* or inbibulat*reinfibulat* or deinfibulat*))
The Cochrane Library Databases in The Cochrane Library:<br>• Cochrane Database of Systematic Reviews (CDSR): Issue 12 of 12, Dec 2011 • Cochrane Central Register of Controlled Trials (CENTRAL),<br>• Database of Abstracts of Reviews of Effects (DARE)<br>• Health Technology Assessment Database (HTA): Issue 4 of 4 Oct 2011	((female* or woman or women or girl or girls) near/3 (mutilation* or circumcis* or cutting*)) in Title, Abstract, Keywords or "fgm/c" or ((removal* or alteration* or excision*) near/6 (female next genital*)) and (pharaonic next circumcision) or sunna or clitoridectom* or clitorectom* or infibulat* or reinfibulat* or deinfibulat* and ((female* or woman or women or girl or girls) near/3 (mutilation* or circumcis* or cutting*)) and "fgm/c" or ((removal* or alteration* or excision*) near/6 (female next genital*)) or (pharaonic next circumcision*) , Online Publication Date from Feb 2012 in Cochrane Reviews'
EMBASE (Ovid EMBASE)	'genital mutilation' OR 'female genital mutilation'/exp OR 'female genital mutilation' OR (removal* OR alteration* OR excision* AND within AND 6 AND ('female'/exp OR female) AND genital*) OR (pharaonic AND circumcision*) OR 'sunna' AND ('tissues'/exp OR 'tissues' OR 'ureteral obstruction'/exp OR 'ureteral obstruction' OR 'urethral obstruction'/exp OR 'urethral obstruction' OR 'cysts'/exp OR 'cysts' OR 'urination disorders'/exp OR 'urination disorders' OR 'menstruation disturbances'/exp OR 'menstruation disturbances' OR 'coitus'/exp OR 'coitus' OR 'reproductive tract infections'/exp OR 'reproductive tract infections' OR 'urinary tract infections'/exp OR 'urinary tract infections' OR 'hiv'/exp OR 'hiv' OR 'sexually transmitted' OR 'infertility'/exp OR 'infertility' OR 'vaginal discharge'/exp OR 'vaginal discharge' OR 'pruritus'/exp OR 'pruritus' OR 'infection'/exp OR 'infection' OR 'fever'/exp OR 'fever' OR 'hemorrhage'/exp OR 'hemorrhage' OR 'shock'/exp OR 'shock' OR 'hemorrhagic' OR 'labor'/exp OR 'labor' OR 'obstetric' OR 'lacerations'/exp OR 'lacerations' OR 'cesarean' OR 'episiotomy'/exp OR 'episiotomy' OR 'delivery'/exp OR 'delivery' OR 'postpartum hemorrhage'/exp OR 'postpartum hemorrhage' OR 'dystocia'/exp OR 'dystocia' OR 'stress disorders' OR 'post-traumatic' OR 'anxiety'/exp OR 'anxiety' OR 'somatoform disorders'/exp OR 'somatoform disorders' OR 'depression'/exp OR 'depression' OR 'hostility'/exp OR 'hostility' OR 'quality of life'/exp OR 'quality of life' OR 'dyspareunia'/exp OR 'dyspareunia' OR 'personal satisfaction'/exp OR 'personal satisfaction' OR 'libido'/exp OR 'libido' OR 'orgasm'/exp OR 'orgasm') AND [2017-2019]/py

MEDLINE	• FGM/C+Gynaecological consequences((((((((((clitoridectom*) OR clitorectom*)) OR "sunna") OR pharaonis circumcision*) OR ((((removal*) OR alteration*) OR excision*) AND female genital*)) OR (("fgm") OR "fgc")) OR ((((((female*) OR wom*) OR girl*) AND mutilation*) OR circumcis*) OR cutting*)) OR Circumcision, Female[MeSH Major Topic]) OR (((infibulat*) OR reinfibulat*) OR deinfibulat*))) AND ("Tissues" OR "Ureteral Obstruction" OR "Urethral Obstruction" OR "Cysts" OR "Urination Disorders" OR "Menstruation Disturbances" OR "Coitus" OR "Reproductive Tract Infections" OR "Urinary Tract Infections" OR "HIV" OR "Sexually Transmitted Diseases" OR "Infertility" OR "Vaginal Discharge" OR "Pruritus" OR "Infection" OR "Fever" OR "Hemorrhage" OR "Shock, Hemorrhagic" OR "Postoperative Hemorrhage" OR "Uterine Hemorrhage" AND ("2017/06/01"[PDAT] : "3000/12/31"[PDAT]))• FGM/C+ Obstetric consequences((((((((((clitoridectom*) OR clitorectom*)) OR "sunna") n pharaonis circumcision*) OR ((((removal*) OR alteration*) OR excision*) AND female genital*)) OR (("fgm") OR "fgc")) OR ((((((female*) OR wom*) OR girl*) AND mutilation*) OR circumcis*) OR cutting*)) OR Circumcision, Female[MeSH Major Topic]) OR (((infibulat*) OR reinfibulat*) OR deinfibulat*))) AND ("Labor, Obstetric" OR "Lacerations" OR "Cesarean Section" OR "Episiotomy" OR "Delivery, Obstetric" OR "Postpartum Hemorrhage" OR "Dystocia" AND ("2017/06/01"[PDAT] : "3000/12/31"[PDAT]))• FGM/C+ Psychological consequences((((((((((clitoridectom*) OR clitorectom*)) OR "sunna") OR pharaonis circumcision*) OR ((((removal*) OR alteration*) OR excision*) AND female genital*)) OR (("fgm") OR "fgc")) OR ((((((female*) OR wom*) OR girl*) AND mutilation*) OR circumcis*) OR cutting*)) OR Circumcision, Female[MeSH Major Topic]) OR (((infibulat*) OR reinfibulat*) OR deinfibulat*))) AND ("Stress Disorders, Post-Traumatic" OR "Anxiety" OR "Somatoform Disorders" OR "Depression" OR "Hostility" OR "Quality of Life" AND ("2017/06/01"[PDAT] : "3000/12/31"[PDAT]))• FGM/C+ Sexual consequences((((((((((clitoridectom*) OR clitorectom*)) OR "sunna") n pharaonis circumcision*) OR ((((removal*) OR alteration*) OR excision*) AND female genital*)) OR (("fgm") OR "fgc")) OR ((((((female*) OR wom*) OR girl*) AND mutilation*) OR circumcis*) OR cutting*)) OR Circumcision, Female[MeSH Major Topic]) OR (((infibulat*) OR reinfibulat*) OR deinfibulat*))) AND ("Dyspareunia" OR "Personal Satisfaction" OR "Libido" OR "Orgasm" AND ("2017/06/01"[PDAT] : "3000/12/31"[PDAT]))
PILOTS	(("genital mutilation") OR ("female genital mutilation") OR ((removal* OR alteration* OR excision*) within 6 (female genital*)) OR (pharaonic circumcision*) OR ("sunna")) AND (("Tissues") OR ("Ureteral Obstruction") OR ("Urethral Obstruction") OR ("Cysts") OR ("Urination Disorders") OR ("Menstruation Disturbances") OR ("Coitus") OR ("Reproductive Tract Infections") OR ("Urinary Tract Infections") OR ("shiv") OR ("Sexually Transmitted") OR ("Infertility") OR ("Vaginal Discharge") OR ("Pruritus") OR ("Infection") OR ("Fever") OR ("Hemorrhage") OR ("Shock") OR ("Hemorrhagic") OR ("Labor") OR ("Obstetric") OR ("Lacerations") OR ("Cesarean") OR ("Episiotomy") OR ("Delivery") OR ("Postpartum Hemorrhage") OR ("Dystocia") OR ("Stress Disorders") OR ("Post-Traumatic") OR ("Anxiety") OR ("somatology Disorders") OR ("Depression") OR ("Hostility") OR ("Quality of Life") OR ("Dyspareunia") OR ("Personal Satisfaction") OR ("Libido") OR ("Orgasm"))
POPLINE	(((((((female genital mutilation OR female genital cutting) AND (effects OR consequences OR problems))))))) AND ((Added to POPLINE:[2017-06-01T22:00:00.000Z TO *]))

PsycINFO	1. Circumcision/<br>2. ((female$ or wom#n or girl$1) adj3 (mutilation$ or circumcis$ or cutting$)).tw. 3. "fgm/c".tw.<br>4. ((removal$ or alteration$ or excision$) adj6 female genital$).tw.<br>5. pharaonic circumcision$.tw.<br>6. sunna.tw.<br>7. (clitoridectom$ or clitorectom$).tw.<br>8. (infibulat$ or reinfibulat$ or deinfibulat$).tw.<br>9. or/1-8
Social Services Abstracts	su.EXACT("Genital Mutilation" OR "Circumcision") OR ti((female* NEAR/3 (mutilation* OR circumcis* OR cutting*))) OR ab((female* NEAR/3 (mutilation* OR circumcis* OR cutting*)))
Sociological Abstracts	su.EXACT("Genital Mutilation" OR "Circumcision") OR ti((female* NEAR/3 (mutilation* OR circumcis* OR cutting*))) OR ab((female* NEAR/3 (mutilation* OR circumcis* OR cutting*)))
WHOLIS	words or phrase "((female$ or wom?n or girl or girls) near3 (mutilation$ or circum- cis$ or cutting$))"<br>OR<br>words or phrase ""fgm/c""<br>OR<br>words or phrase "((removal$ or alteration$ or excision$) near6 (female adj geni- tal$))"<br>OR<br>words or phrase "(pharaonic adj circumcision$)"<br>OR<br>words or phrase "sunna"<br>OR<br>words or phrase "(clitoridectom$ or clitorectom$)"<br>OR<br>words or phrase "(infibulat$ or reinfibulat$ or deinfibulat$)"
Scopus	TITLE-ABS-KEY (("genital mutilation") OR ("female genital mutilation") OR ((removal* OR alteration* OR excision*) within 6 (female genital*)) OR (pharaonic circumcision*) OR ("sunna")) AND (("Tissues") OR ("Ureteral Obstruction") OR ("Urethral Obstruction") OR ("Cysts") OR ("Urination Disorders") OR ("Menstruation Disturbances") OR ("Coitus") OR ("Reproductive Tract Infections") OR ("Urinary Tract Infections") OR ("HIV") OR ("Sexually Transmitted") OR ("Infertility") OR ("Vaginal Discharge") OR ("Pruritus") OR ("Infection") OR ("Fever") OR ("Hemorrhage") OR ("Shock") OR ("Hemorrhagic") OR ("Labor") OR ("Obstetric") OR ("Lacerations") OR ("Cesarean") OR ("Episiotomy") OR ("Delivery") OR ("Postpartum Hemorrhage") OR ("Dystocia") OR ("Stress Disorders") OR ("Post-Traumatic") OR ("Anxiety") OR ("Somatoform Disorders") OR ("Depression") OR ("Hostility") OR ("Quality of Life") OR ("Dyspareunia") OR ("Personal Satisfaction") OR ("Libido") OR ("Orgasm")) AND (LIMIT-TO (PUBYEAR , 2017) OR LIMIT-TO (PUBYEAR , 2018) OR LIMIT-TO (PUBYEAR , 2019)
Index Medicus for Eastern Mediterranean Region	“female genital mutilation” OR “female genital cutting”
RHS / DHS website	“female genital mutilation” OR “female genital cutting”
