# Supplementary material for: Exploring the health complications of female genital mutilation through a systematic review and meta-analysis
Source: BMC Public Health. 2025 Apr 14;25:1387. doi: 10.1186/s12889-025-21584-z (PMC11995580; doi:10.1186/s12889-025-21584-z)

Prolonged or obstructed labour, Any FGM

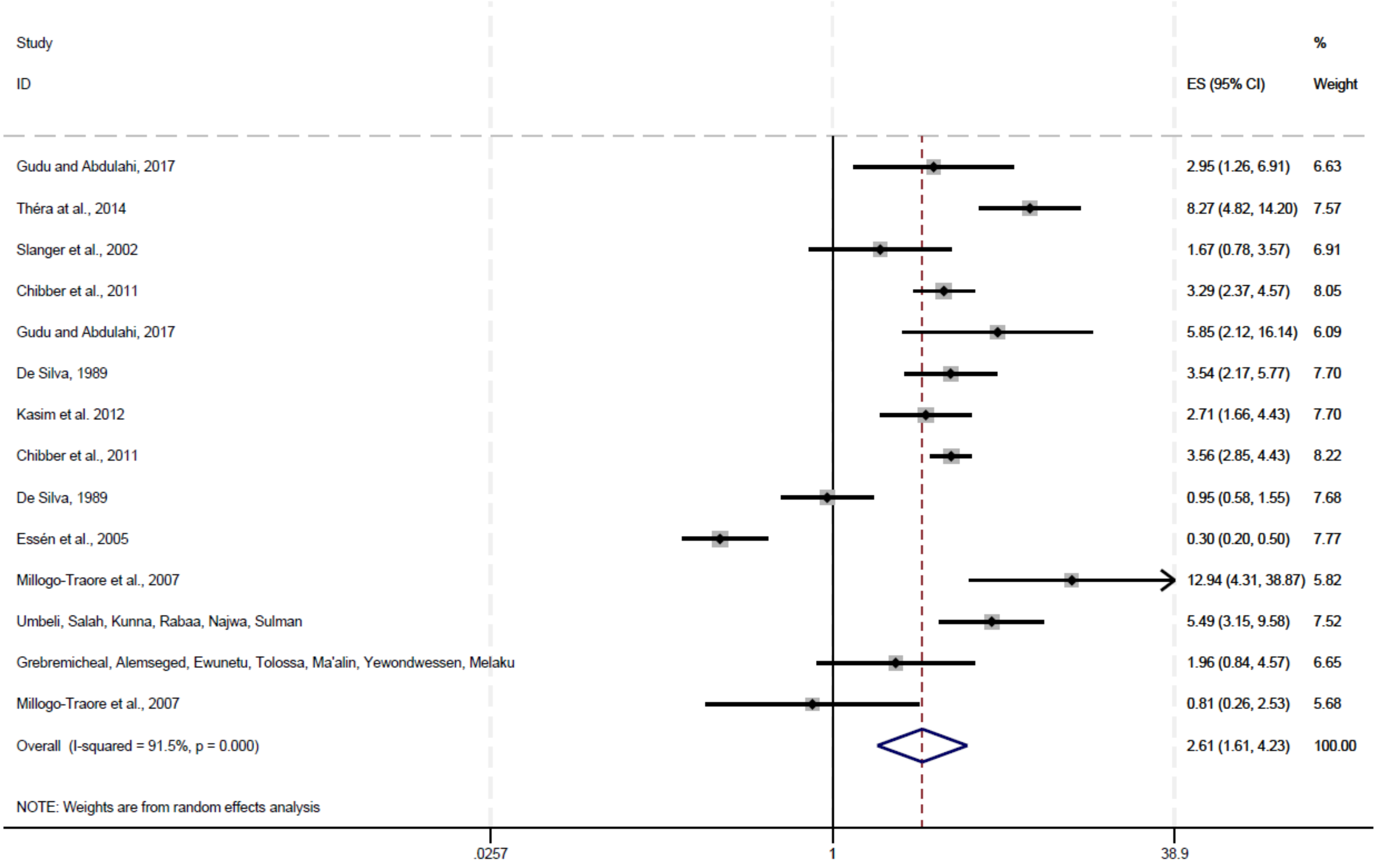

Prolonged or obstructed labour, Type I or II FGM

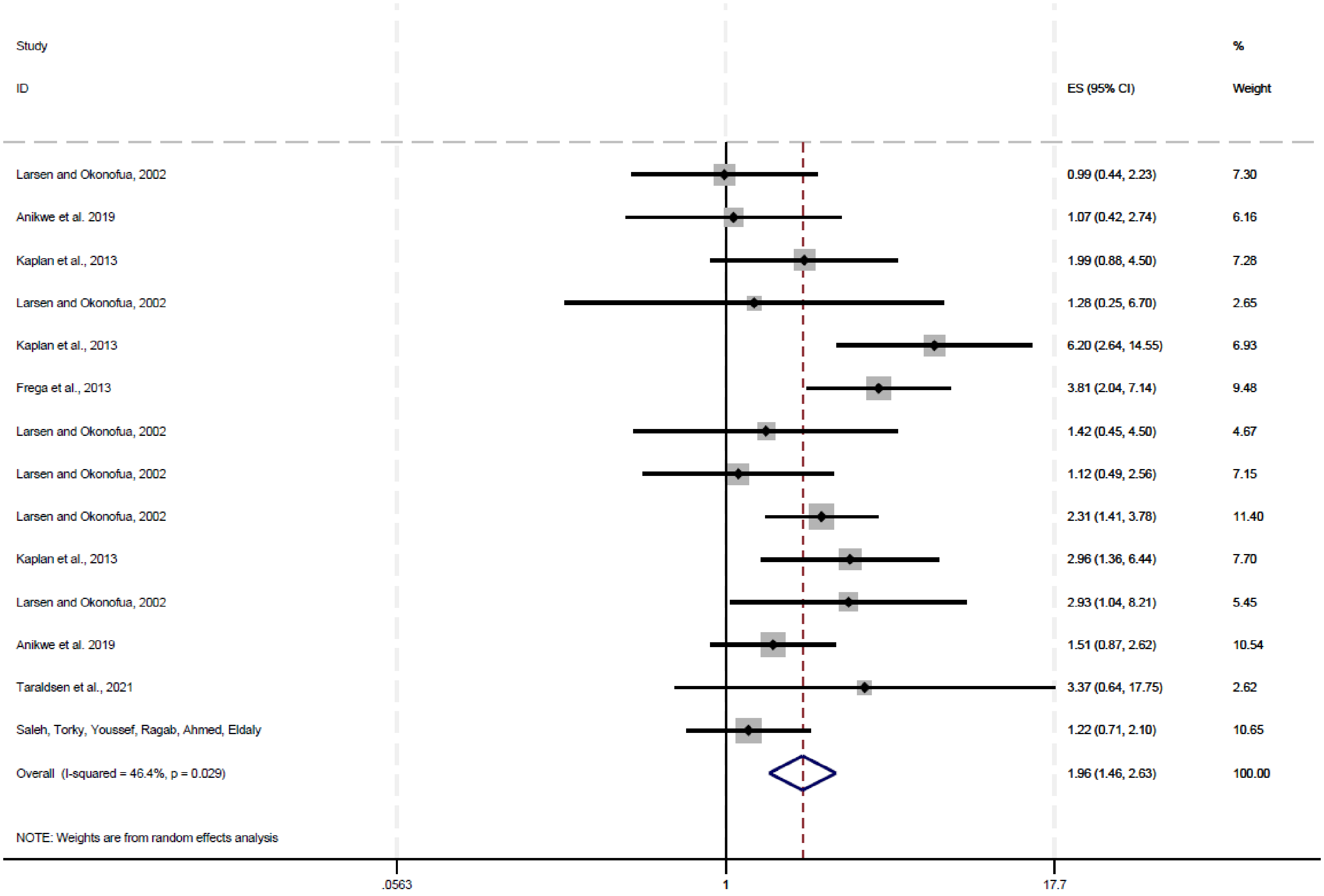

Stillbirth or neonatal death, Any FGM

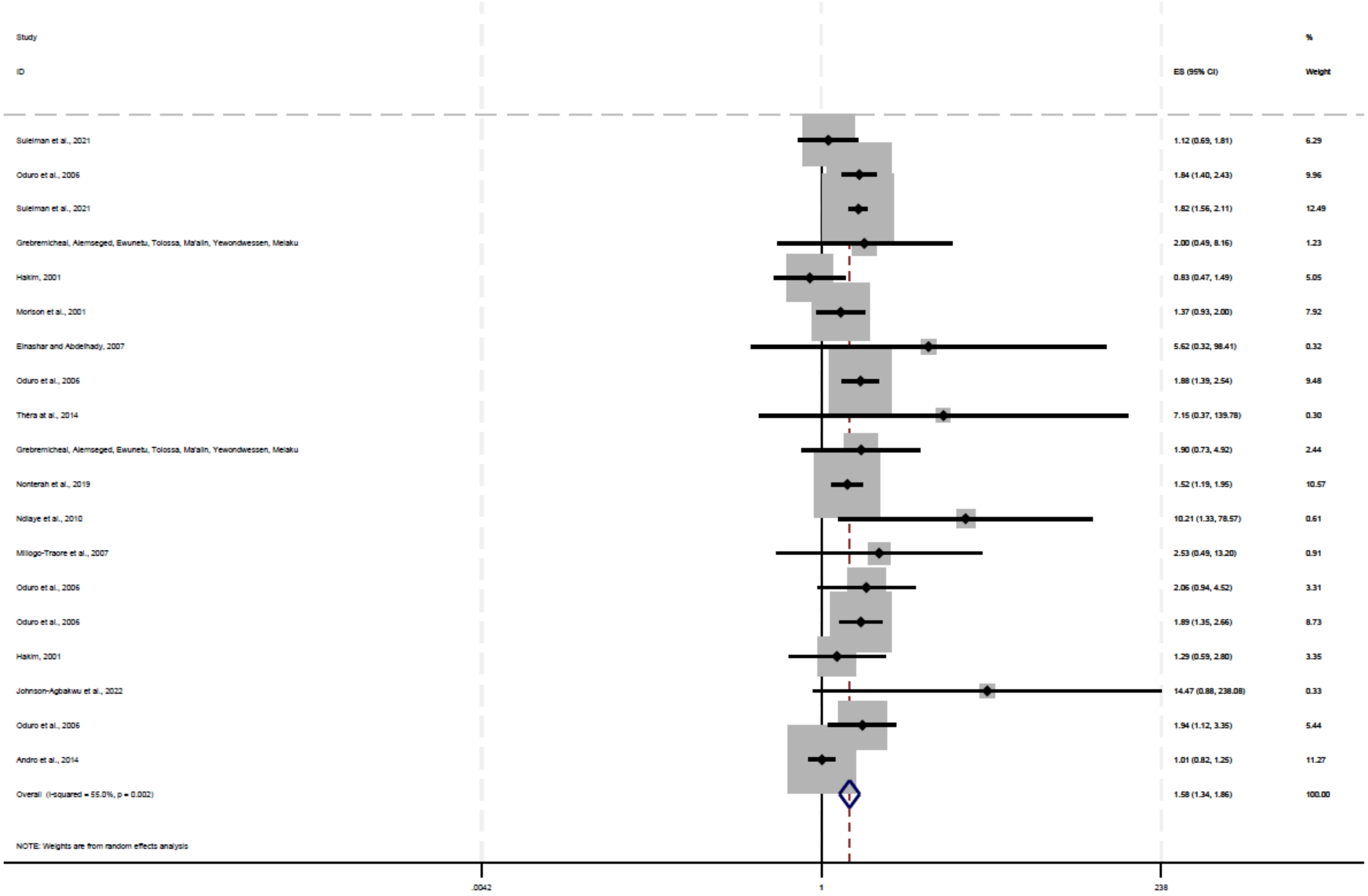

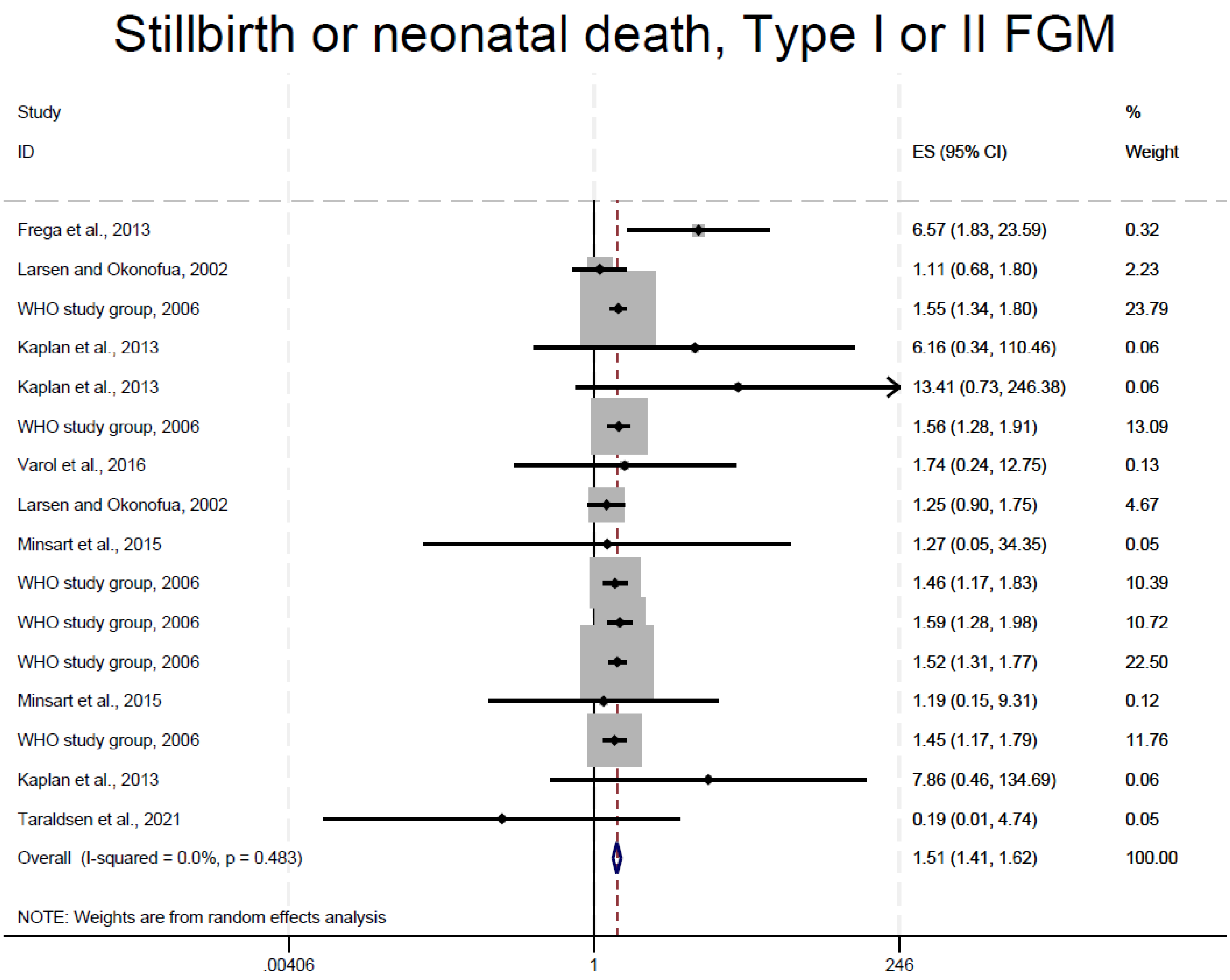
Stillbirth or neonatal death, Type I or II FGM
Study
ID
ES (95% CI)
%
Weight
Frega et al., 2013
6.57 (1.83, 23.59)
0.32
Larsen and Okonofua, 2002
1.11 (0.68, 1.80)
2.23
WHO study group, 2006
1.55 (1.34, 1.80)
23.79
Kaplan et al., 2013
6.16 (0.34, 110.46)
0.06
Kaplan et al., 2013
13.41 (0.73, 246.38)
0.06
WHO study group, 2006
1.56 (1.28, 1.91)
13.09
Varol et al., 2016
1.74 (0.24, 12.75)
0.13
Larsen and Okonofua, 2002
1.25 (0.90, 1.75)
4.67
Minsart et al., 2015
1.27 (0.05, 34.35)
0.05
WHO study group, 2006
1.46 (1.17, 1.83)
10.39
WHO study group, 2006
1.59 (1.28, 1.98)
10.72
WHO study group, 2006
1.52 (1.31, 1.77)
22.50
Minsart et al., 2015
1.19 (0.15, 9.31)
0.12
WHO study group, 2006
1.45 (1.17, 1.79)
11.76
Kaplan et al., 2013
7.86 (0.46, 134.69)
0.06
Taraldsen et al., 2021
0.19 (0.01, 4.74)
0.05
Overall (I-squared = 0.0%, p = 0.483)
1.51 (1.41, 1.62)
100.00
NOTE: Weights are from random effects analysis
.00406
1
246

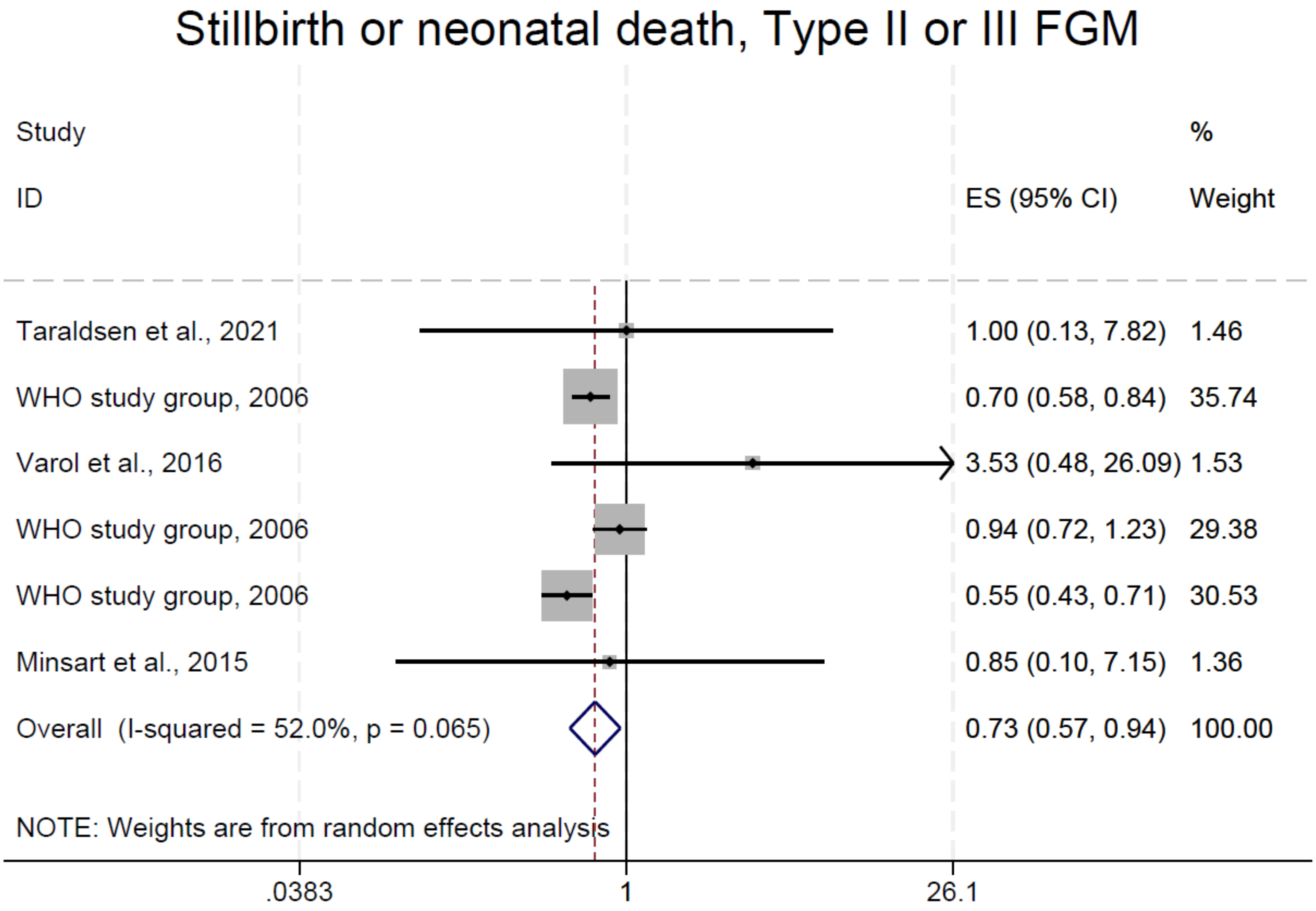
Stillbirth or neonatal death, Type II or III FGM
Study
ID
ES (95% CI)
%
Weight
Taraldsen et al., 2021
1.00 (0.13, 7.82)
1.46
WHO study group, 2006
0.70 (0.58, 0.84)
35.74
Varol et al., 2016
3.53 (0.48, 26.09)
1.53
WHO study group, 2006
0.94 (0.72, 1.23)
29.38
WHO study group, 2006
0.55 (0.43, 0.71)
30.53
Minsart et al., 2015
0.85 (0.10, 7.15)
1.36
Overall (I-squared = 52.0%, p = 0.065)
0.73 (0.57, 0.94)
100.00
NOTE: Weights are from random effects analysis
.0383
1
26.1

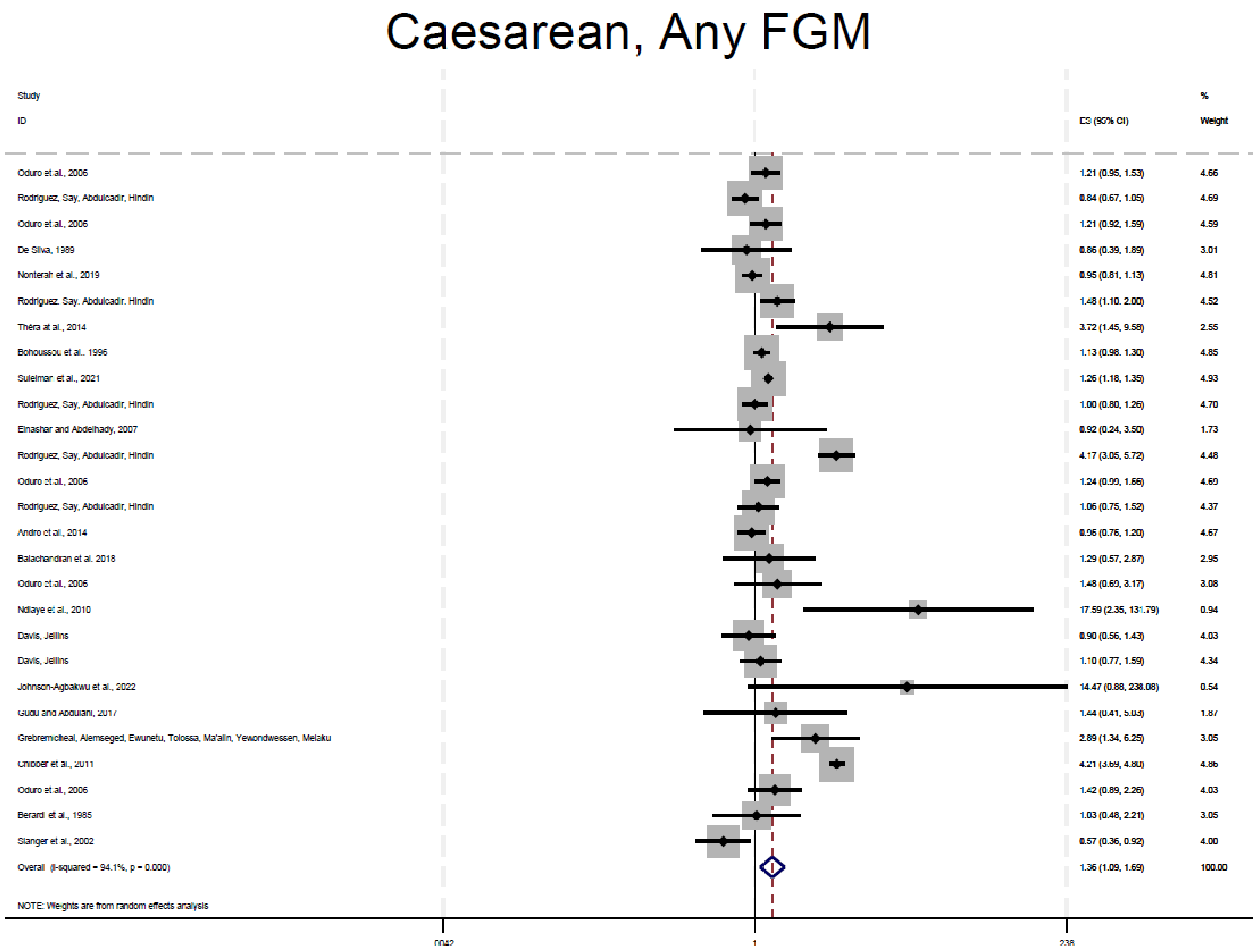
Caesarean, Any FGM
Study
ID
ES (95% CI)
%
Weight
Oduro et al., 2006 1.21 (0.95, 1.53) 4.66
Rodriguez, Say, Abdulcadir, Hindin 0.84 (0.67, 1.05) 4.69
Oduro et al., 2006 1.21 (0.92, 1.59) 4.59
De Silva, 1989 0.86 (0.39, 1.89) 3.01
Nonterah et al., 2019 0.95 (0.81, 1.13) 4.81
Rodriguez, Say, Abdulcadir, Hindin 1.48 (1.10, 2.00) 4.52
Théra at al., 2014 3.72 (1.45, 9.58) 2.55
Bohoussou et al., 1996 1.13 (0.98, 1.30) 4.85
Suleiman et al., 2021 1.26 (1.18, 1.35) 4.93
Rodriguez, Say, Abdulcadir, Hindin 1.00 (0.80, 1.26) 4.70
Elnashar and Abdelhady, 2007 0.92 (0.24, 3.50) 1.73
Rodriguez, Say, Abdulcadir, Hindin 4.17 (3.05, 5.72) 4.48
Oduro et al., 2006 1.24 (0.99, 1.56) 4.69
Rodriguez, Say, Abdulcadir, Hindin 1.06 (0.75, 1.52) 4.37
Andro et al., 2014 0.95 (0.75, 1.20) 4.67
Balachandran et al. 2018 1.29 (0.57, 2.87) 2.95
Oduro et al., 2006 1.48 (0.69, 3.17) 3.08
Ndiaye et al., 2010 17.59 (2.35, 131.79) 0.94
Davis, Jellins 0.90 (0.56, 1.43) 4.03
Davis, Jellins 1.10 (0.77, 1.59) 4.34
Johnson-Agbakwu et al., 2022 14.47 (0.88, 238.08) 0.54
Gudu and Abdulahi, 2017 1.44 (0.41, 5.03) 1.87
Gebremicheal, Alemseged, Ewunetu, Tolossa, Ma'alin, Yewondwessen, Melaku 2.89 (1.34, 6.25) 3.05
Chibber et al., 2011 4.21 (3.69, 4.80) 4.86
Oduro et al., 2006 1.42 (0.89, 2.26) 4.03
Berardi et al., 1985 1.03 (0.48, 2.21) 3.05
Slanger et al., 2002 0.57 (0.36, 0.92) 4.00
Overall (I-squared = 94.1%, p = 0.000) 1.36 (1.09, 1.69) 100.00
NOTE: Weights are from random effects analysis
.0042
1
238

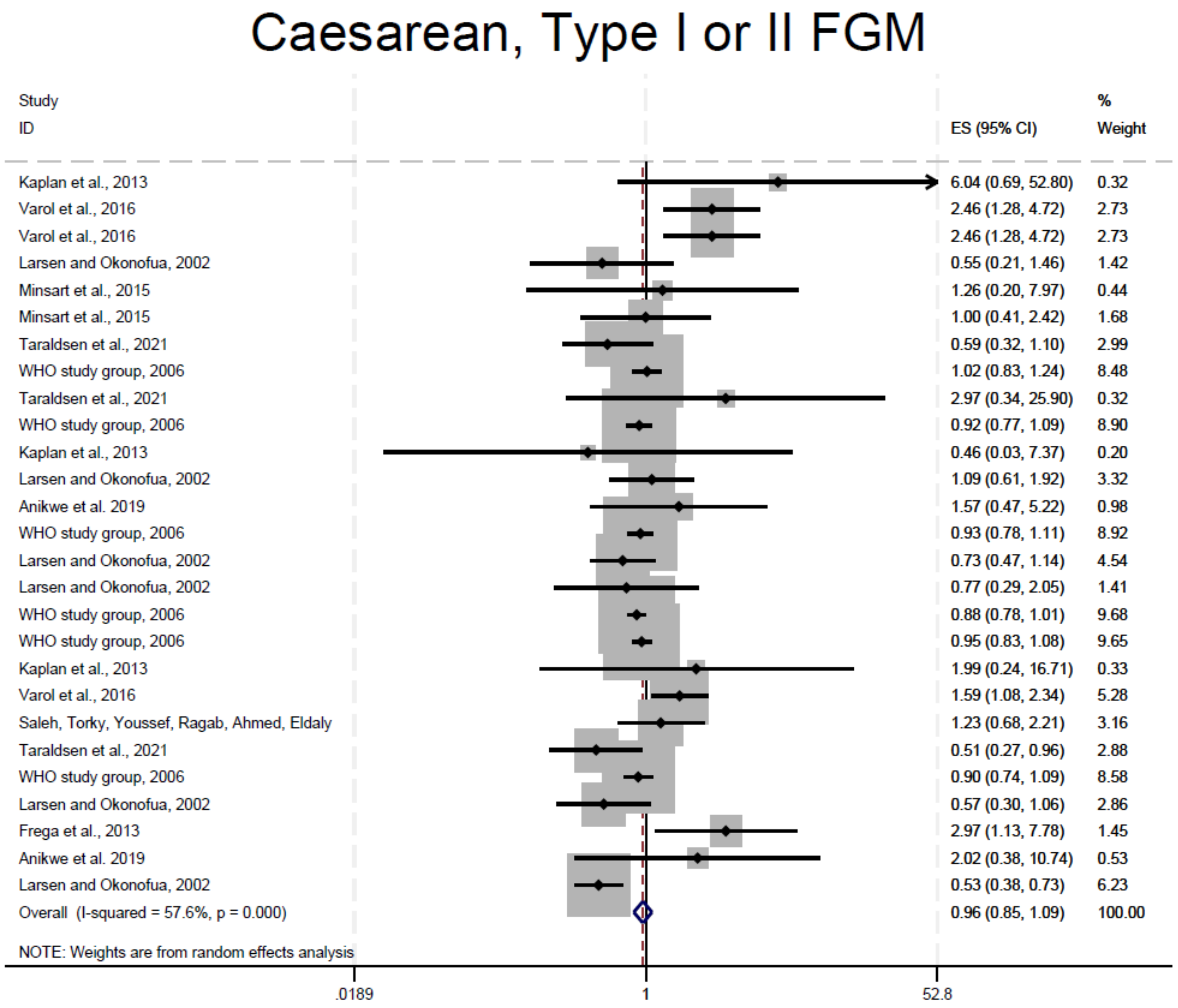
Caesarean, Type I or II FGM
Study
ID
ES (95% CI)
%
Weight
Kaplan et al., 2013 6.04 (0.69, 52.80) 0.32
Varol et al., 2016 2.46 (1.28, 4.72) 2.73
Varol et al., 2016 2.46 (1.28, 4.72) 2.73
Larsen and Okonofua, 2002 0.55 (0.21, 1.46) 1.42
Minsart et al., 2015 1.26 (0.20, 7.97) 0.44
Minsart et al., 2015 1.00 (0.41, 2.42) 1.68
Taraldsen et al., 2021 0.59 (0.32, 1.10) 2.99
WHO study group, 2006 1.02 (0.83, 1.24) 8.48
Taraldsen et al., 2021 2.97 (0.34, 25.90) 0.32
WHO study group, 2006 0.92 (0.77, 1.09) 8.90
Kaplan et al., 2013 0.46 (0.03, 7.37) 0.20
Larsen and Okonofua, 2002 1.09 (0.61, 1.92) 3.32
Anikwe et al. 2019 1.57 (0.47, 5.22) 0.98
WHO study group, 2006 0.93 (0.78, 1.11) 8.92
Larsen and Okonofua, 2002 0.73 (0.47, 1.14) 4.54
Larsen and Okonofua, 2002 0.77 (0.29, 2.05) 1.41
WHO study group, 2006 0.88 (0.78, 1.01) 9.68
WHO study group, 2006 0.95 (0.83, 1.08) 9.65
Kaplan et al., 2013 1.99 (0.24, 16.71) 0.33
Varol et al., 2016 1.59 (1.08, 2.34) 5.28
Saleh, Torky, Youssef, Ragab, Ahmed, Eldaly 1.23 (0.68, 2.21) 3.16
Taraldsen et al., 2021 0.51 (0.27, 0.96) 2.88
WHO study group, 2006 0.90 (0.74, 1.09) 8.58
Larsen and Okonofua, 2002 0.57 (0.30, 1.06) 2.86
Frega et al., 2013 2.97 (1.13, 7.78) 1.45
Anikwe et al. 2019 2.02 (0.38, 10.74) 0.53
Larsen and Okonofua, 2002 0.53 (0.38, 0.73) 6.23
Overall (I-squared = 57.6%, p = 0.000) 0.96 (0.85, 1.09) 100.00
NOTE: Weights are from random effects analysis
.0189
1
52.8

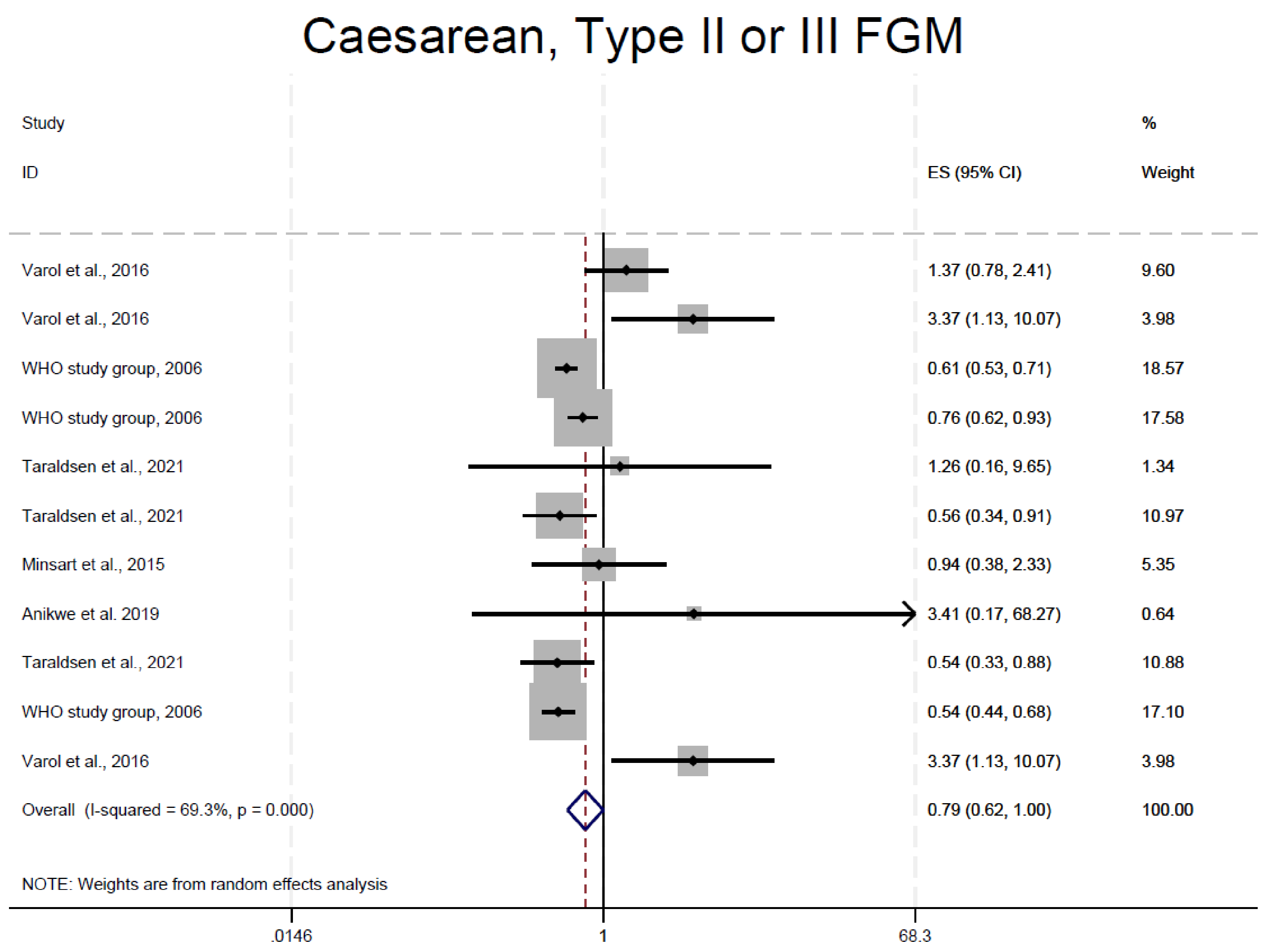

Caesarean, Type II or III FGM
Study
ID
ES (95% CI)
%
Weight
Varol et al., 2016 1.37 (0.78, 2.41) 9.60
Varol et al., 2016 3.37 (1.13, 10.07) 3.98
WHO study group, 2006 0.61 (0.53, 0.71) 18.57
WHO study group, 2006 0.76 (0.62, 0.93) 17.58
Taraldsen et al., 2021 1.26 (0.16, 9.65) 1.34
Taraldsen et al., 2021 0.56 (0.34, 0.91) 10.97
Minsart et al., 2015 0.94 (0.38, 2.33) 5.35
Anikwe et al. 2019 3.41 (0.17, 68.27) 0.64
Taraldsen et al., 2021 0.54 (0.33, 0.88) 10.88
WHO study group, 2006 0.54 (0.44, 0.68) 17.10
Varol et al., 2016 3.37 (1.13, 10.07) 3.98
Overall (I-squared = 69.3%, p = 0.000) 0.79 (0.62, 1.00) 100.00
NOTE: Weights are from random effects analysis
.0146
1
68.3

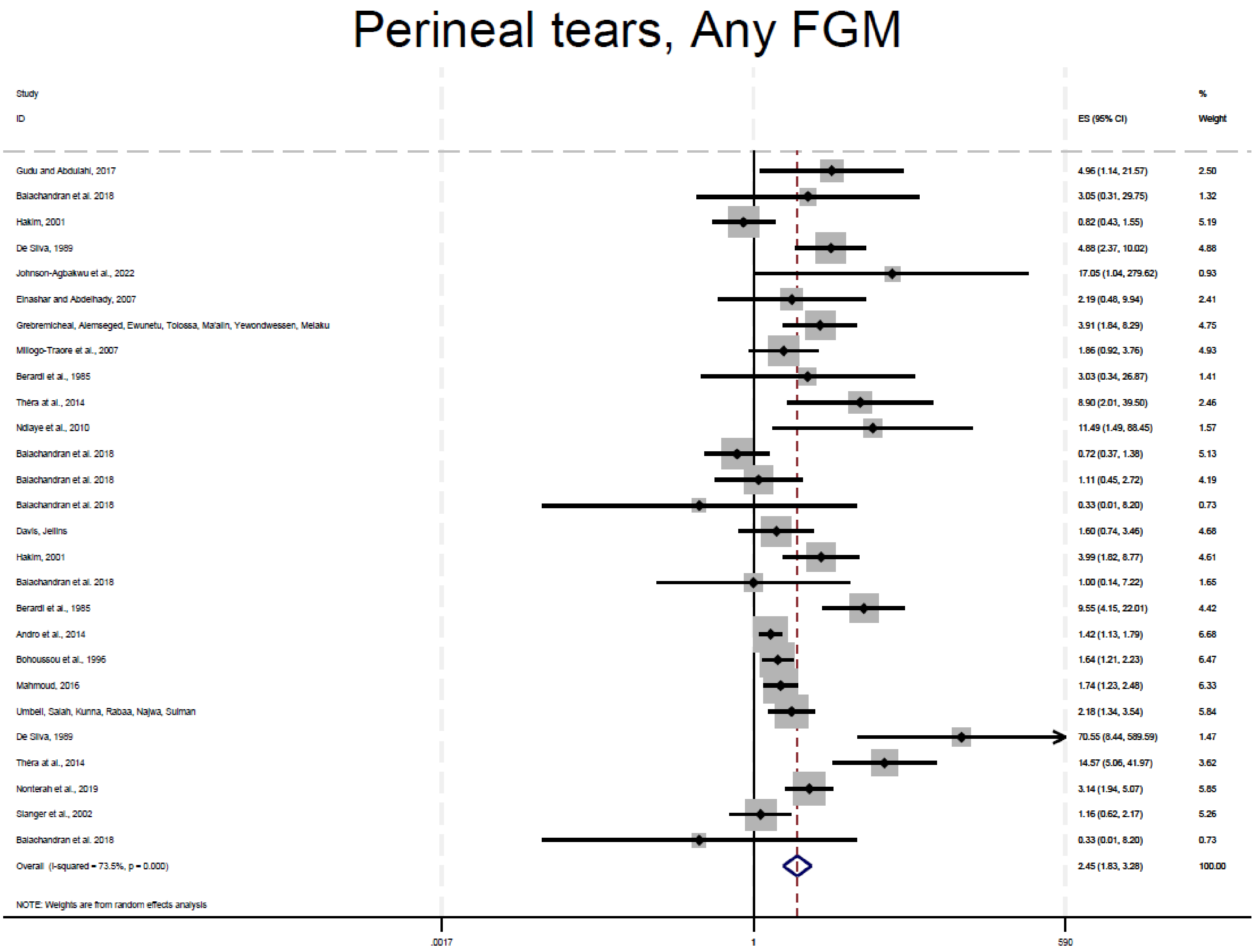
Perineal tears, Any FGM
Study
ID
ES (95% CI)
%
Weight
Gudu and Abdulahi, 2017 4.96 (1.14, 21.57) 2.50
Balachandran et al. 2018 3.05 (0.31, 29.75) 1.32
Hakim, 2001 0.82 (0.43, 1.55) 5.19
De Silva, 1989 4.88 (2.37, 10.02) 4.88
Johnson-Agbakwu et al., 2022 17.05 (1.04, 279.62) 0.93
Elnashar and Abdelhady, 2007 2.19 (0.48, 9.94) 2.41
Grebremicheal, Alemseged, Ewunetu, Tolossa, Ma'alin, Yewondwessen, Melaku 3.91 (1.84, 8.29) 4.75
Millogo-Traore et al., 2007 1.86 (0.92, 3.76) 4.93
Berardi et al., 1985 3.03 (0.34, 26.87) 1.41
Théra at al., 2014 8.90 (2.01, 39.50) 2.46
Ndiaye et al., 2010 11.49 (1.49, 88.45) 1.57
Balachandran et al. 2018 0.72 (0.37, 1.38) 5.13
Balachandran et al. 2018 1.11 (0.45, 2.72) 4.19
Balachandran et al. 2018 0.33 (0.01, 8.20) 0.73
Davis, Jellins 1.60 (0.74, 3.46) 4.68
Hakim, 2001 3.99 (1.82, 8.77) 4.61
Balachandran et al. 2018 1.00 (0.14, 7.22) 1.65
Berardi et al., 1985 9.55 (4.15, 22.01) 4.42
Andro et al., 2014 1.42 (1.13, 1.79) 6.68
Bohoussou et al., 1996 1.64 (1.21, 2.23) 6.47
Mahmoud, 2016 1.74 (1.23, 2.48) 6.33
Umbeli, Salah, Kunna, Rabaa, Najwa, Sulman 2.18 (1.34, 3.54) 5.84
De Silva, 1989 70.55 (8.44, 589.59) 1.47
Théra at al., 2014 14.57 (5.06, 41.97) 3.62
Nonterah et al., 2019 3.14 (1.94, 5.07) 5.85
Slanger et al., 2002 1.16 (0.62, 2.17) 5.26
Balachandran et al. 2018 0.33 (0.01, 8.20) 0.73
Overall (I-squared = 73.5%, p = 0.000) 2.45 (1.83, 3.28) 100.00
NOTE: Weights are from random effects analysis
.0017
1
590

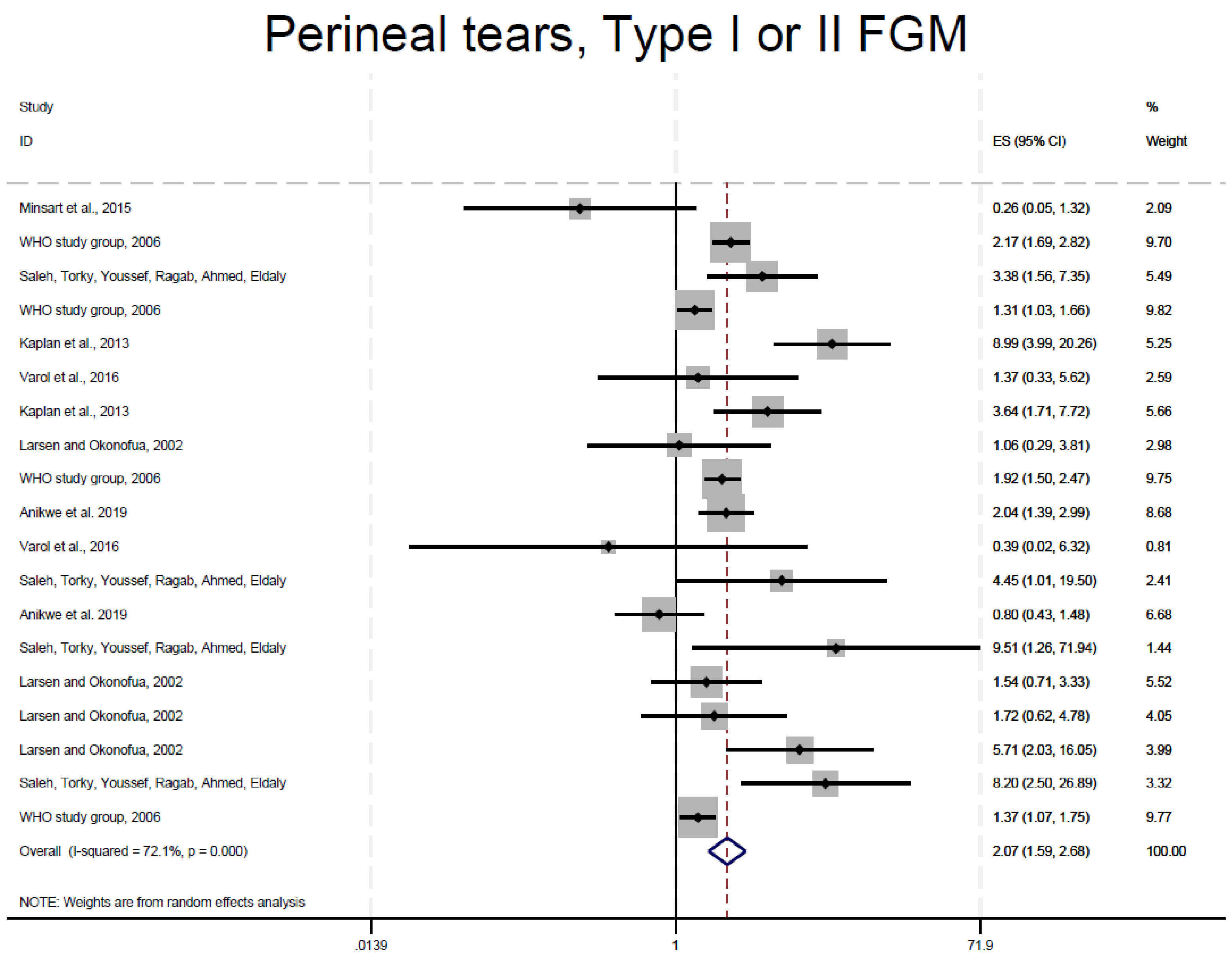
Perineal tears, Type I or II FGM
Study
ID
ES (95% CI)
%
Weight
Minsart et al., 2015
0.26 (0.05, 1.32)
2.09
WHO study group, 2006
2.17 (1.69, 2.82)
9.70
Saleh, Torky, Youssef, Ragab, Ahmed, Eldaly
3.38 (1.56, 7.35)
5.49
WHO study group, 2006
1.31 (1.03, 1.66)
9.82
Kaplan et al., 2013
8.99 (3.99, 20.26)
5.25
Varol et al., 2016
1.37 (0.33, 5.62)
2.59
Kaplan et al., 2013
3.64 (1.71, 7.72)
5.66
Larsen and Okonofua, 2002
1.06 (0.29, 3.81)
2.98
WHO study group, 2006
1.92 (1.50, 2.47)
9.75
Anikwe et al. 2019
2.04 (1.39, 2.99)
8.68
Varol et al., 2016
0.39 (0.02, 6.32)
0.81
Saleh, Torky, Youssef, Ragab, Ahmed, Eldaly
4.45 (1.01, 19.50)
2.41
Anikwe et al. 2019
0.80 (0.43, 1.48)
6.68
Saleh, Torky, Youssef, Ragab, Ahmed, Eldaly
9.51 (1.26, 71.94)
1.44
Larsen and Okonofua, 2002
1.54 (0.71, 3.33)
5.52
Larsen and Okonofua, 2002
1.72 (0.62, 4.78)
4.05
Larsen and Okonofua, 2002
5.71 (2.03, 16.05)
3.99
Saleh, Torky, Youssef, Ragab, Ahmed, Eldaly
8.20 (2.50, 26.89)
3.32
WHO study group, 2006
1.37 (1.07, 1.75)
9.77
Overall (I-squared = 72.1%, p = 0.000)
2.07 (1.59, 2.68)
100.00
NOTE: Weights are from random effects analysis
.0139
1
71.9

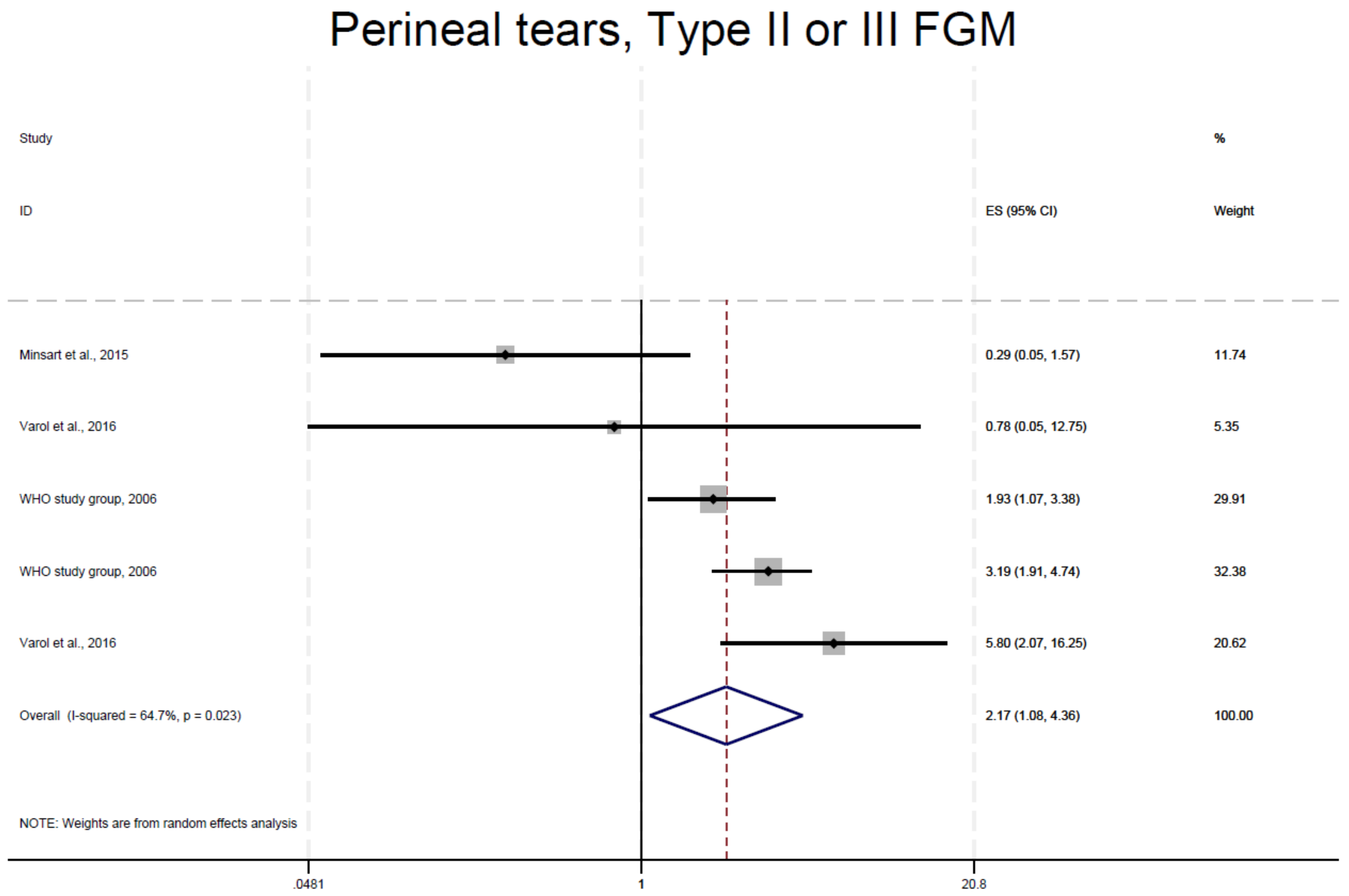
Perineal tears, Type II or III FGM
Study
ID
%
ES (95% CI)
Weight
Minsart et al., 2015
0.29 (0.05, 1.57)
11.74
Varol et al., 2016
0.78 (0.05, 12.75)
5.35
WHO study group, 2006
1.93 (1.07, 3.38)
29.91
WHO study group, 2006
3.19 (1.91, 4.74)
32.38
Varol et al., 2016
5.80 (2.07, 16.25)
20.62
Overall (I-squared = 64.7%, p = 0.023)
2.17 (1.08, 4.36)
100.00
NOTE: Weights are from random effects analysis
.0481
1
20.8

Episiotomy, Any FGM

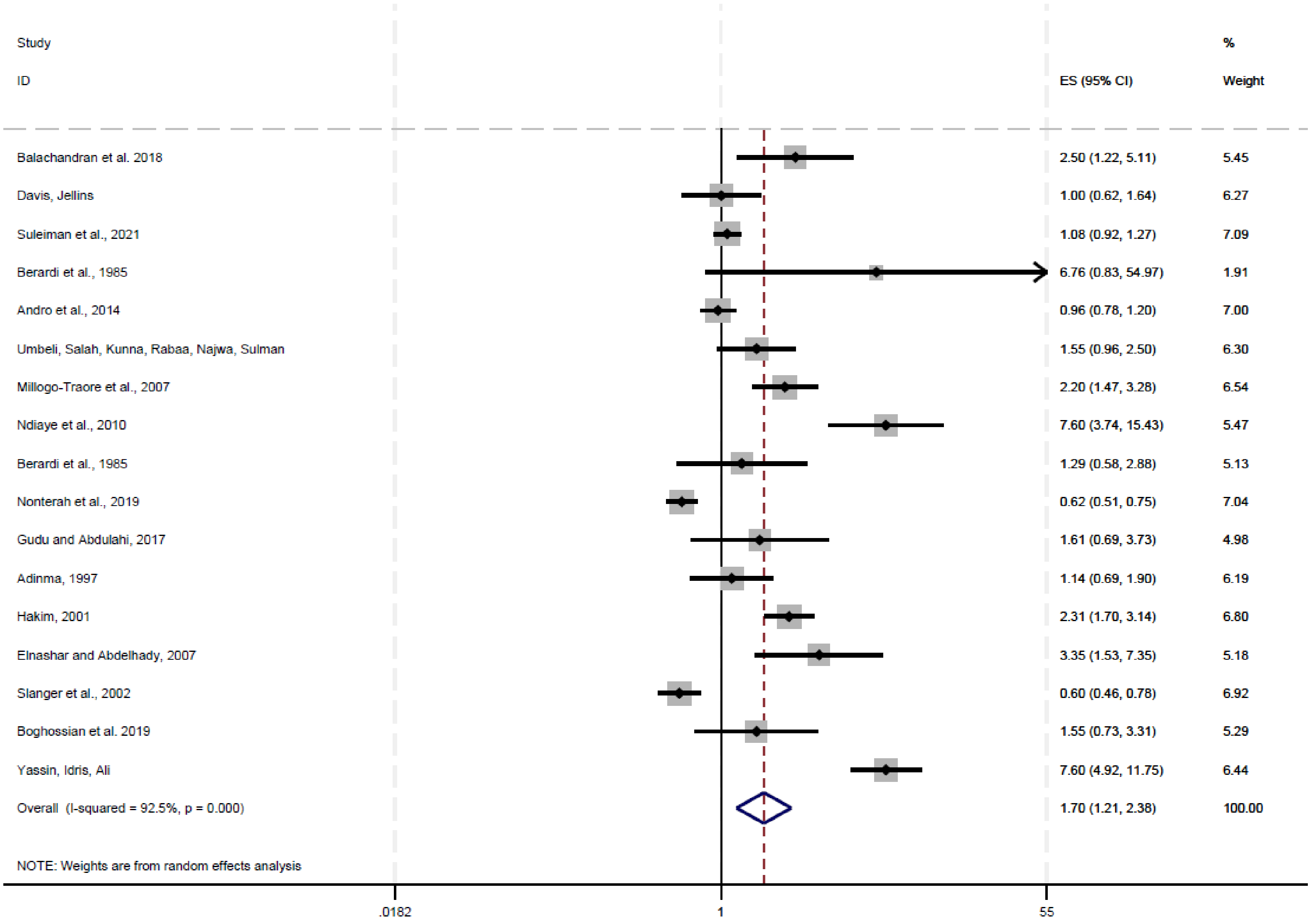

Episiotomy, Type I or II FGM

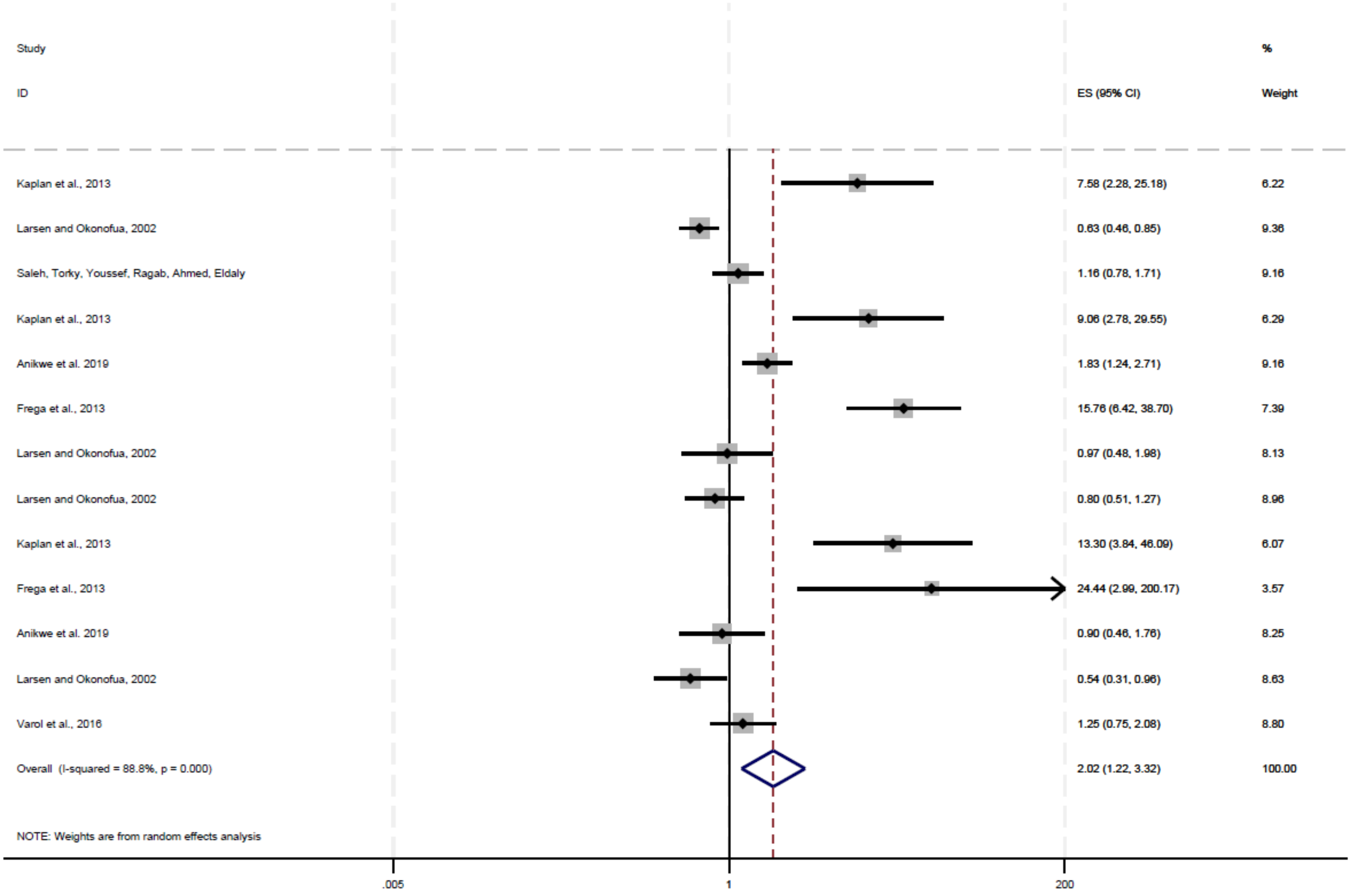

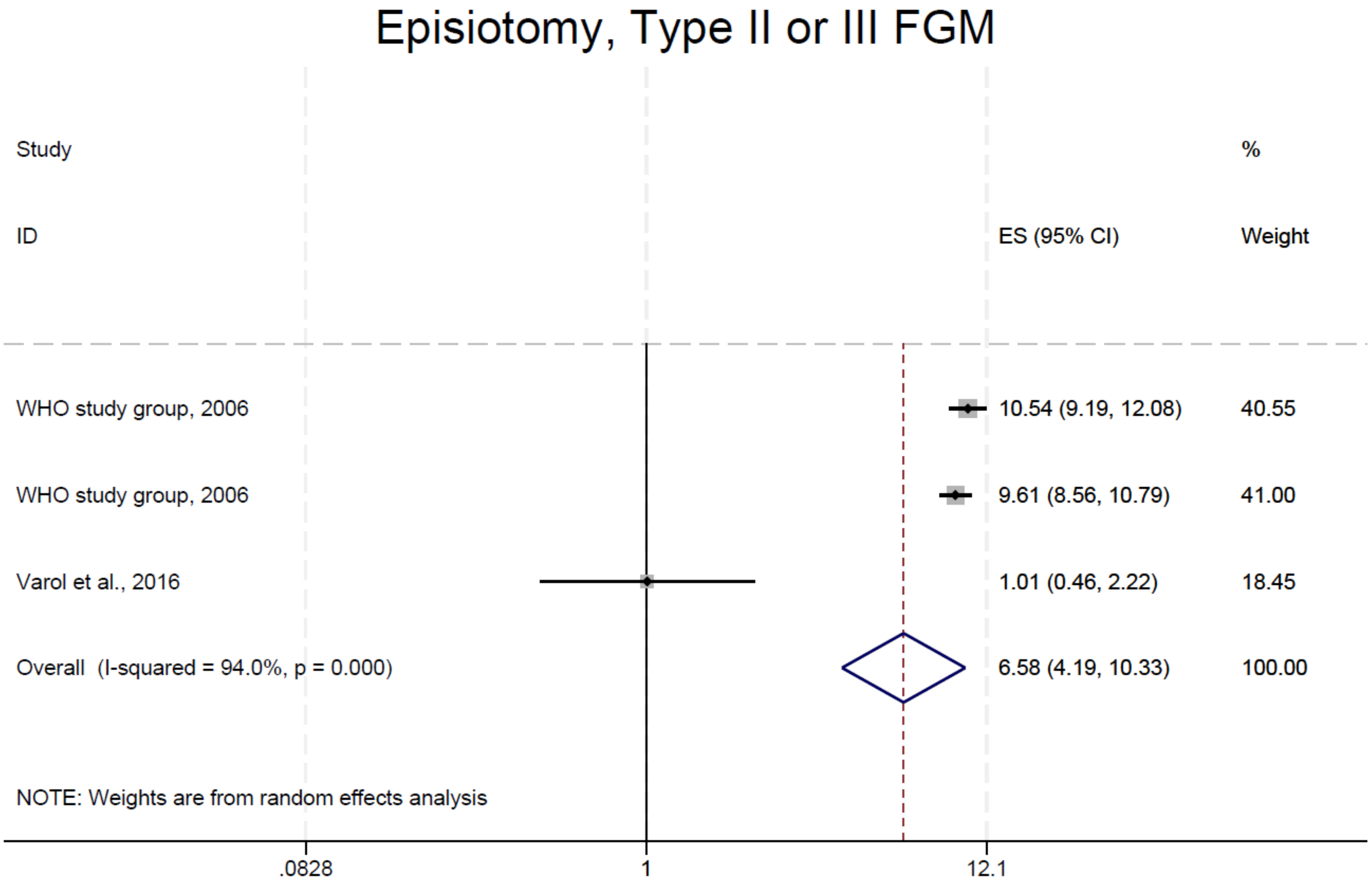

Episiotomy, Type II or III FGM
Study
ID
ES (95% CI)
%
Weight
WHO study group, 2006
10.54 (9.19, 12.08)
40.55
WHO study group, 2006
9.61 (8.56, 10.79)
41.00
Varol et al., 2016
1.01 (0.46, 2.22)
18.45
Overall (I-squared = 94.0%, p = 0.000)
6.58 (4.19, 10.33)
100.00
NOTE: Weights are from random effects analysis
.0828
1
12.1

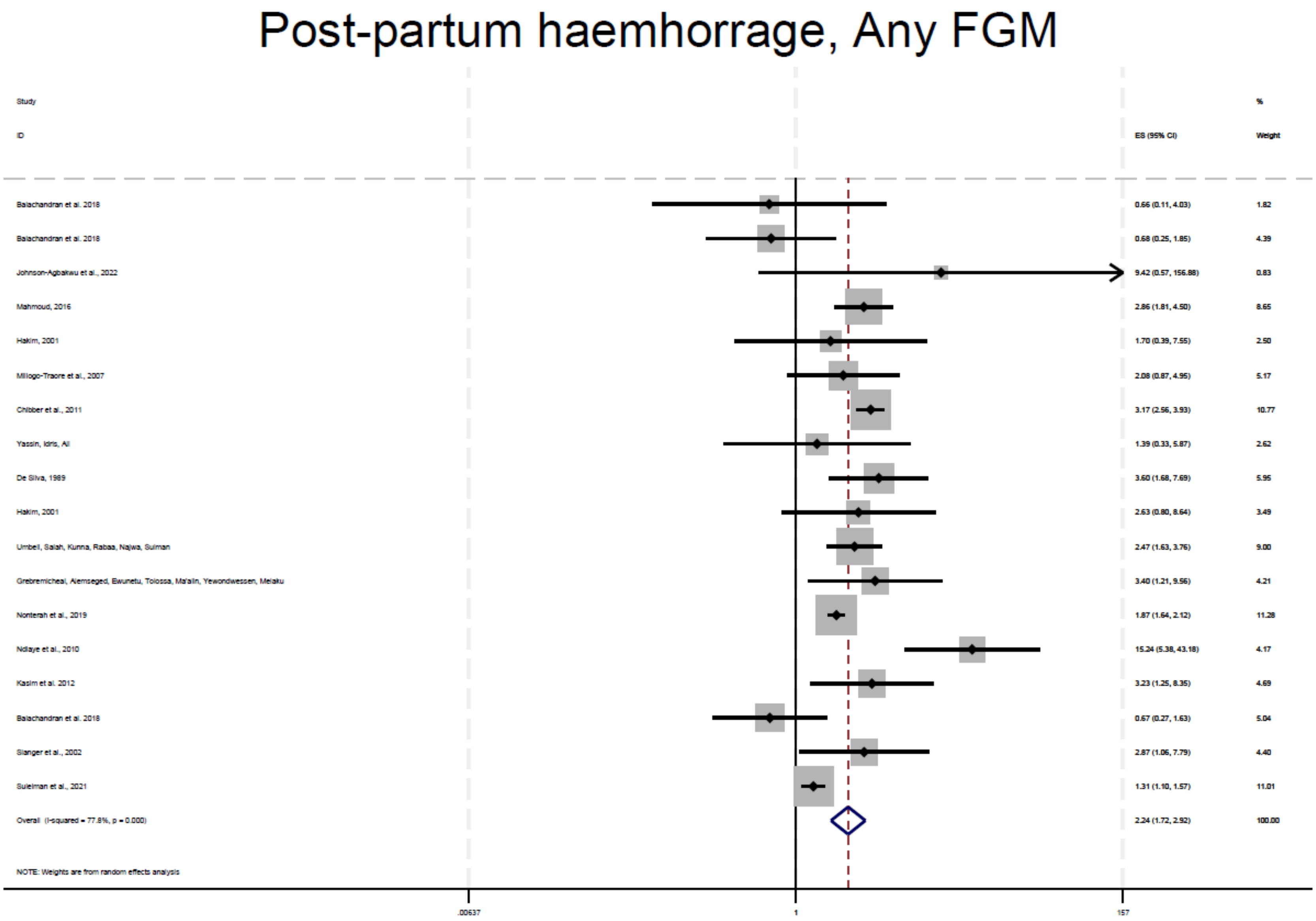
Post-partum haemhorrage, Any FGM
Study
ID
ES (95% CI)
%
Weight
Balachandran et al. 2018
0.66 (0.11, 4.03)
1.82
Balachandran et al. 2018
0.68 (0.25, 1.85)
4.39
Johnson-Agbakwu et al., 2022
9.42 (0.57, 156.88)
0.83
Mahmoud, 2016
2.86 (1.81, 4.50)
8.65
Hakim, 2001
1.70 (0.39, 7.55)
2.50
Millogo-Traore et al., 2007
2.08 (0.87, 4.95)
5.17
Chibber et al., 2011
3.17 (2.56, 3.93)
10.77
Yassin, Idris, Ali
1.39 (0.33, 5.87)
2.62
De Silva, 1989
3.60 (1.68, 7.69)
5.95
Hakim, 2001
2.63 (0.80, 8.64)
3.49
Umbeli, Salah, Kunna, Rabaa, Najwa, Sulman
2.47 (1.63, 3.76)
9.00
Grebremicheal, Alemseged, Ewunetu, Tolossa, Ma'alin, Yewondwessen, Melaku
3.40 (1.21, 9.56)
4.21
Nonterah et al., 2019
1.87 (1.64, 2.12)
11.28
Ndiaye et al., 2010
15.24 (5.38, 43.18)
4.17
Kasim et al. 2012
3.23 (1.25, 8.35)
4.69
Balachandran et al. 2018
0.67 (0.27, 1.63)
5.04
Slanger et al., 2002
2.87 (1.06, 7.79)
4.40
Suleiman et al., 2021
1.31 (1.10, 1.57)
11.01
Overall (I-squared = 77.8%, p = 0.000)
2.24 (1.72, 2.92)
100.00
NOTE: Weights are from random effects analysis
.00637
1
157

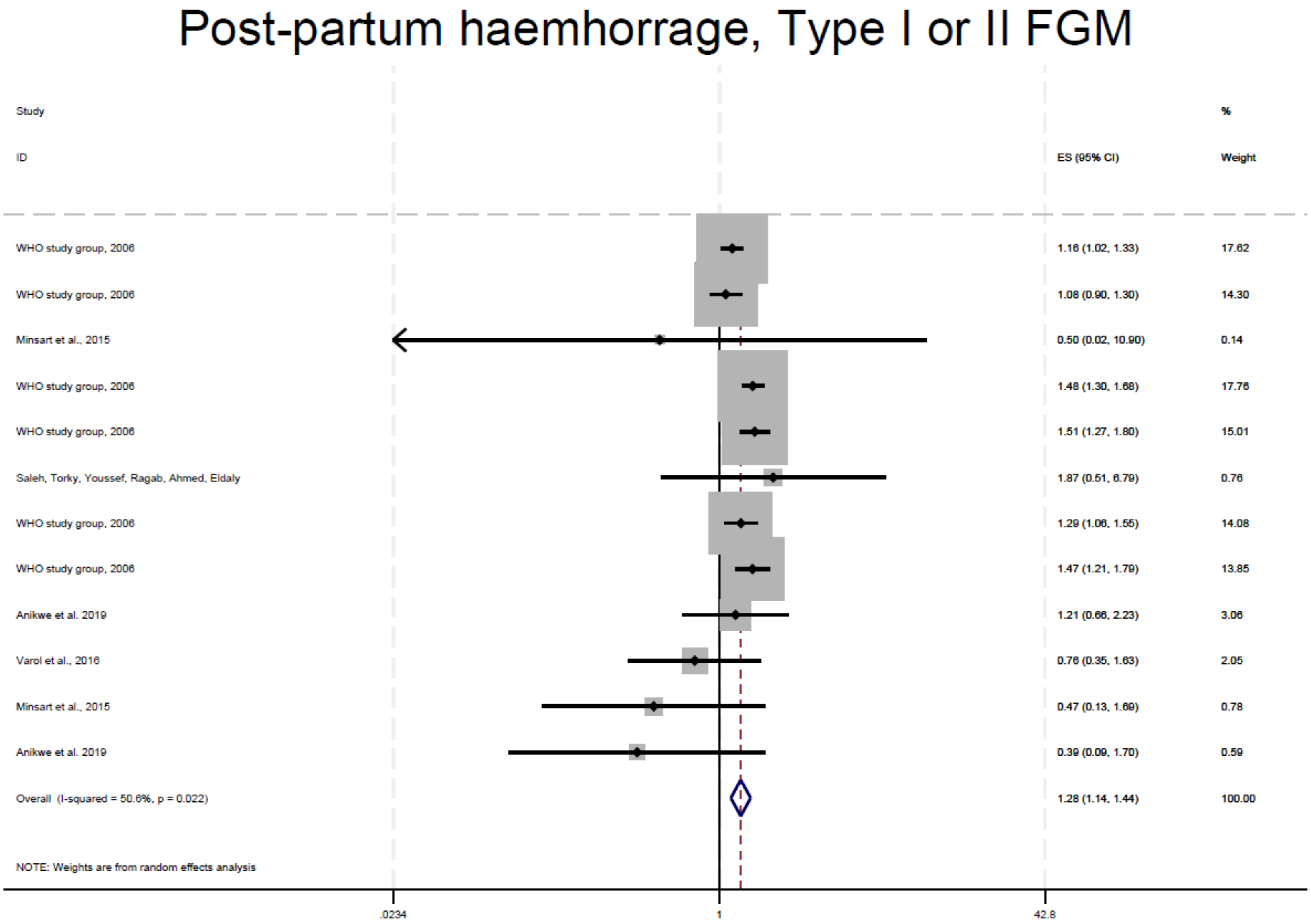
Post-partum haemhorrage, Type I or II FGM
Study
ID
ES (95% CI)
%
Weight
WHO study group, 2006
1.16 (1.02, 1.33)
17.62
WHO study group, 2006
1.08 (0.90, 1.30)
14.30
Minsart et al., 2015
0.50 (0.02, 10.90)
0.14
WHO study group, 2006
1.48 (1.30, 1.68)
17.76
WHO study group, 2006
1.51 (1.27, 1.80)
15.01
Saleh, Torky, Youssef, Ragab, Ahmed, Eldaly
1.87 (0.51, 6.79)
0.76
WHO study group, 2006
1.29 (1.06, 1.55)
14.08
WHO study group, 2006
1.47 (1.21, 1.79)
13.85
Anikwe et al. 2019
1.21 (0.66, 2.23)
3.06
Varol et al., 2016
0.76 (0.35, 1.63)
2.05
Minsart et al., 2015
0.47 (0.13, 1.69)
0.78
Anikwe et al. 2019
0.39 (0.09, 1.70)
0.59
Overall (I-squared = 50.6%, p = 0.022)
1.28 (1.14, 1.44)
100.00
NOTE: Weights are from random effects analysis
.0234
1
42.8

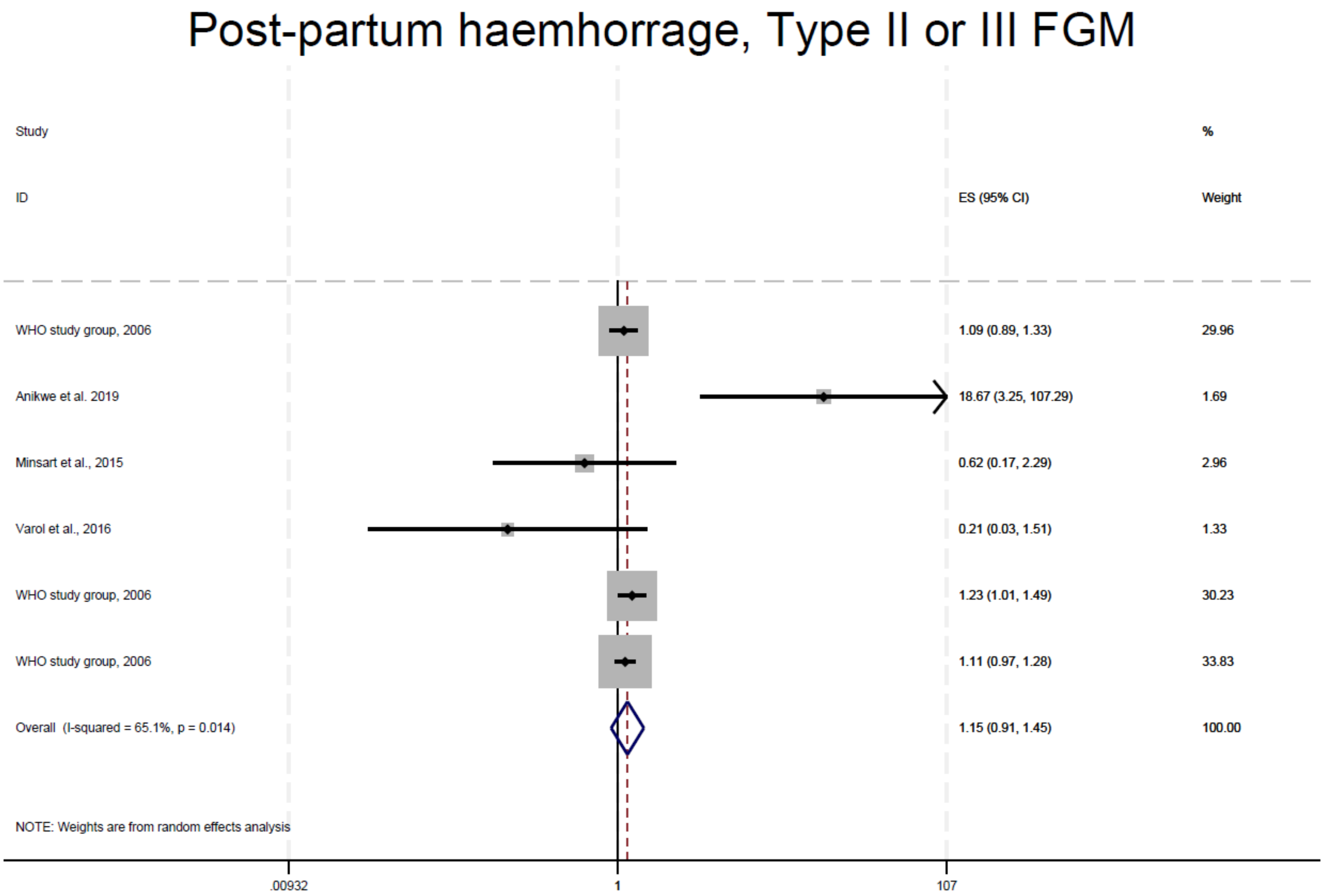
Post-partum haemhorrage, Type II or III FGM
Study
ID
ES (95% CI)
%
Weight
WHO study group, 2006
1.09 (0.89, 1.33)
29.96
Anikwe et al. 2019
18.67 (3.25, 107.29)
1.69
Minsart et al., 2015
0.62 (0.17, 2.29)
2.96
Varol et al., 2016
0.21 (0.03, 1.51)
1.33
WHO study group, 2006
1.23 (1.01, 1.49)
30.23
WHO study group, 2006
1.11 (0.97, 1.28)
33.83
Overall (I-squared = 65.1%, p = 0.014)
1.15 (0.91, 1.45)
100.00
NOTE: Weights are from random effects analysis
.00932
1
107

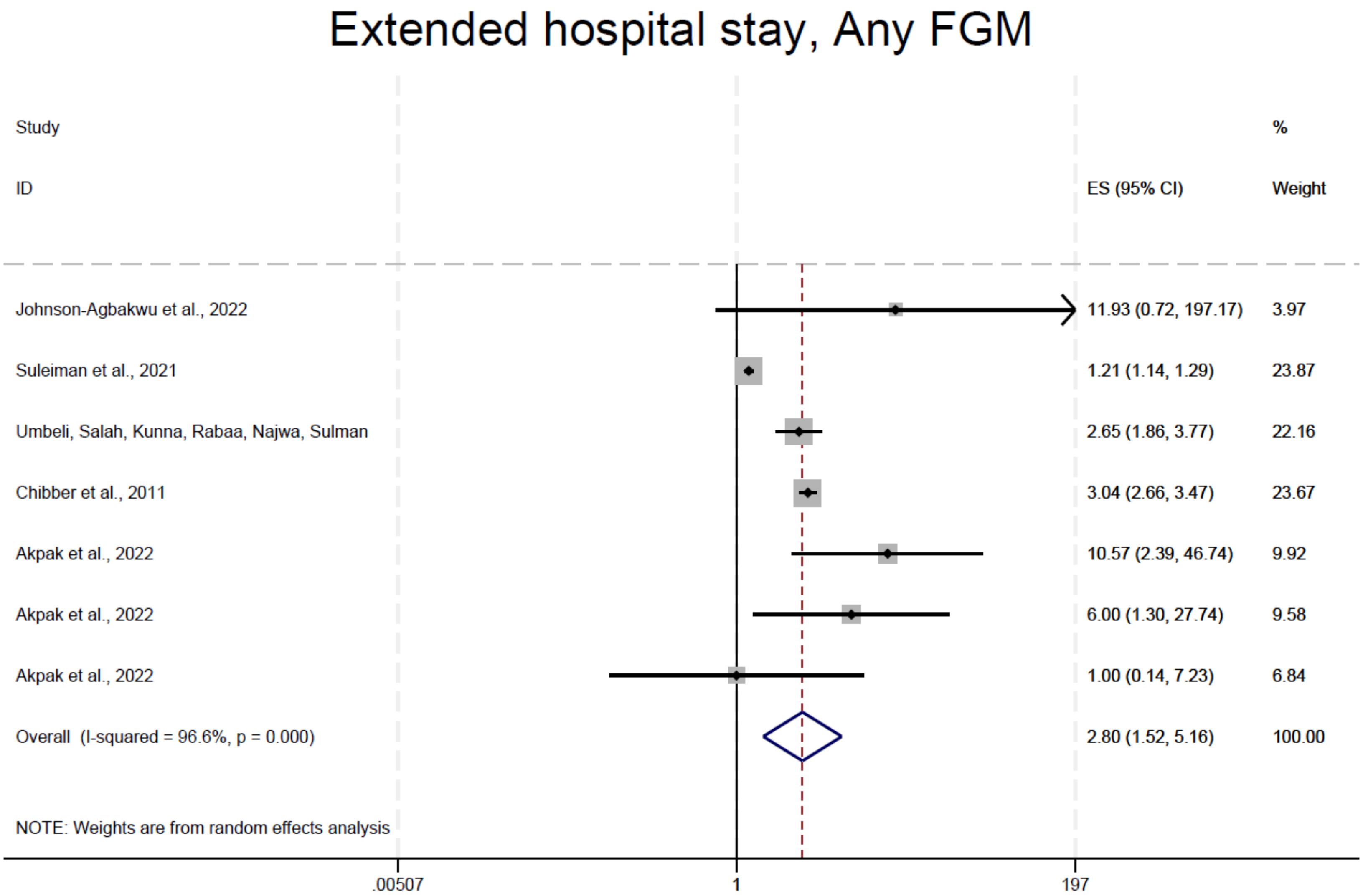

Extended hospital stay, Any FGM
Study
ID
ES (95% CI)
%
Weight
Johnson-Agbakwu et al., 2022
11.93 (0.72, 197.17)
3.97
Suleiman et al., 2021
1.21 (1.14, 1.29)
23.87
Umbeli, Salah, Kunna, Rabaa, Najwa, Sulman
2.65 (1.86, 3.77)
22.16
Chibber et al., 2011
3.04 (2.66, 3.47)
23.67
Akpak et al., 2022
10.57 (2.39, 46.74)
9.92
Akpak et al., 2022
6.00 (1.30, 27.74)
9.58
Akpak et al., 2022
1.00 (0.14, 7.23)
6.84
Overall (I-squared = 96.6%, p = 0.000)
2.80 (1.52, 5.16)
100.00
NOTE: Weights are from random effects analysis
.00507
1
197

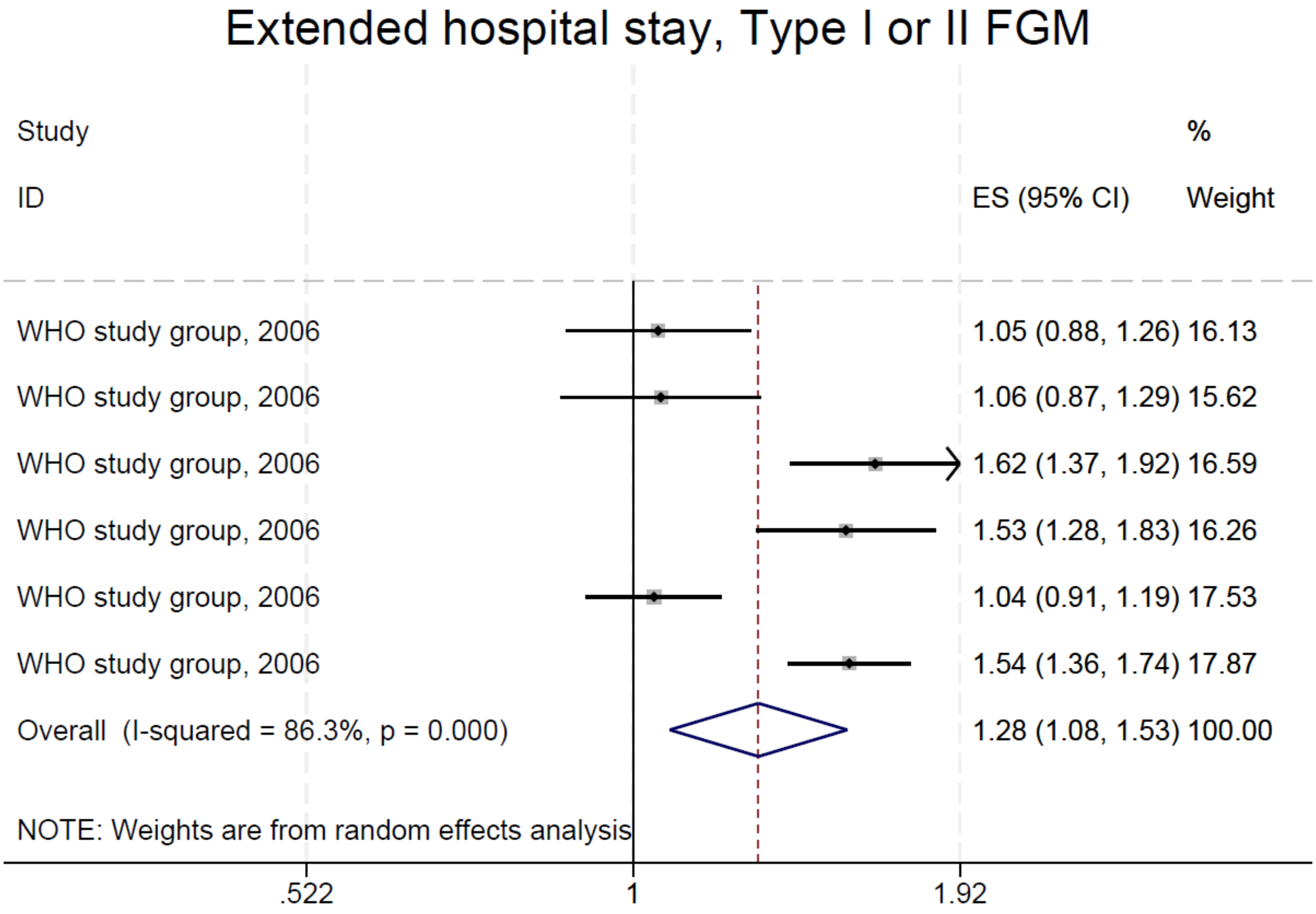

Extended hospital stay, Type I or II FGM
Study
ID
ES (95% CI)
%
Weight
WHO study group, 2006
1.05 (0.88, 1.26) 16.13
WHO study group, 2006
1.06 (0.87, 1.29) 15.62
WHO study group, 2006
1.62 (1.37, 1.92) 16.59
WHO study group, 2006
1.53 (1.28, 1.83) 16.26
WHO study group, 2006
1.04 (0.91, 1.19) 17.53
WHO study group, 2006
1.54 (1.36, 1.74) 17.87
Overall (I-squared = 86.3%, p = 0.000)
1.28 (1.08, 1.53) 100.00
NOTE: Weights are from random effects analysis
.522
1
1.92

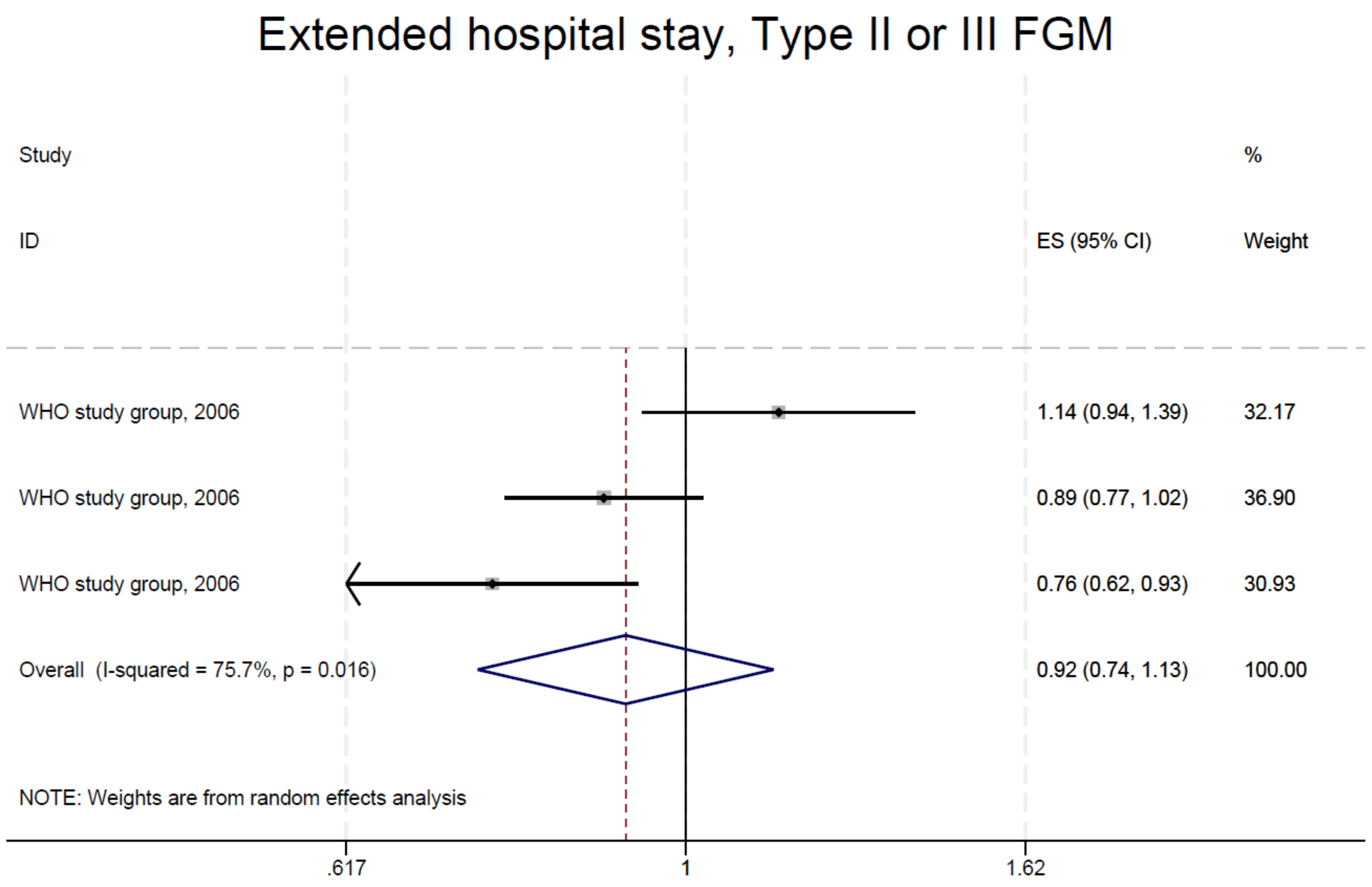
Extended hospital stay, Type II or III FGM
Study
ID
ES (95% CI)
%
Weight
WHO study group, 2006
1.14 (0.94, 1.39)
32.17
WHO study group, 2006
0.89 (0.77, 1.02)
36.90
WHO study group, 2006
0.76 (0.62, 0.93)
30.93
Overall (I-squared = 75.7%, p = 0.016)
0.92 (0.74, 1.13)
100.00
NOTE: Weights are from random effects analysis
.617
1
1.62

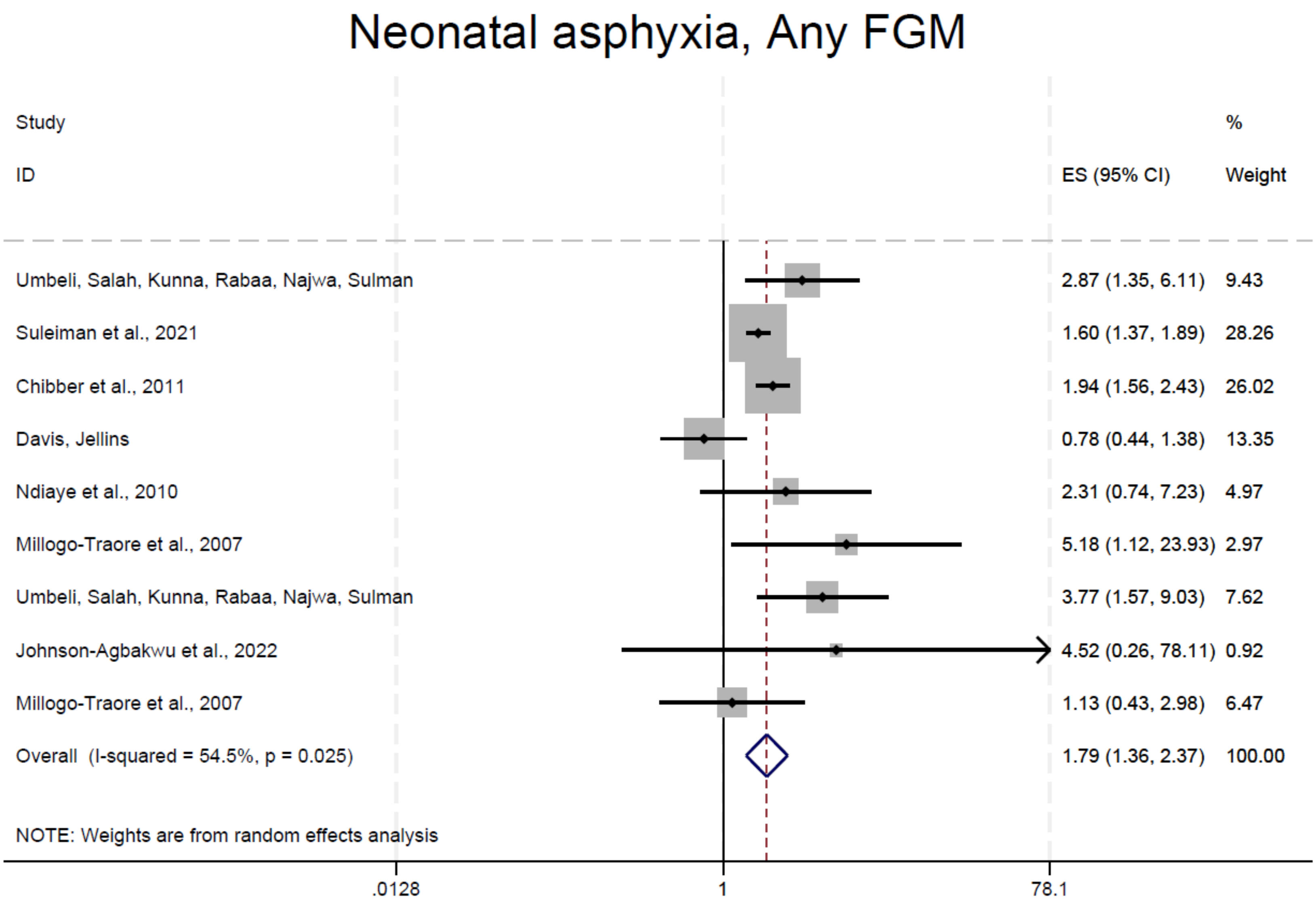
Neonatal asphyxia, Any FGM
Study
ID
ES (95% CI)
%
Weight
Umbeli, Salah, Kunna, Rabaa, Najwa, Sulman 2.87 (1.35, 6.11) 9.43
Suleiman et al., 2021 1.60 (1.37, 1.89) 28.26
Chibber et al., 2011 1.94 (1.56, 2.43) 26.02
Davis, Jellins 0.78 (0.44, 1.38) 13.35
Ndiaye et al., 2010 2.31 (0.74, 7.23) 4.97
Millogo-Traore et al., 2007 5.18 (1.12, 23.93) 2.97
Umbeli, Salah, Kunna, Rabaa, Najwa, Sulman 3.77 (1.57, 9.03) 7.62
Johnson-Agbakwu et al., 2022 4.52 (0.26, 78.11) 0.92
Millogo-Traore et al., 2007 1.13 (0.43, 2.98) 6.47
Overall (I-squared = 54.5%, p = 0.025) 1.79 (1.36, 2.37) 100.00
NOTE: Weights are from random effects analysis
.0128
1
78.1

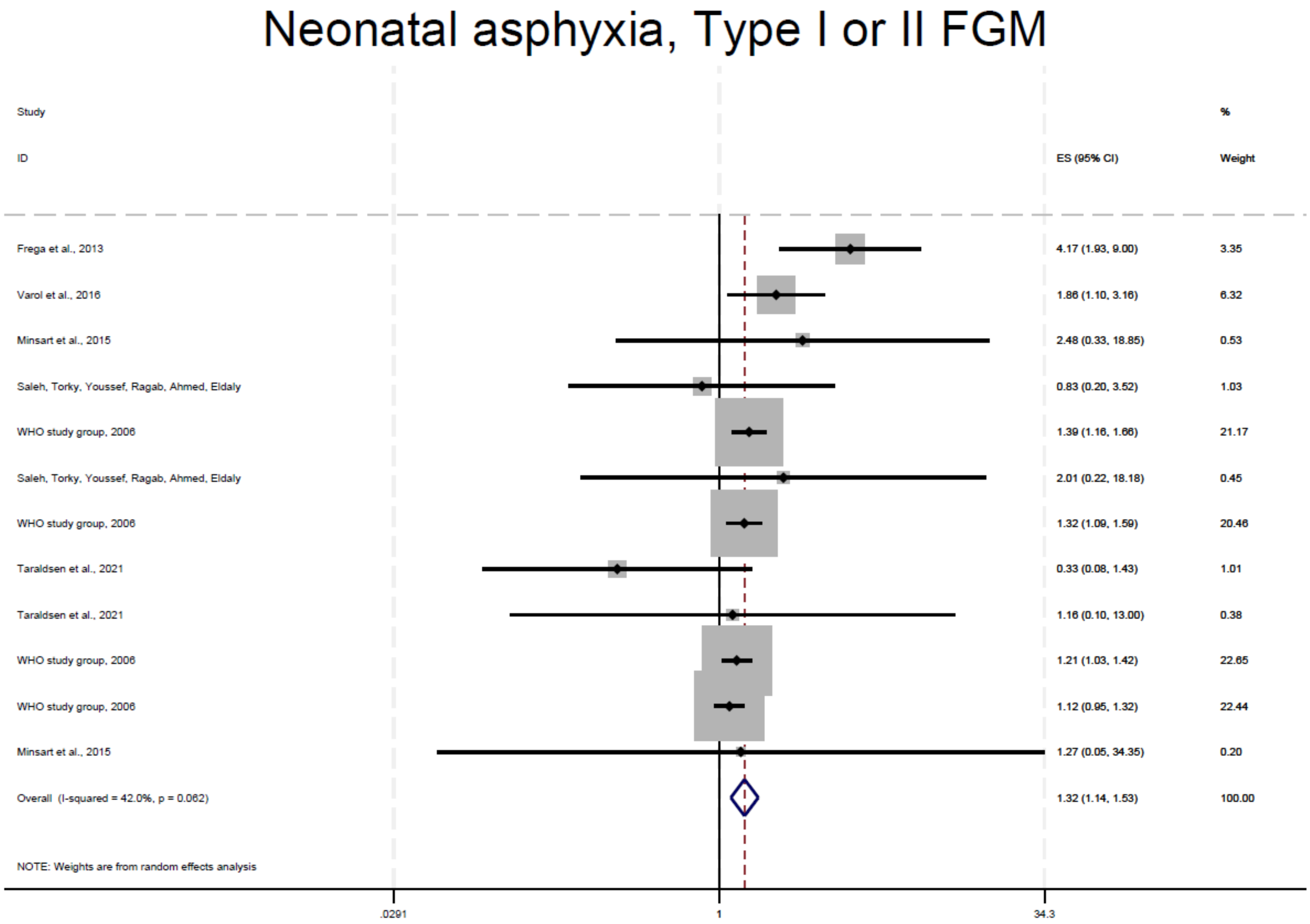
Neonatal asphyxia, Type I or II FGM
Study
ID
ES (95% CI)
%
Weight
Frega et al., 2013
4.17 (1.93, 9.00)
3.35
Varol et al., 2016
1.86 (1.10, 3.16)
6.32
Minsart et al., 2015
2.48 (0.33, 18.85)
0.53
Saleh, Torky, Youssef, Ragab, Ahmed, Eldaly
0.83 (0.20, 3.52)
1.03
WHO study group, 2006
1.39 (1.16, 1.66)
21.17
Saleh, Torky, Youssef, Ragab, Ahmed, Eldaly
2.01 (0.22, 18.18)
0.45
WHO study group, 2006
1.32 (1.09, 1.59)
20.46
Taraldsen et al., 2021
0.33 (0.08, 1.43)
1.01
Taraldsen et al., 2021
1.16 (0.10, 13.00)
0.38
WHO study group, 2006
1.21 (1.03, 1.42)
22.65
WHO study group, 2006
1.12 (0.95, 1.32)
22.44
Minsart et al., 2015
1.27 (0.05, 34.35)
0.20
Overall (I-squared = 42.0%, p = 0.062)
1.32 (1.14, 1.53)
100.00
NOTE: Weights are from random effects analysis
.0291
1
34.3

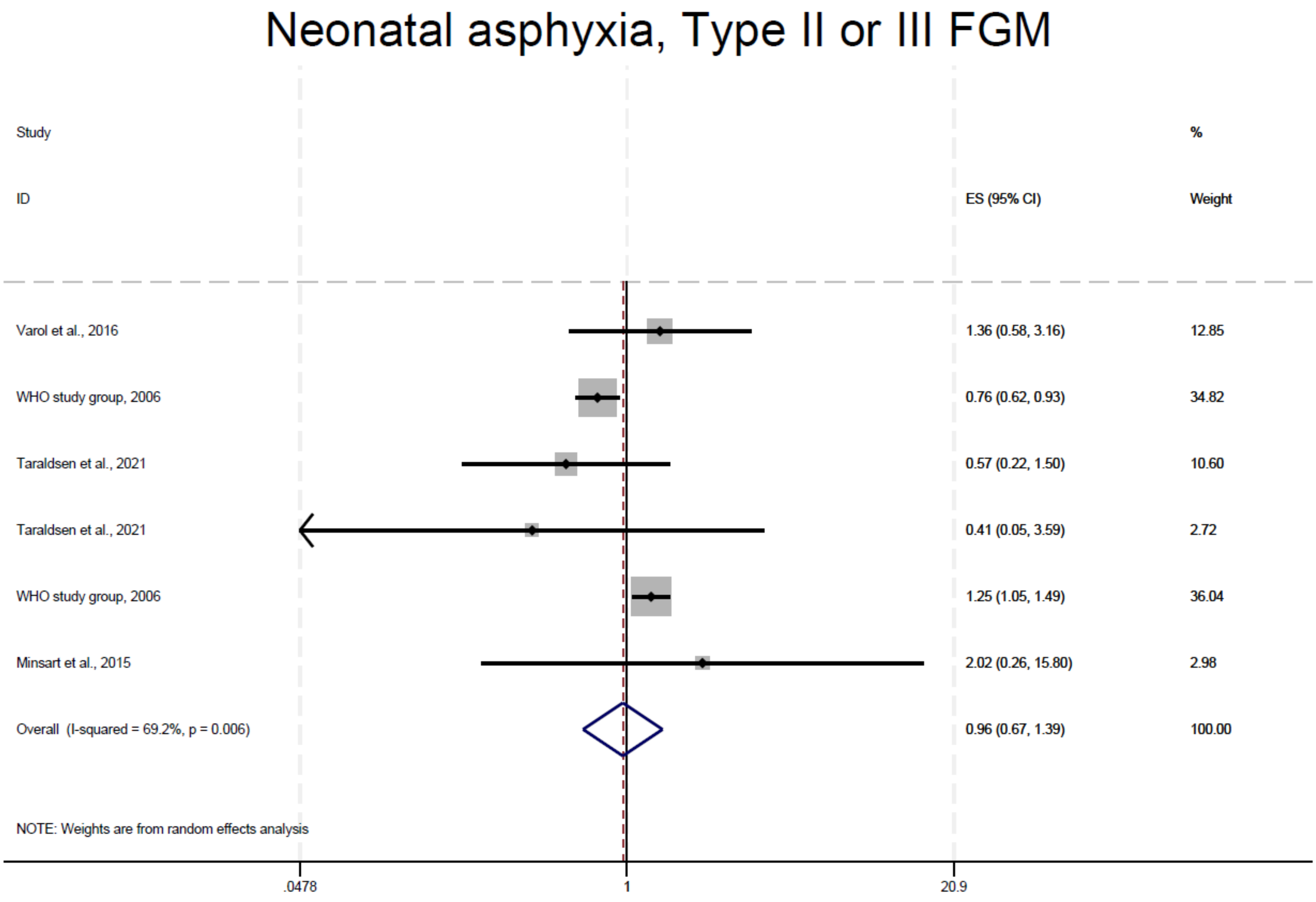
Neonatal asphyxia, Type II or III FGM
Study
ID
ES (95% CI)
%
Weight
Varol et al., 2016
1.36 (0.58, 3.16)
12.85
WHO study group, 2006
0.76 (0.62, 0.93)
34.82
Taraldsen et al., 2021
0.57 (0.22, 1.50)
10.60
Taraldsen et al., 2021
0.41 (0.05, 3.59)
2.72
WHO study group, 2006
1.25 (1.05, 1.49)
36.04
Minsart et al., 2015
2.02 (0.26, 15.80)
2.98
Overall (I-squared = 69.2%, p = 0.006)
0.96 (0.67, 1.39)
100.00
NOTE: Weights are from random effects analysis
.0478
1
20.9

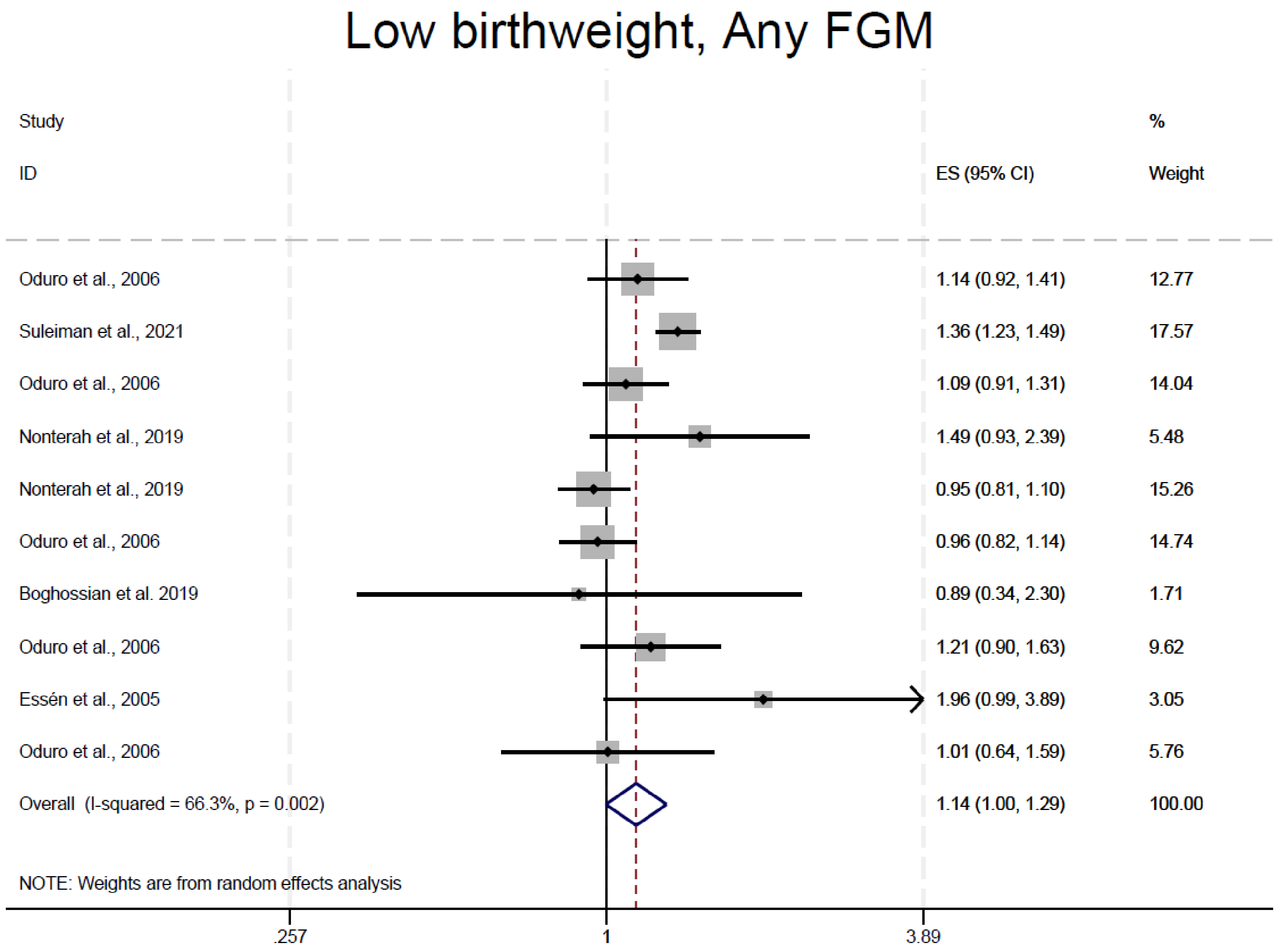
Low birthweight, Any FGM
Study
ID
ES (95% CI)
%
Weight
Oduro et al., 2006
1.14 (0.92, 1.41)
12.77
Suleiman et al., 2021
1.36 (1.23, 1.49)
17.57
Oduro et al., 2006
1.09 (0.91, 1.31)
14.04
Nonterah et al., 2019
1.49 (0.93, 2.39)
5.48
Nonterah et al., 2019
0.95 (0.81, 1.10)
15.26
Oduro et al., 2006
0.96 (0.82, 1.14)
14.74
Boghossian et al. 2019
0.89 (0.34, 2.30)
1.71
Oduro et al., 2006
1.21 (0.90, 1.63)
9.62
Essén et al., 2005
1.96 (0.99, 3.89)
3.05
Oduro et al., 2006
1.01 (0.64, 1.59)
5.76
Overall (I-squared = 66.3%, p = 0.002)
1.14 (1.00, 1.29)
100.00
NOTE: Weights are from random effects analysis
.257
1
3.89

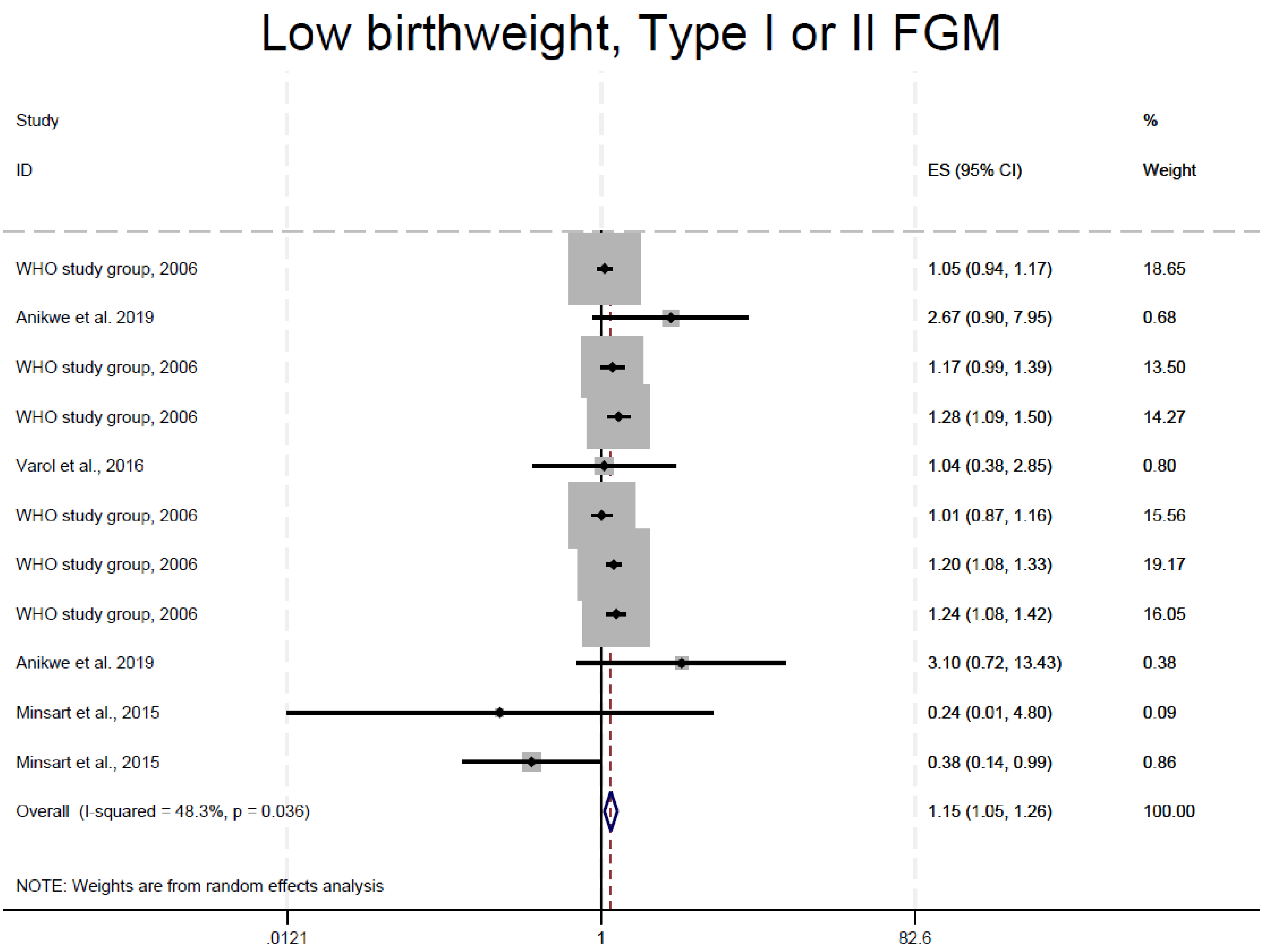
Low birthweight, Type I or II FGM
Study
ID
ES (95% CI)
%
Weight
WHO study group, 2006 1.05 (0.94, 1.17) 18.65
Anikwe et al. 2019 2.67 (0.90, 7.95) 0.68
WHO study group, 2006 1.17 (0.99, 1.39) 13.50
WHO study group, 2006 1.28 (1.09, 1.50) 14.27
Varol et al., 2016 1.04 (0.38, 2.85) 0.80
WHO study group, 2006 1.01 (0.87, 1.16) 15.56
WHO study group, 2006 1.20 (1.08, 1.33) 19.17
WHO study group, 2006 1.24 (1.08, 1.42) 16.05
Anikwe et al. 2019 3.10 (0.72, 13.43) 0.38
Minsart et al., 2015 0.24 (0.01, 4.80) 0.09
Minsart et al., 2015 0.38 (0.14, 0.99) 0.86
Overall (I-squared = 48.3%, p = 0.036) 1.15 (1.05, 1.26) 100.00
NOTE: Weights are from random effects analysis
.0121
1
82.6

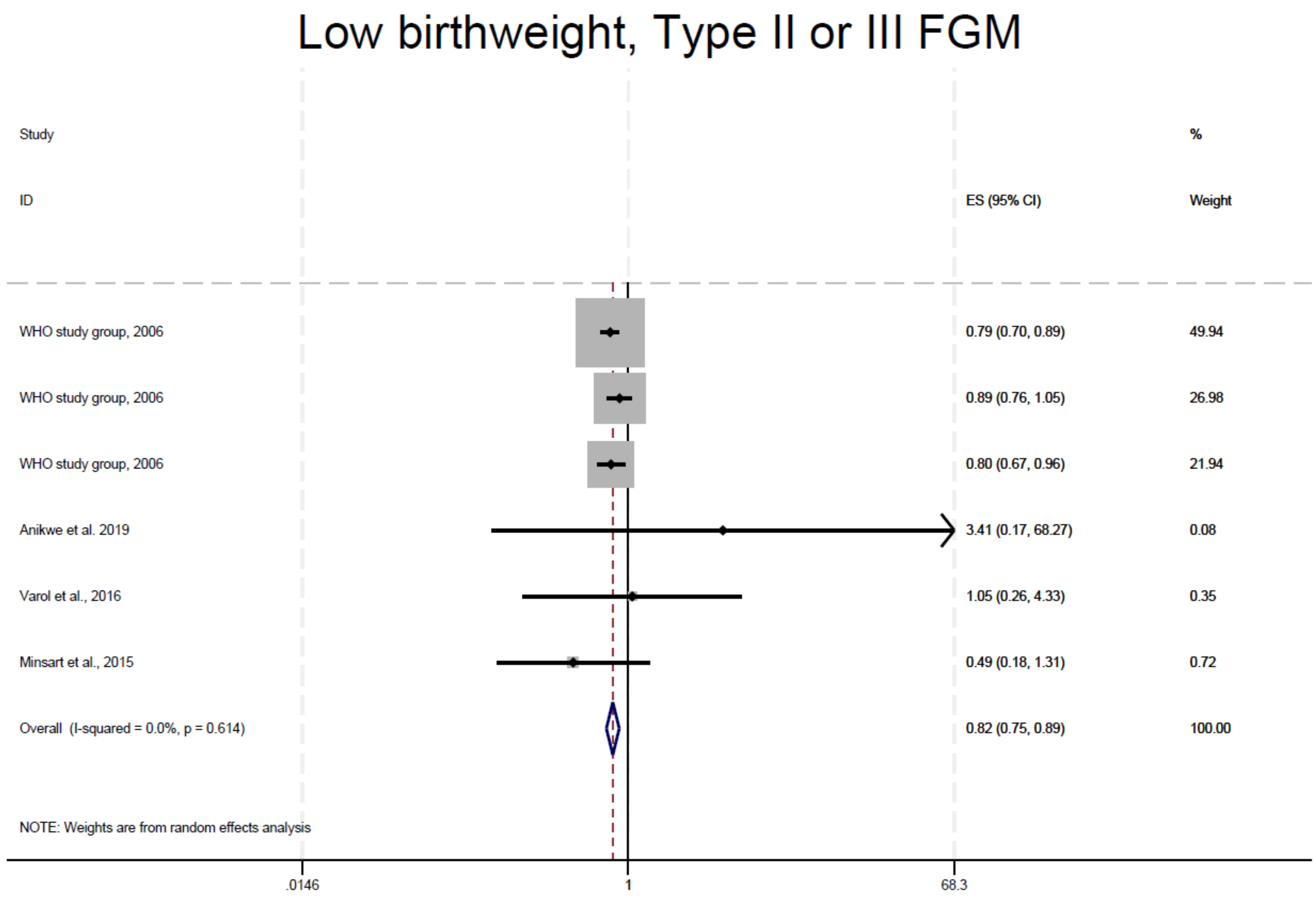
Low birthweight, Type II or III FGM
Study
ID
ES (95% CI)
%
Weight
WHO study group, 2006
0.79 (0.70, 0.89)
49.94
WHO study group, 2006
0.89 (0.76, 1.05)
26.98
WHO study group, 2006
0.80 (0.67, 0.96)
21.94
Anikwe et al. 2019
3.41 (0.17, 68.27)
0.08
Varol et al., 2016
1.05 (0.26, 4.33)
0.35
Minsart et al., 2015
0.49 (0.18, 1.31)
0.72
Overall (I-squared = 0.0%, p = 0.614)
0.82 (0.75, 0.89)
100.00
NOTE: Weights are from random effects analysis
.0146
1
68.3

Instrumental delivery, Any FGM

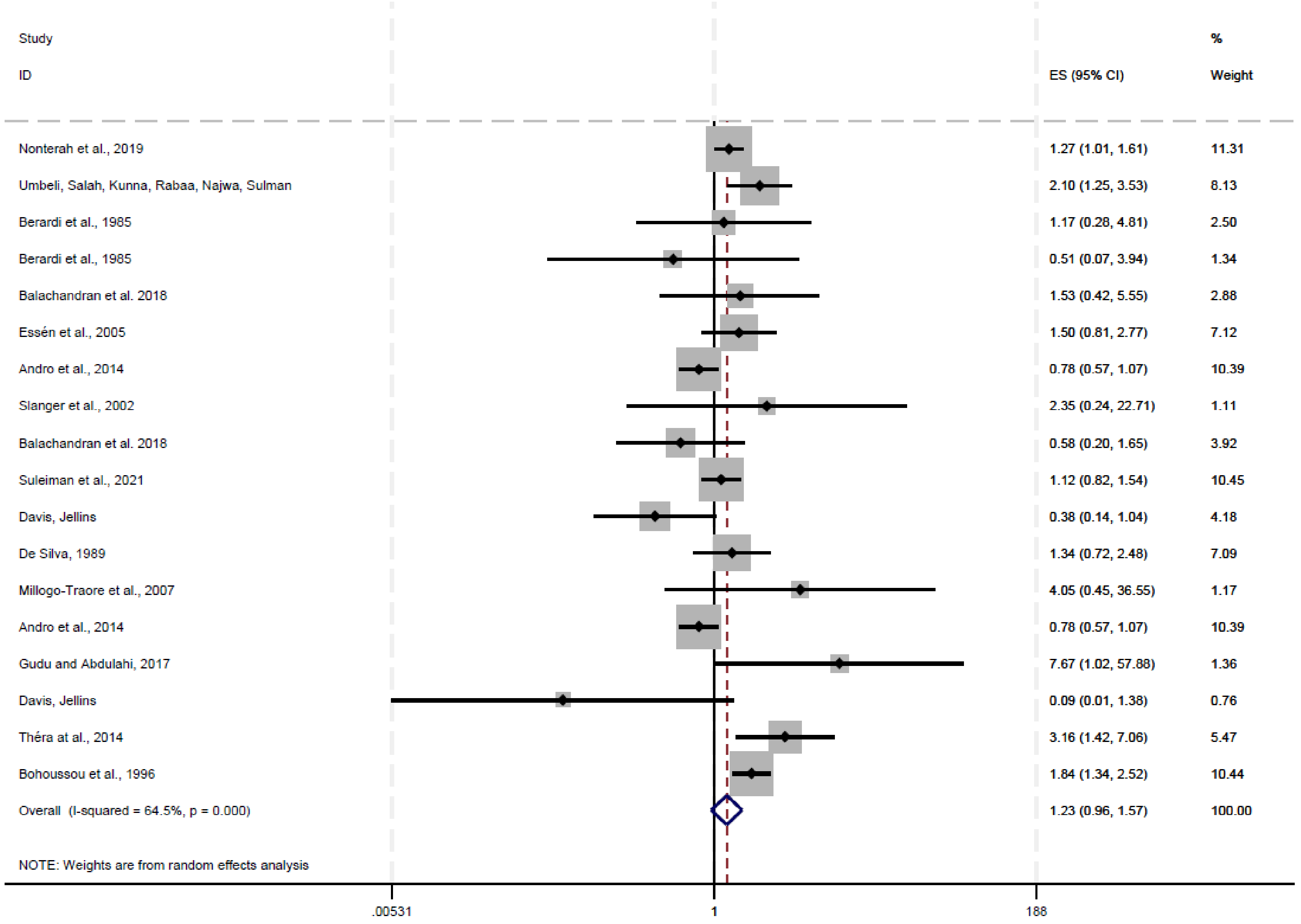

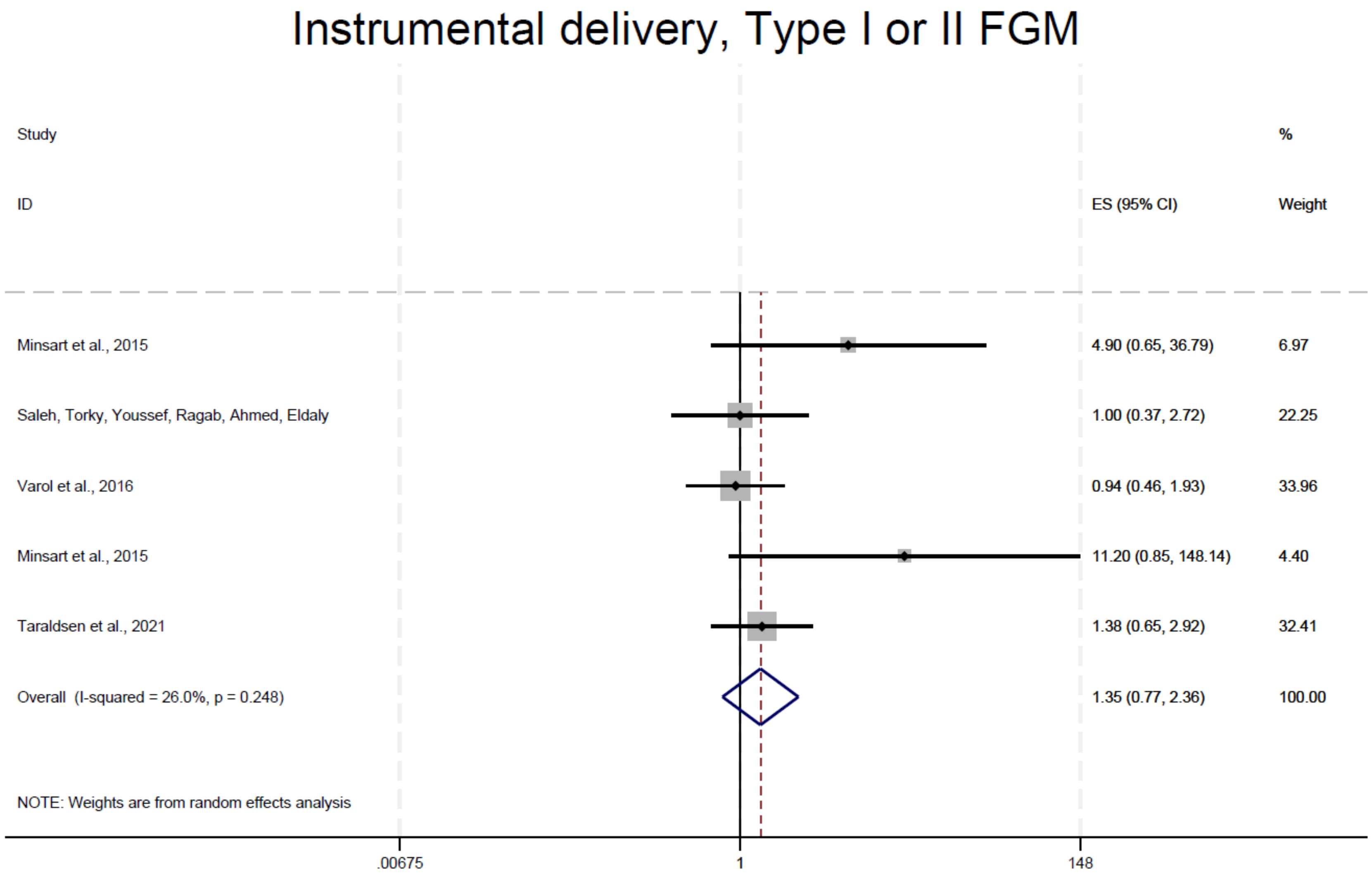
Instrumental delivery, Type I or II FGM
Study
ID
ES (95% CI)
%
Weight
Minsart et al., 2015
4.90 (0.65, 36.79)
6.97
Saleh, Torky, Youssef, Ragab, Ahmed, Eldaly
1.00 (0.37, 2.72)
22.25
Varol et al., 2016
0.94 (0.46, 1.93)
33.96
Minsart et al., 2015
11.20 (0.85, 148.14)
4.40
Taraldsen et al., 2021
1.38 (0.65, 2.92)
32.41
Overall (I-squared = 26.0%, p = 0.248)
1.35 (0.77, 2.36)
100.00
NOTE: Weights are from random effects analysis
.00675
1
148

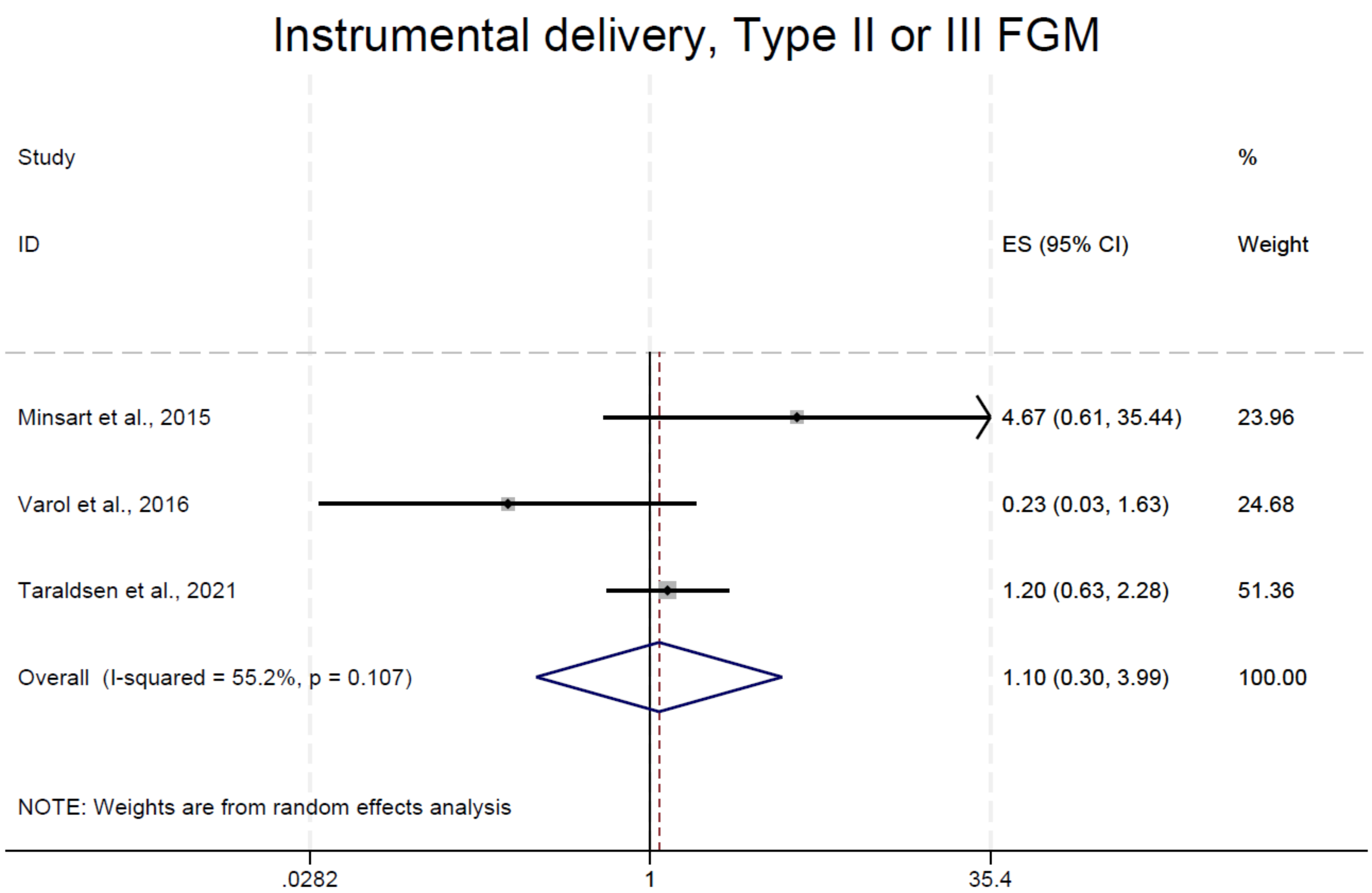

Instrumental delivery, Type II or III FGM
Study
ID
%
ES (95% CI)
Weight
Minsart et al., 2015
4.67 (0.61, 35.44)
23.96
Varol et al., 2016
0.23 (0.03, 1.63)
24.68
Taraldsen et al., 2021
1.20 (0.63, 2.28)
51.36
Overall (I-squared = 55.2%, p = 0.107)
1.10 (0.30, 3.99)
100.00
NOTE: Weights are from random effects analysis
.0282
1
35.4

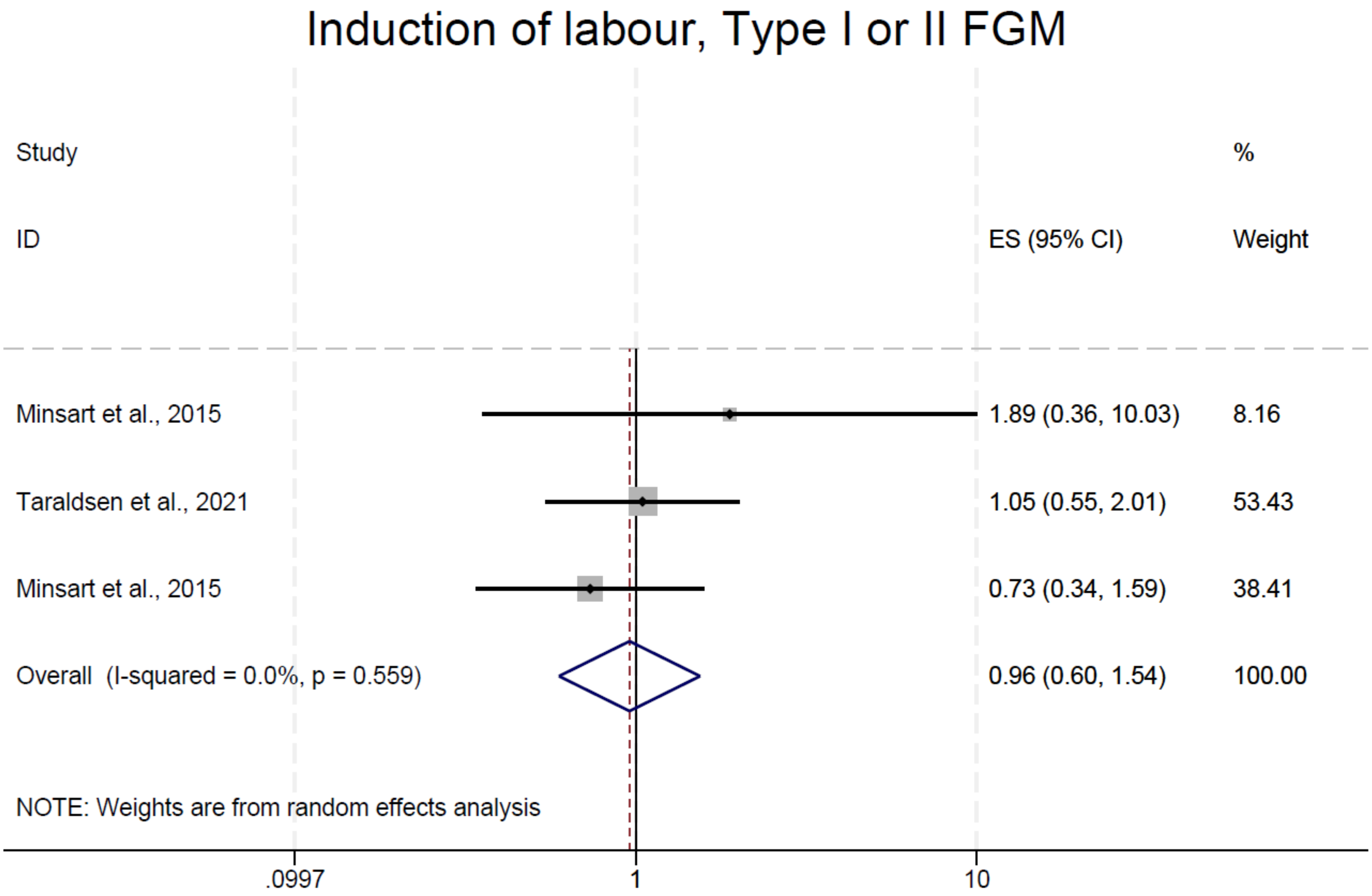
Induction of labour, Type I or II FGM
Study
ID
ES (95% CI)
%
Weight
Minsart et al., 2015
1.89 (0.36, 10.03)
8.16
Taraldsen et al., 2021
1.05 (0.55, 2.01)
53.43
Minsart et al., 2015
0.73 (0.34, 1.59)
38.41
Overall (I-squared = 0.0%, p = 0.559)
0.96 (0.60, 1.54)
100.00
NOTE: Weights are from random effects analysis
.0997
1
10

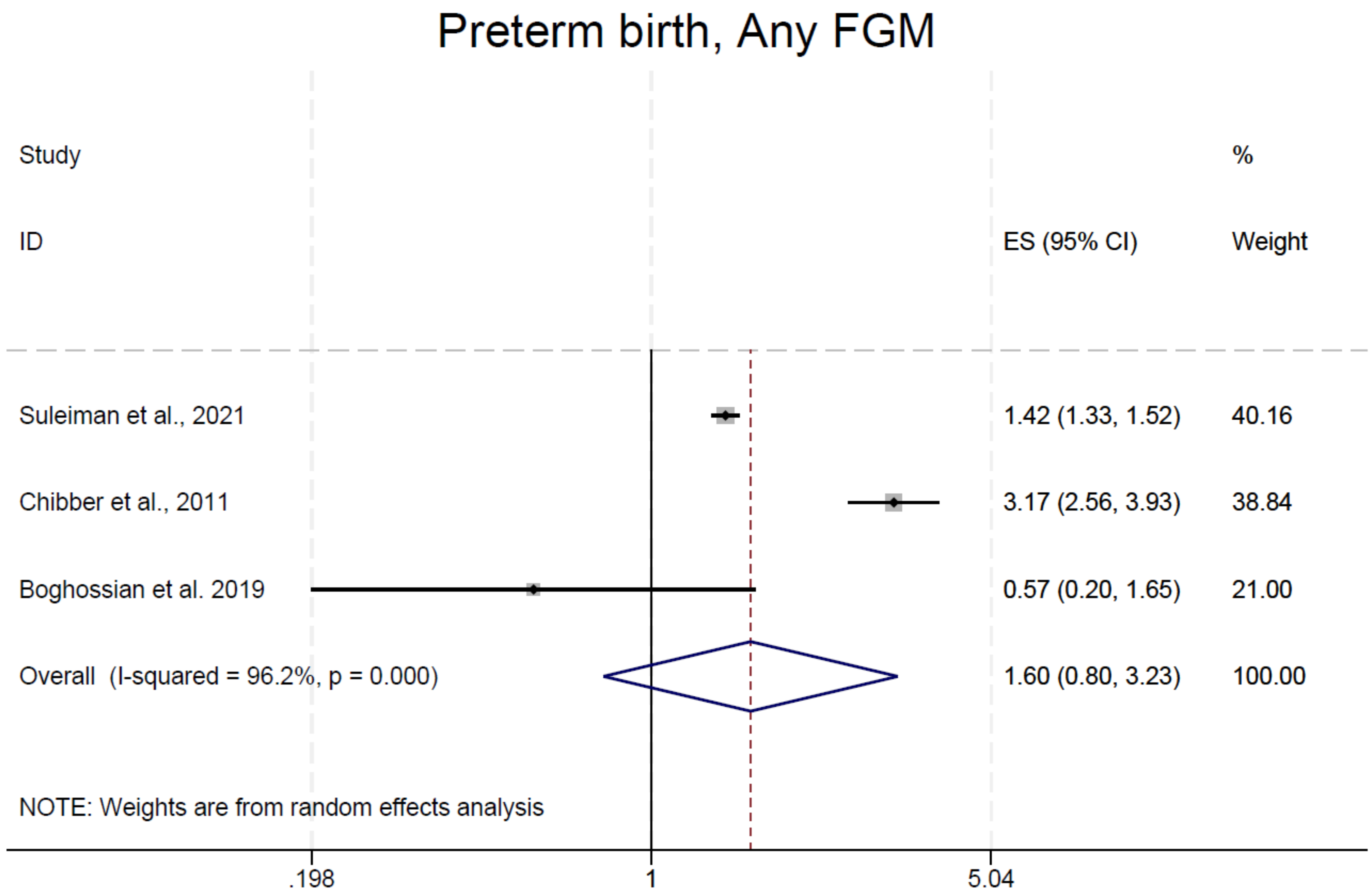

Preterm birth, Any FGM
Study
ID
ES (95% CI)
%
Weight
Suleiman et al., 2021
1.42 (1.33, 1.52)
40.16
Chibber et al., 2011
3.17 (2.56, 3.93)
38.84
Boghossian et al. 2019
0.57 (0.20, 1.65)
21.00
Overall (I-squared = 96.2%, p = 0.000)
1.60 (0.80, 3.23)
100.00
NOTE: Weights are from random effects analysis
.198
1
5.04

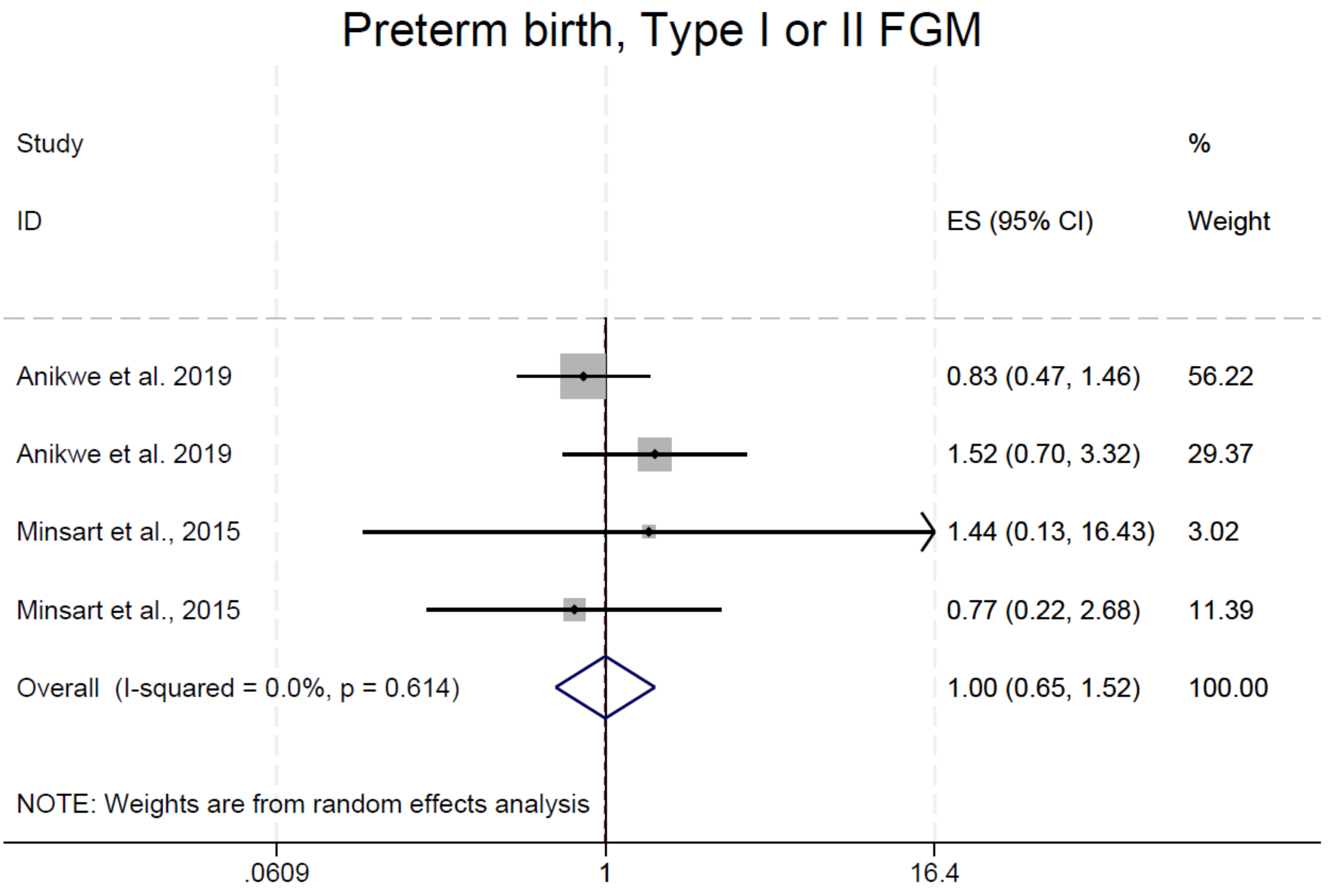
Preterm birth, Type I or II FGM
Study
ID
ES (95% CI)
%
Weight
Anikwe et al. 2019
0.83 (0.47, 1.46)
56.22
Anikwe et al. 2019
1.52 (0.70, 3.32)
29.37
Minsart et al., 2015
1.44 (0.13, 16.43)
3.02
Minsart et al., 2015
0.77 (0.22, 2.68)
11.39
Overall (I-squared = 0.0%, p = 0.614)
1.00 (0.65, 1.52)
100.00
NOTE: Weights are from random effects analysis
.0609
1
16.4

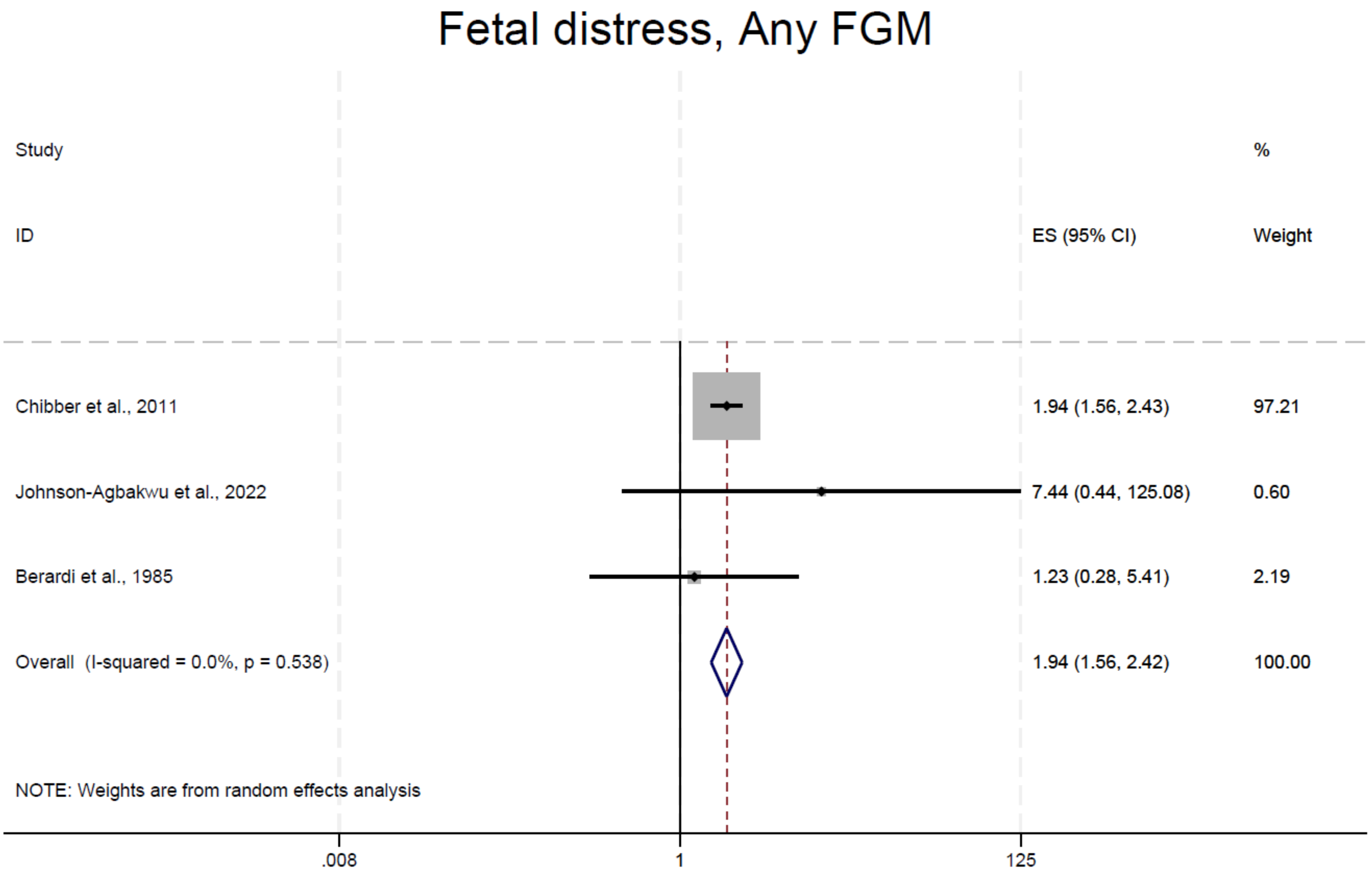
Fetal distress, Any FGM
Study
ID
ES (95% CI)
%
Weight
Chibber et al., 2011
1.94 (1.56, 2.43)
97.21
Johnson-Agbakwu et al., 2022
7.44 (0.44, 125.08)
0.60
Berardi et al., 1985
1.23 (0.28, 5.41)
2.19
Overall (I-squared = 0.0%, p = 0.538)
1.94 (1.56, 2.42)
100.00
NOTE: Weights are from random effects analysis
.008
1
125

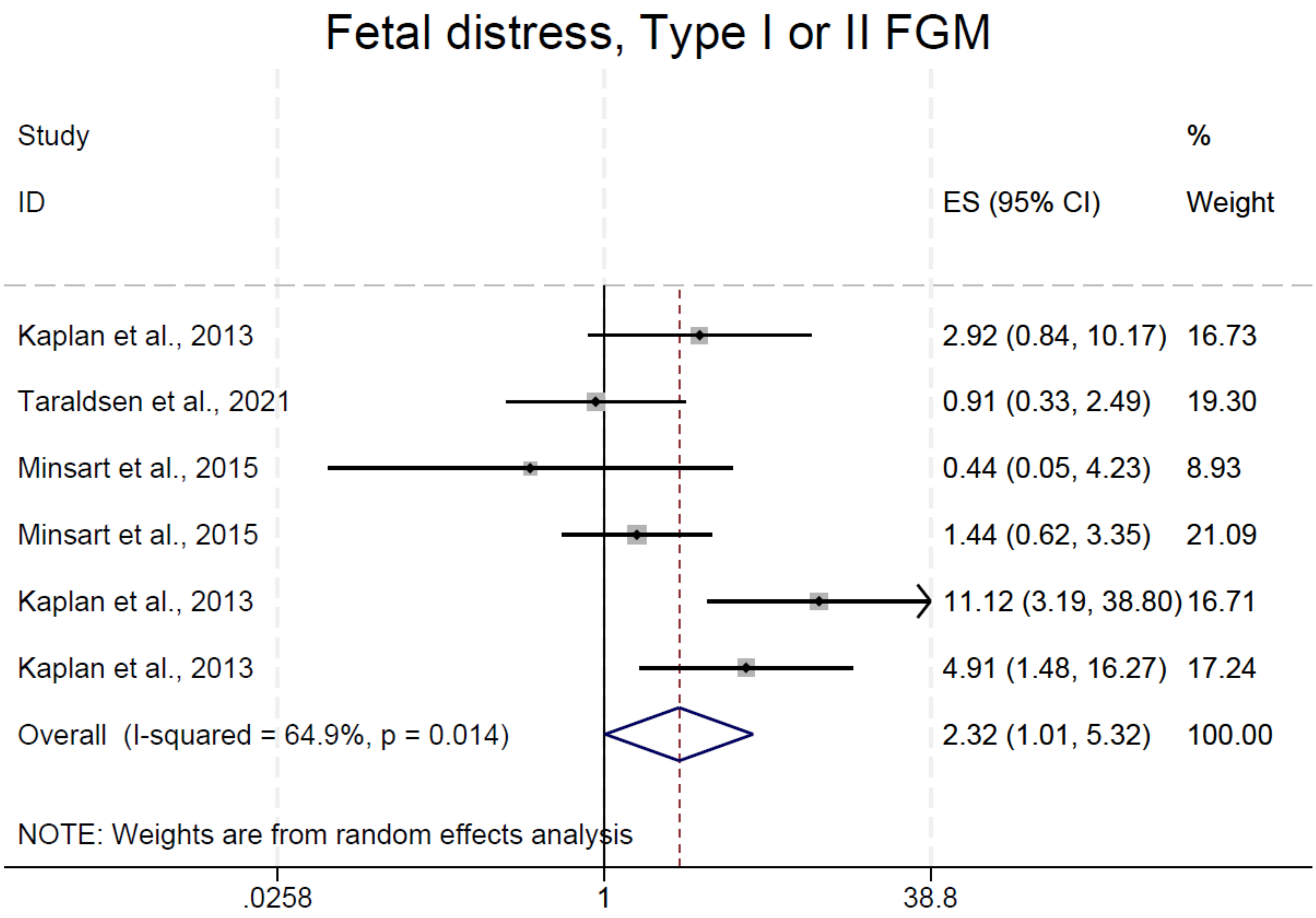
Fetal distress, Type I or II FGM
Study
ID
%
ES (95% CI)
Weight
Kaplan et al., 2013
2.92 (0.84, 10.17)
16.73
Taraldsen et al., 2021
0.91 (0.33, 2.49)
19.30
Minsart et al., 2015
0.44 (0.05, 4.23)
8.93
Minsart et al., 2015
1.44 (0.62, 3.35)
21.09
Kaplan et al., 2013
11.12 (3.19, 38.80)
16.71
Kaplan et al., 2013
4.91 (1.48, 16.27)
17.24
Overall (I-squared = 64.9%, p = 0.014)
2.32 (1.01, 5.32)
100.00
NOTE: Weights are from random effects analysis
.0258
1
38.8

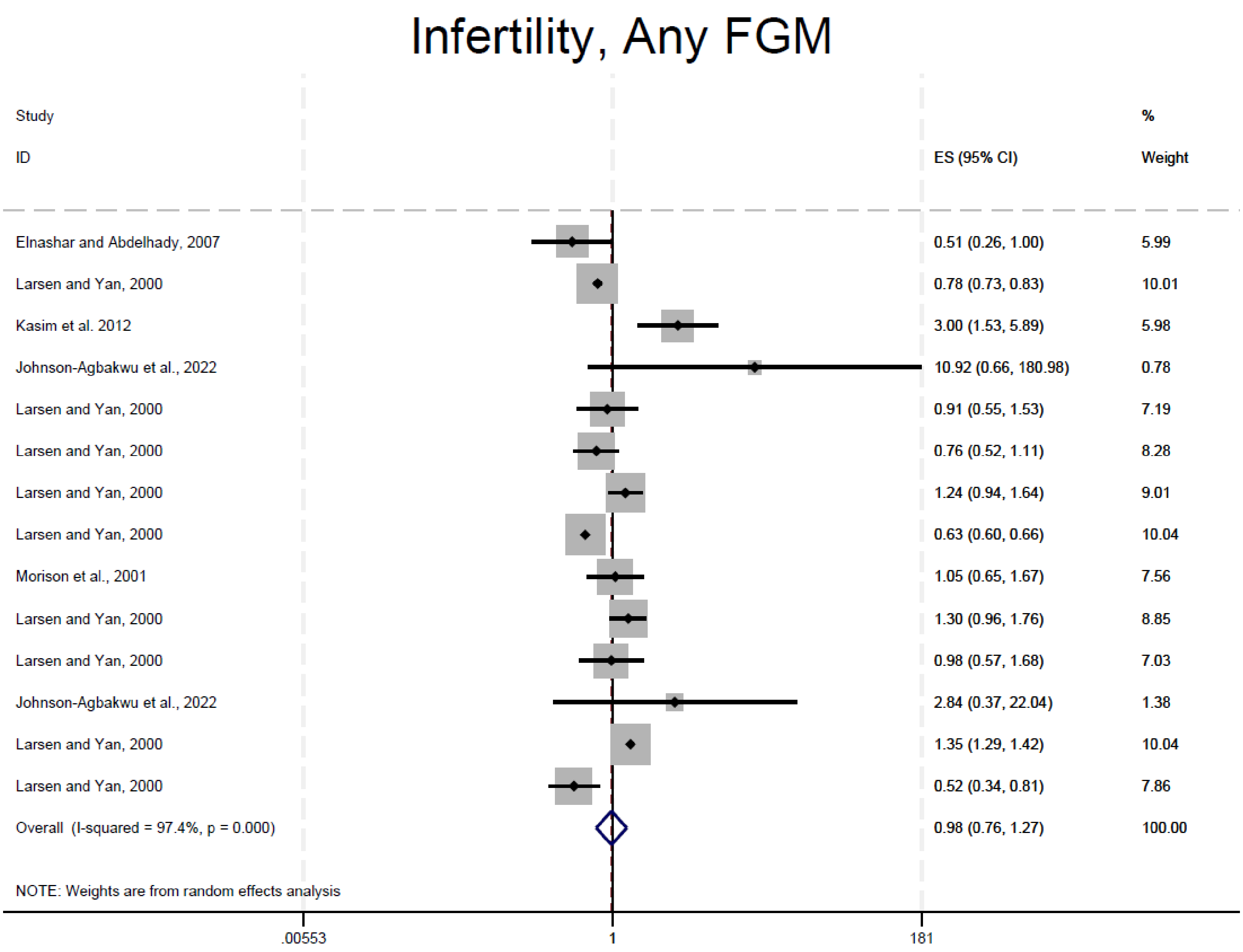
Infertility, Any FGM
Study
ID
ES (95% CI)
%
Weight
Elnashar and Abdelhady, 2007 0.51 (0.26, 1.00) 5.99
Larsen and Yan, 2000 0.78 (0.73, 0.83) 10.01
Kasim et al. 2012 3.00 (1.53, 5.89) 5.98
Johnson-Agbakwu et al., 2022 10.92 (0.66, 180.98) 0.78
Larsen and Yan, 2000 0.91 (0.55, 1.53) 7.19
Larsen and Yan, 2000 0.76 (0.52, 1.11) 8.28
Larsen and Yan, 2000 1.24 (0.94, 1.64) 9.01
Larsen and Yan, 2000 0.63 (0.60, 0.66) 10.04
Morison et al., 2001 1.05 (0.65, 1.67) 7.56
Larsen and Yan, 2000 1.30 (0.96, 1.76) 8.85
Larsen and Yan, 2000 0.98 (0.57, 1.68) 7.03
Johnson-Agbakwu et al., 2022 2.84 (0.37, 22.04) 1.38
Larsen and Yan, 2000 1.35 (1.29, 1.42) 10.04
Larsen and Yan, 2000 0.52 (0.34, 0.81) 7.86
Overall (I-squared = 97.4%, p = 0.000) 0.98 (0.76, 1.27) 100.00
NOTE: Weights are from random effects analysis
.00553
1
181

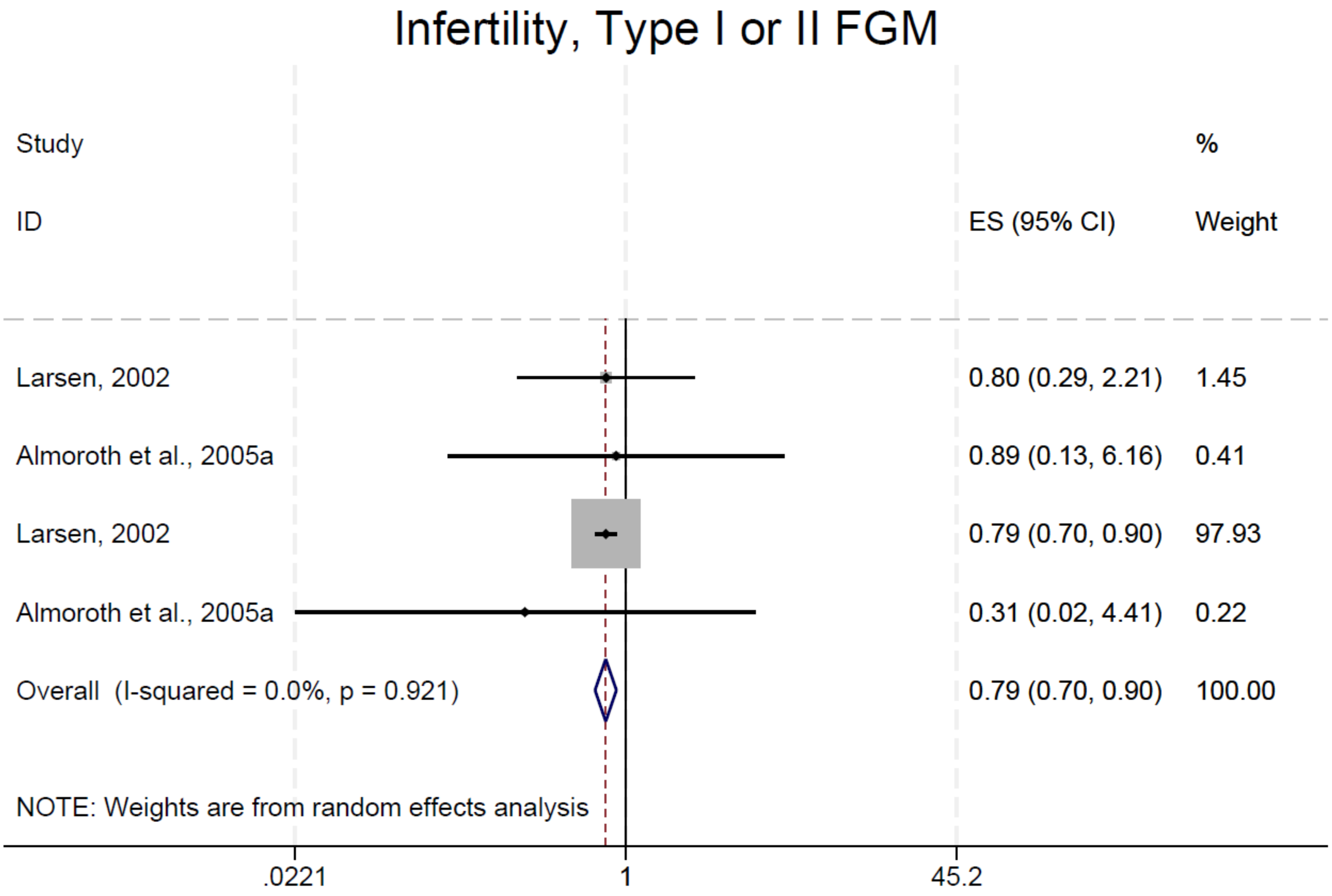
Infertility, Type I or II FGM
Study
ID
ES (95% CI)
%
Weight
Larsen, 2002
0.80 (0.29, 2.21)
1.45
Almoroth et al., 2005a
0.89 (0.13, 6.16)
0.41
Larsen, 2002
0.79 (0.70, 0.90)
97.93
Almoroth et al., 2005a
0.31 (0.02, 4.41)
0.22
Overall (I-squared = 0.0%, p = 0.921)
0.79 (0.70, 0.90)
100.00
NOTE: Weights are from random effects analysis
.0221
1
45.2

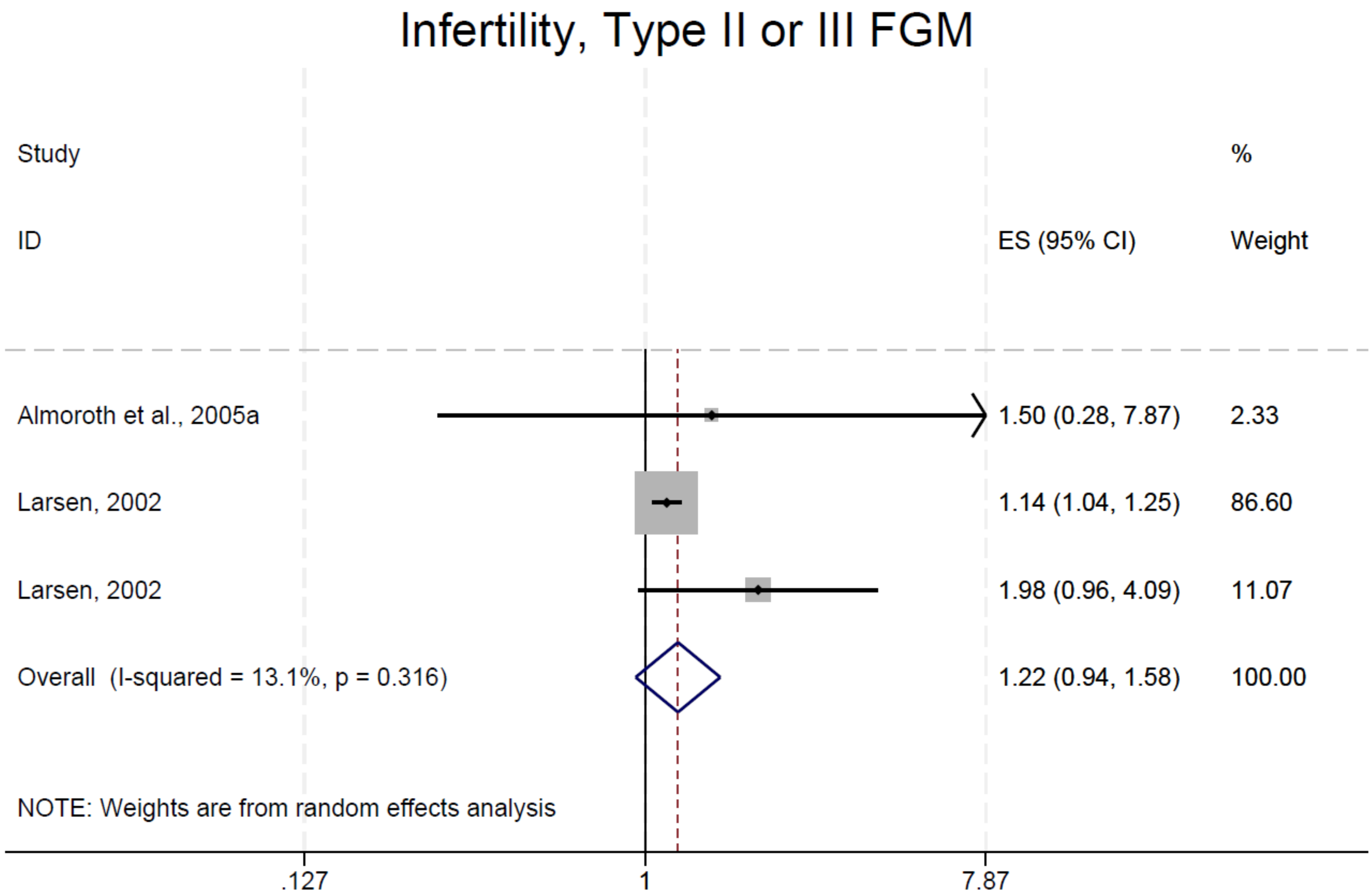
Infertility, Type II or III FGM
Study
ID
ES (95% CI)
%
Weight
Almoroth et al., 2005a
1.50 (0.28, 7.87)
2.33
Larsen, 2002
1.14 (1.04, 1.25)
86.60
Larsen, 2002
1.98 (0.96, 4.09)
11.07
Overall (I-squared = 13.1%, p = 0.316)
1.22 (0.94, 1.58)
100.00
NOTE: Weights are from random effects analysis
.127
1
7.87

Genital complications, Any FGM

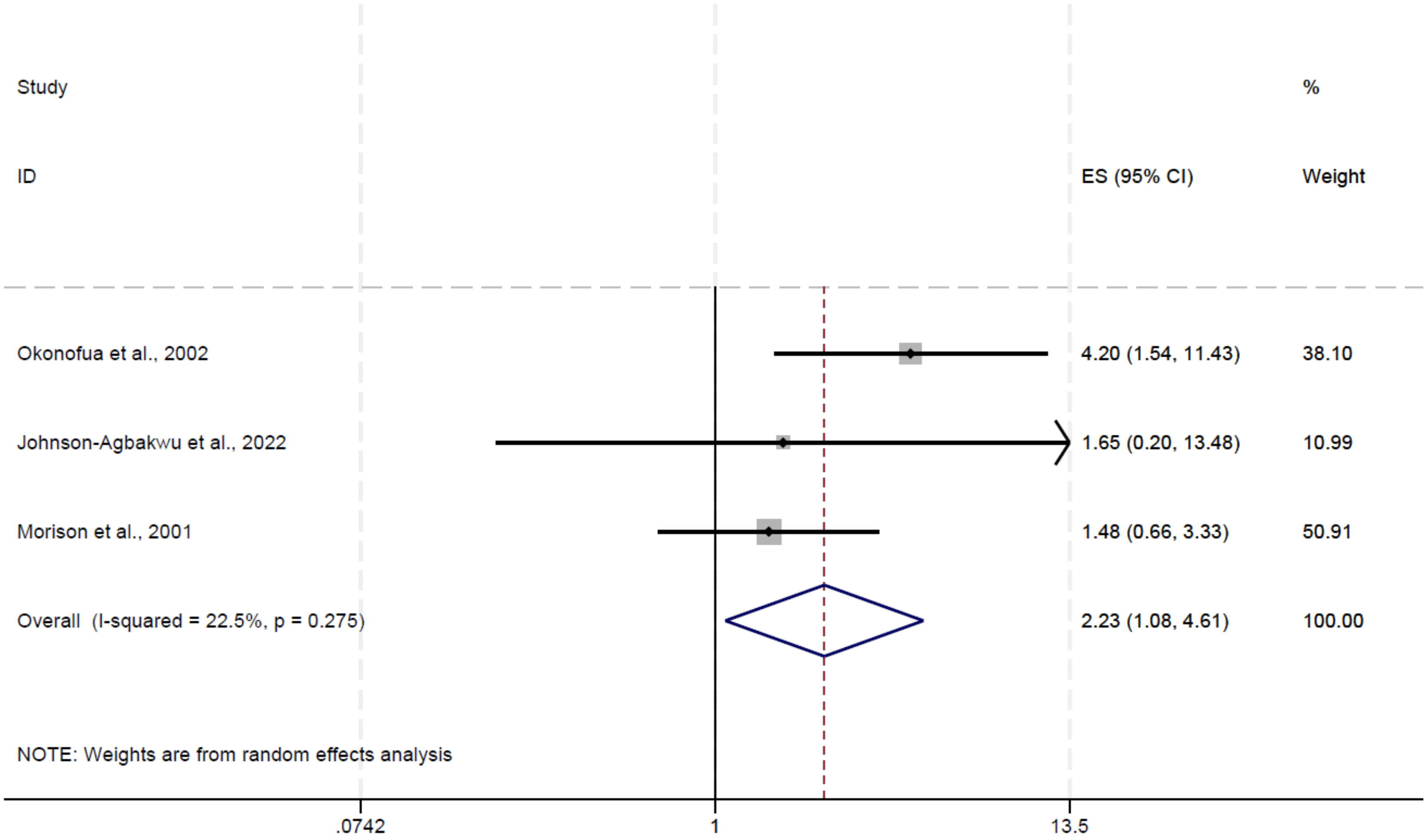

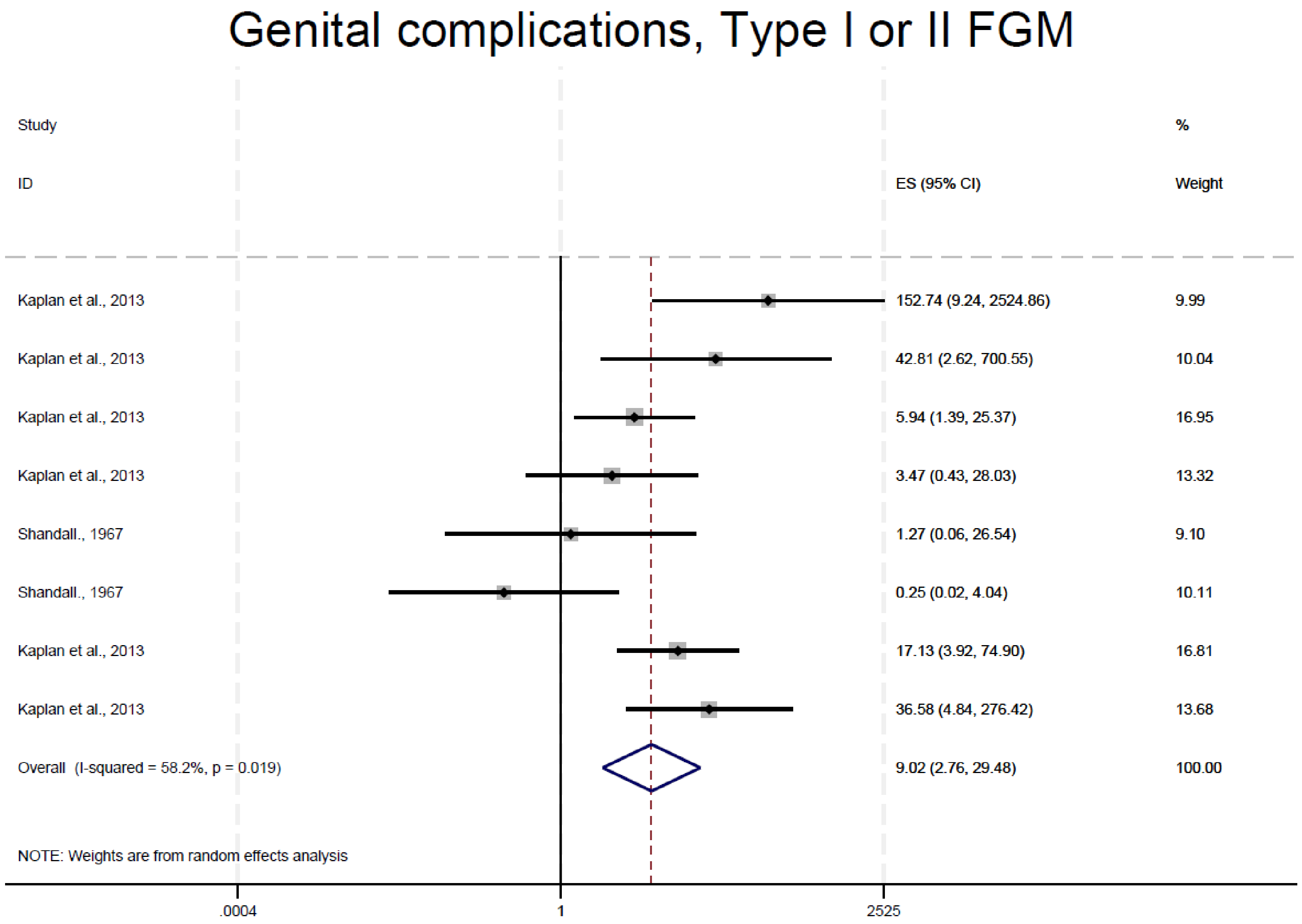

Genital complications, Type I or II FGM
Study
ID
ES (95% CI)
%
Weight
Kaplan et al., 2013
152.74 (9.24, 2524.86)
9.99
Kaplan et al., 2013
42.81 (2.62, 700.55)
10.04
Kaplan et al., 2013
5.94 (1.39, 25.37)
16.95
Kaplan et al., 2013
3.47 (0.43, 28.03)
13.32
Shandall., 1967
1.27 (0.06, 26.54)
9.10
Shandall., 1967
0.25 (0.02, 4.04)
10.11
Kaplan et al., 2013
17.13 (3.92, 74.90)
16.81
Kaplan et al., 2013
36.58 (4.84, 276.42)
13.68
Overall (I-squared = 58.2%, p = 0.019)
9.02 (2.76, 29.48)
100.00
NOTE: Weights are from random effects analysis
.0004
1
2525

Sexually Transmitted Infections, Any FGM

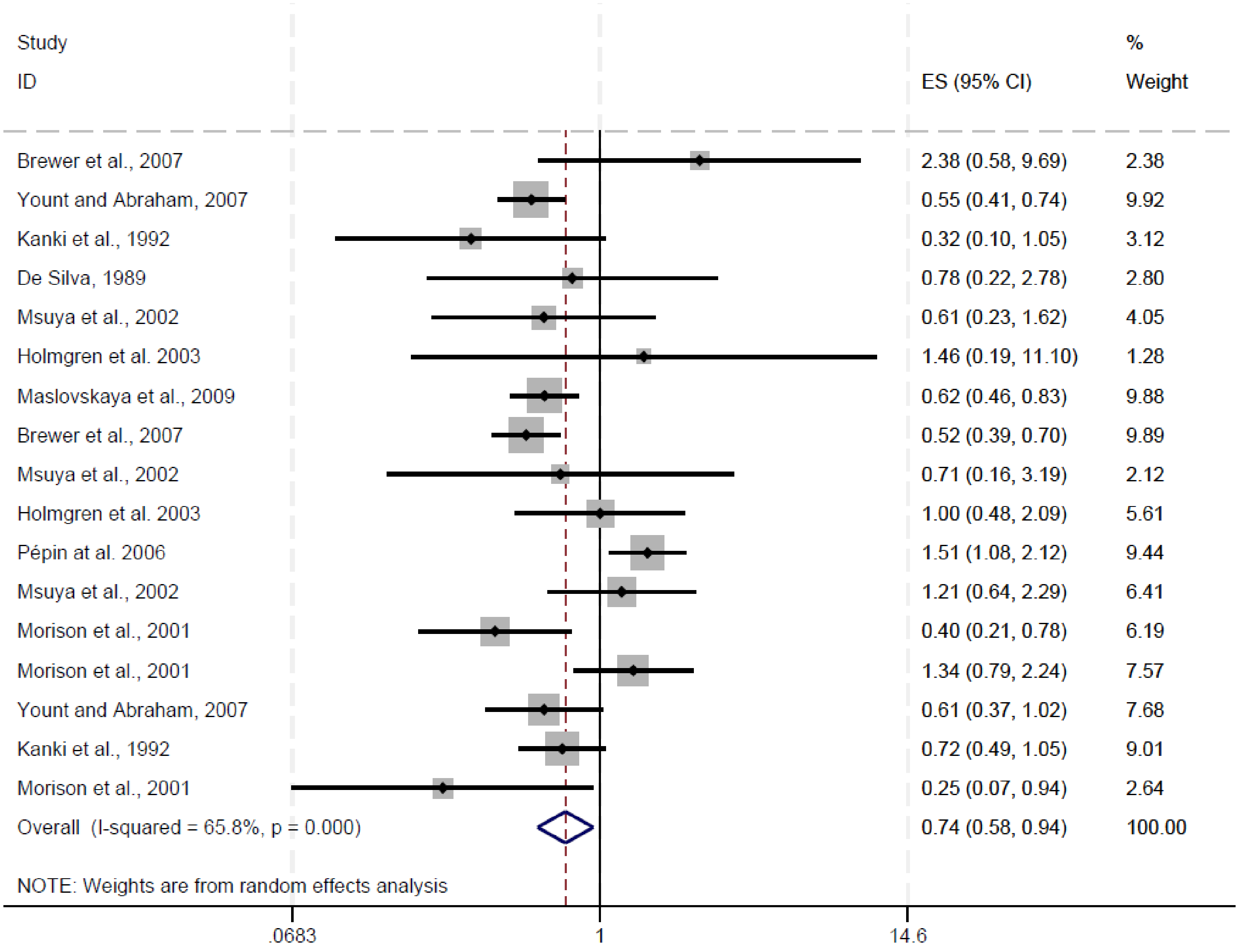

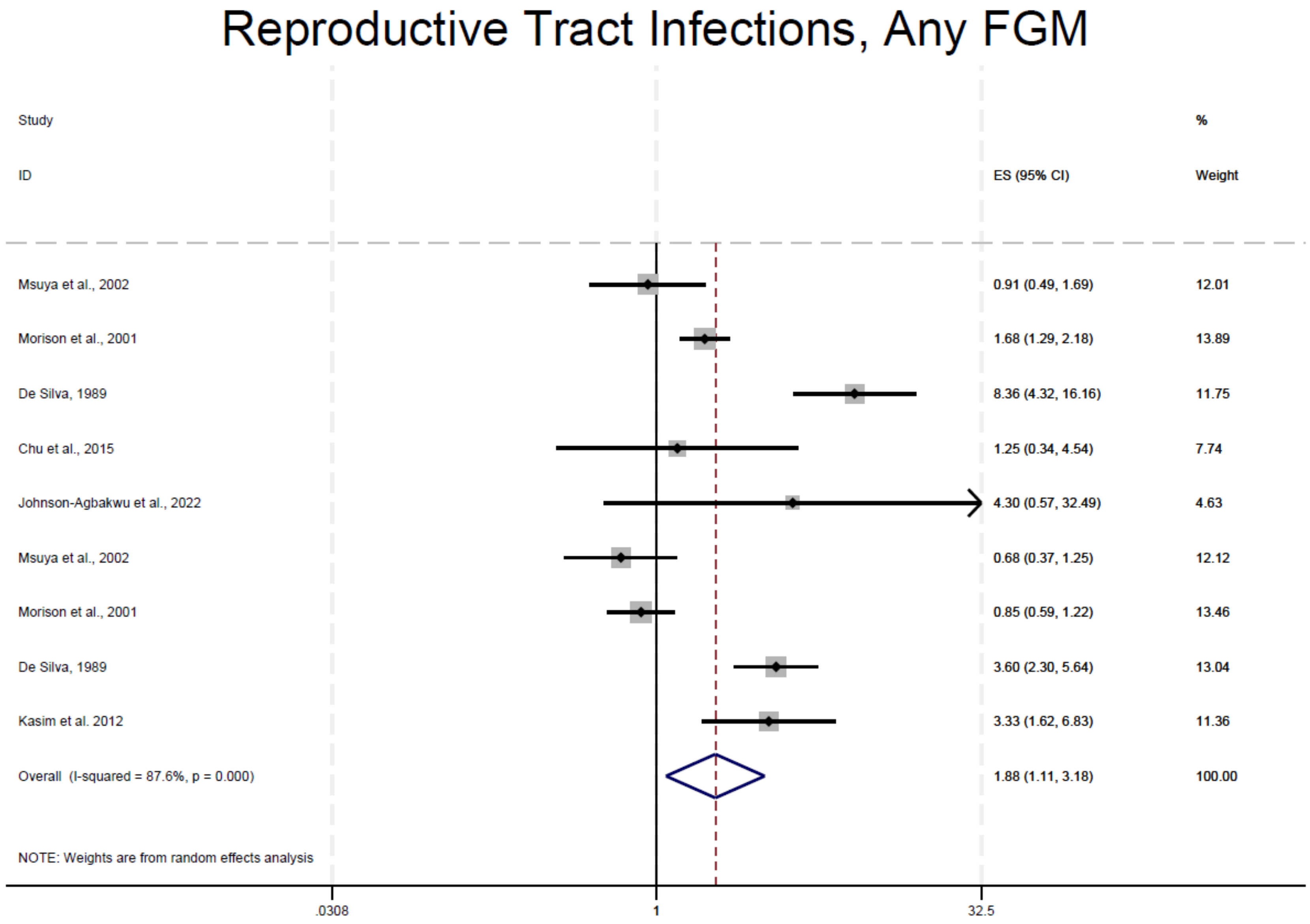

Reproductive Tract Infections, Any FGM
Study
ID
ES (95% CI)
%
Weight
Msuya et al., 2002
0.91 (0.49, 1.69)
12.01
Morison et al., 2001
1.68 (1.29, 2.18)
13.89
De Silva, 1989
8.36 (4.32, 16.16)
11.75
Chu et al., 2015
1.25 (0.34, 4.54)
7.74
Johnson-Agbakwu et al., 2022
4.30 (0.57, 32.49)
4.63
Msuya et al., 2002
0.68 (0.37, 1.25)
12.12
Morison et al., 2001
0.85 (0.59, 1.22)
13.46
De Silva, 1989
3.60 (2.30, 5.64)
13.04
Kasim et al. 2012
3.33 (1.62, 6.83)
11.36
Overall (I-squared = 87.6%, p = 0.000)
1.88 (1.11, 3.18)
100.00
NOTE: Weights are from random effects analysis
.0308
1
32.5

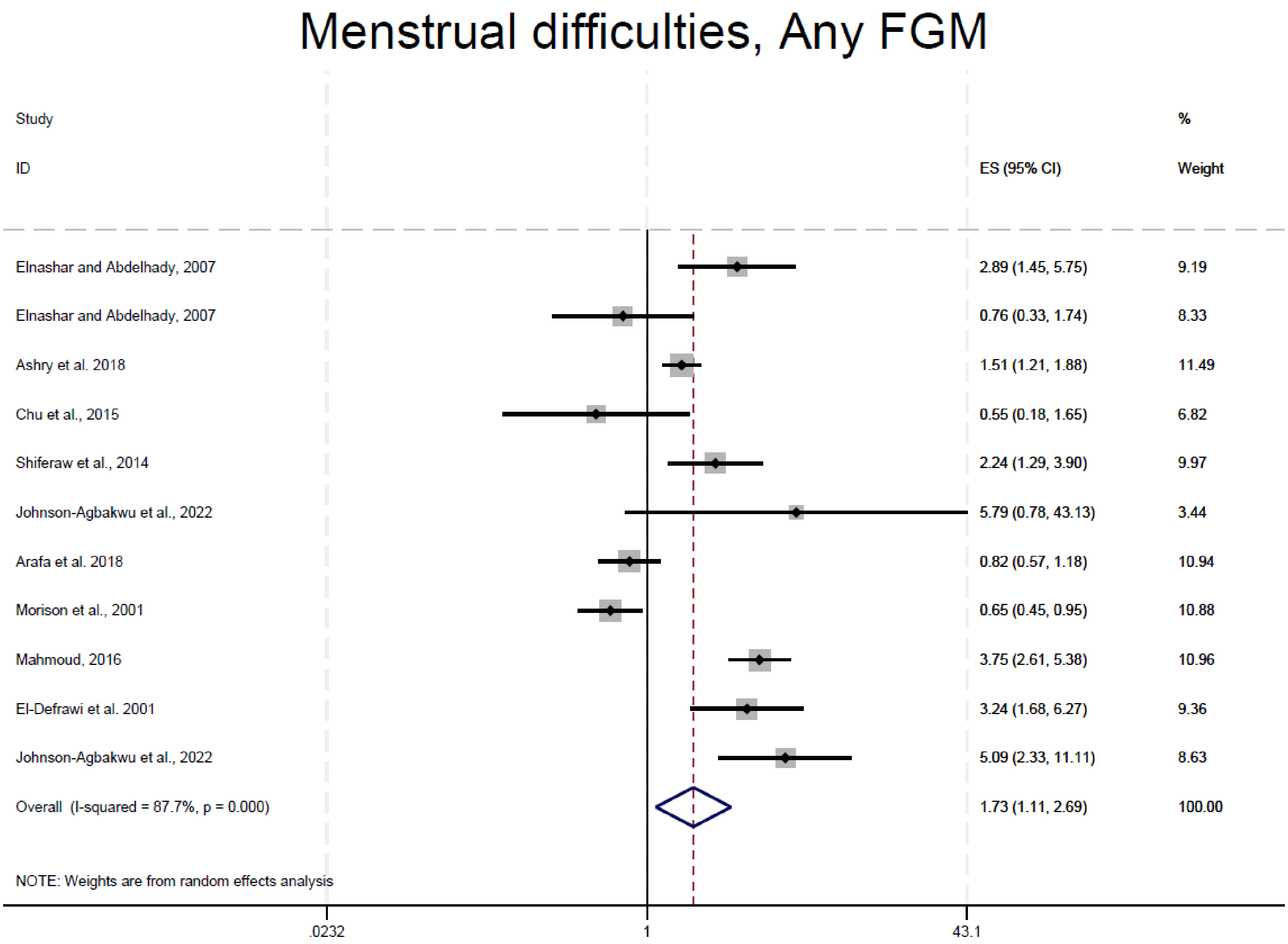
Menstrual difficulties, Any FGM
Study
ID
ES (95% CI)
%
Weight
Elnashar and Abdelhady, 2007 2.89 (1.45, 5.75) 9.19
Elnashar and Abdelhady, 2007 0.76 (0.33, 1.74) 8.33
Ashry et al. 2018 1.51 (1.21, 1.88) 11.49
Chu et al., 2015 0.55 (0.18, 1.65) 6.82
Shiferaw et al., 2014 2.24 (1.29, 3.90) 9.97
Johnson-Agbakwu et al., 2022 5.79 (0.78, 43.13) 3.44
Arafa et al. 2018 0.82 (0.57, 1.18) 10.94
Morison et al., 2001 0.65 (0.45, 0.95) 10.88
Mahmoud, 2016 3.75 (2.61, 5.38) 10.96
El-Defrawi et al. 2001 3.24 (1.68, 6.27) 9.36
Johnson-Agbakwu et al., 2022 5.09 (2.33, 11.11) 8.63
Overall (I-squared = 87.7%, p = 0.000) 1.73 (1.11, 2.69) 100.00
NOTE: Weights are from random effects analysis
.0232
1
43.1

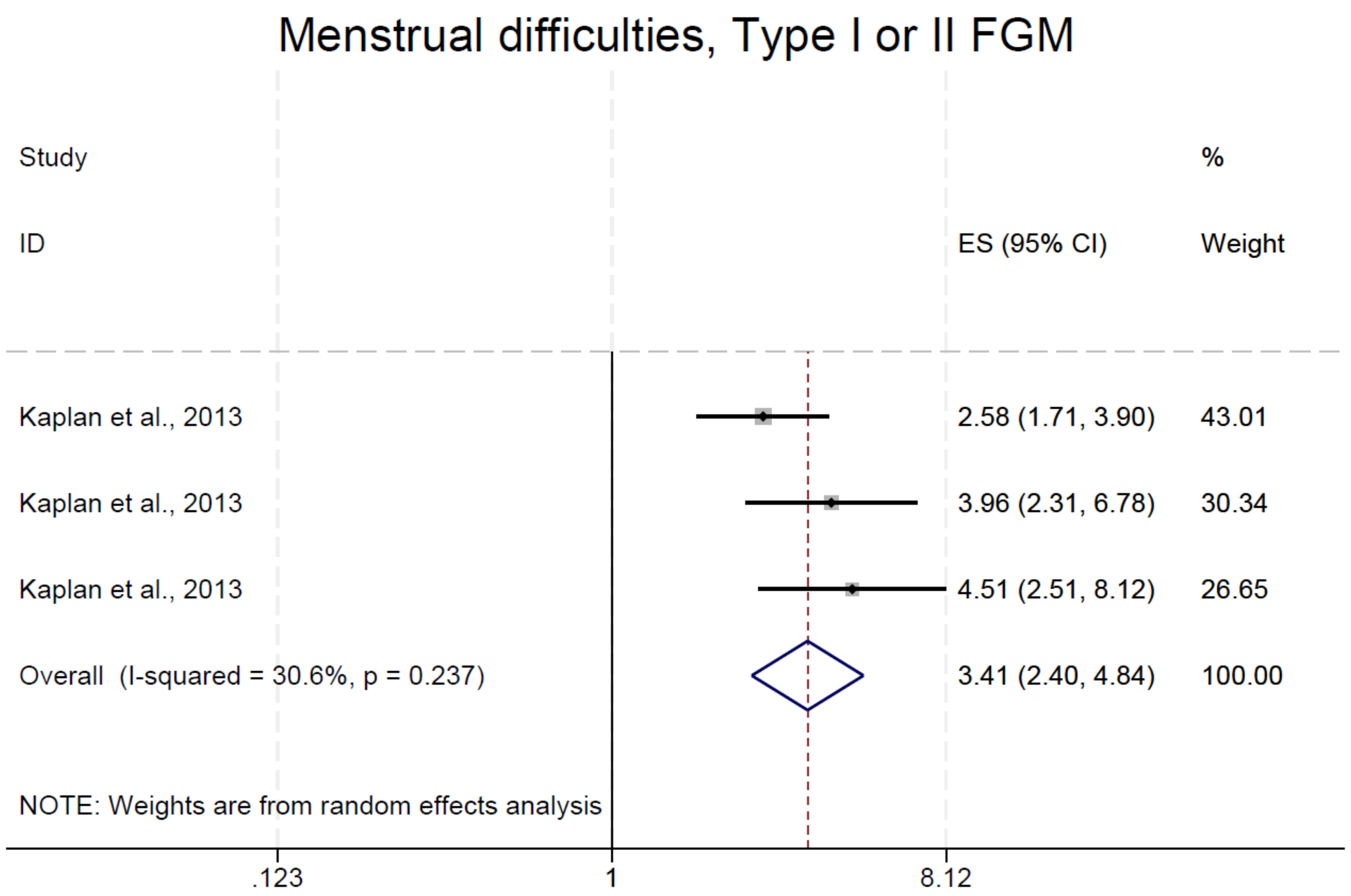
Menstrual difficulties, Type I or II FGM
Study
ID
ES (95% CI)
%
Weight
Kaplan et al., 2013
2.58 (1.71, 3.90)
43.01
Kaplan et al., 2013
3.96 (2.31, 6.78)
30.34
Kaplan et al., 2013
4.51 (2.51, 8.12)
26.65
Overall (I-squared = 30.6%, p = 0.237)
3.41 (2.40, 4.84)
100.00
NOTE: Weights are from random effects analysis
.123
1
8.12

Fistula, Any FGM

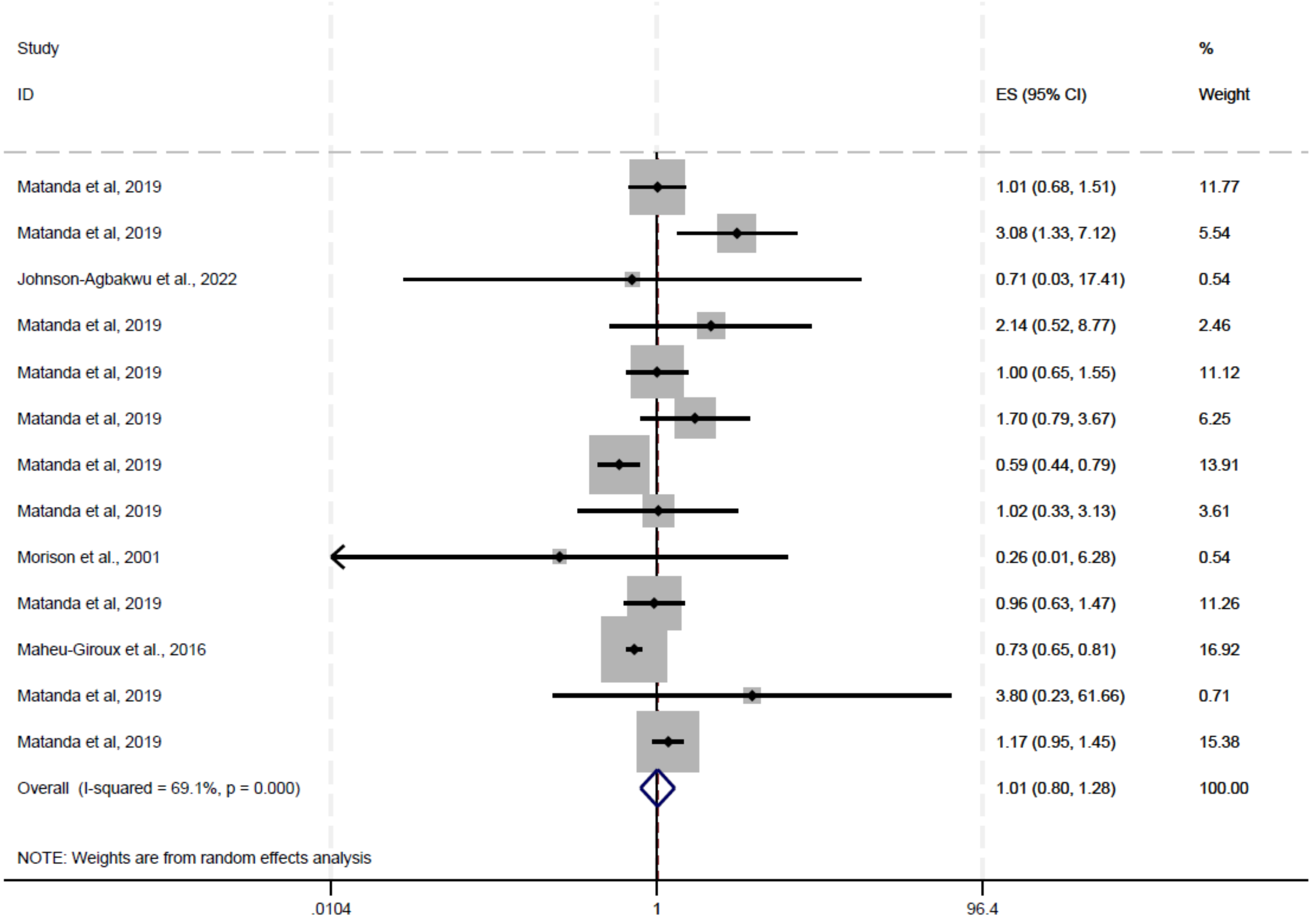

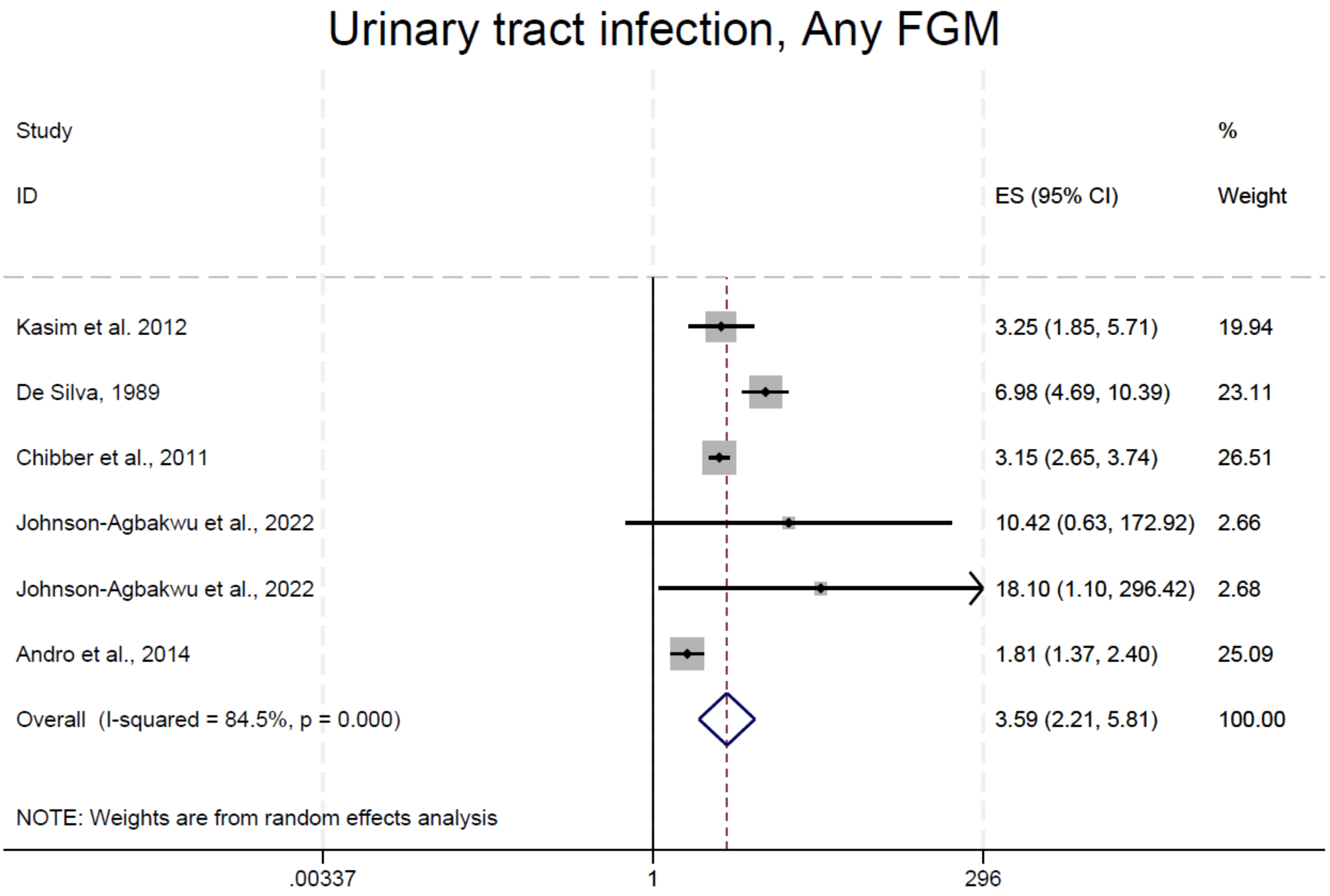

Urinary tract infection, Any FGM
Study
ID
ES (95% CI)
%
Weight
Kasim et al. 2012
3.25 (1.85, 5.71)
19.94
De Silva, 1989
6.98 (4.69, 10.39)
23.11
Chibber et al., 2011
3.15 (2.65, 3.74)
26.51
Johnson-Agbakwu et al., 2022
10.42 (0.63, 172.92)
2.66
Johnson-Agbakwu et al., 2022
18.10 (1.10, 296.42)
2.68
Andro et al., 2014
1.81 (1.37, 2.40)
25.09
Overall (I-squared = 84.5%, p = 0.000)
3.59 (2.21, 5.81)
100.00
NOTE: Weights are from random effects analysis
.00337
1
296

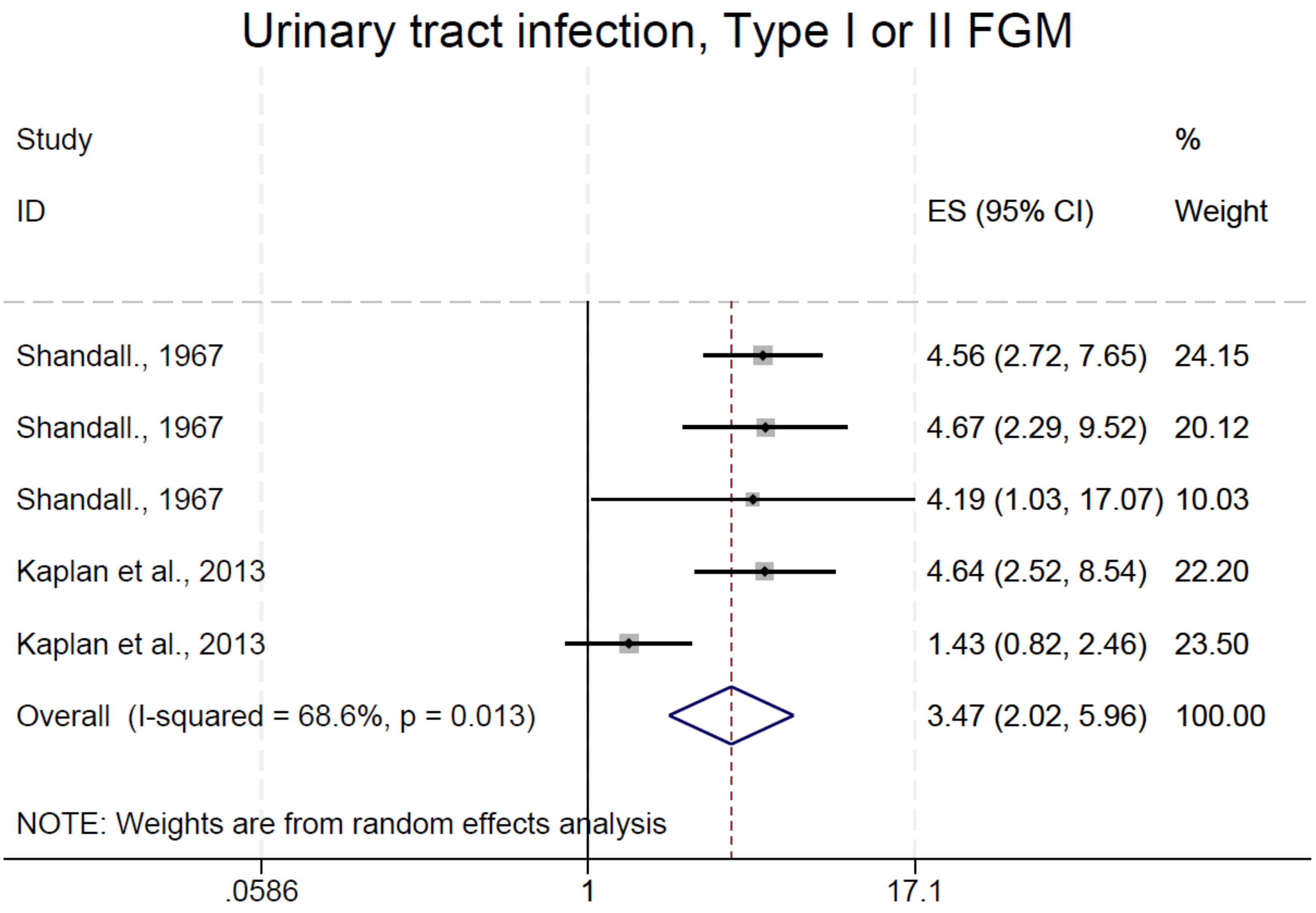
Urinary tract infection, Type I or II FGM
Study
ID
%
ES (95% CI)
Weight
Shandall., 1967
4.56 (2.72, 7.65)
24.15
Shandall., 1967
4.67 (2.29, 9.52)
20.12
Shandall., 1967
4.19 (1.03, 17.07)
10.03
Kaplan et al., 2013
4.64 (2.52, 8.54)
22.20
Kaplan et al., 2013
1.43 (0.82, 2.46)
23.50
Overall (I-squared = 68.6%, p = 0.013)
3.47 (2.02, 5.96)
100.00
NOTE: Weights are from random effects analysis
.0586
1
17.1

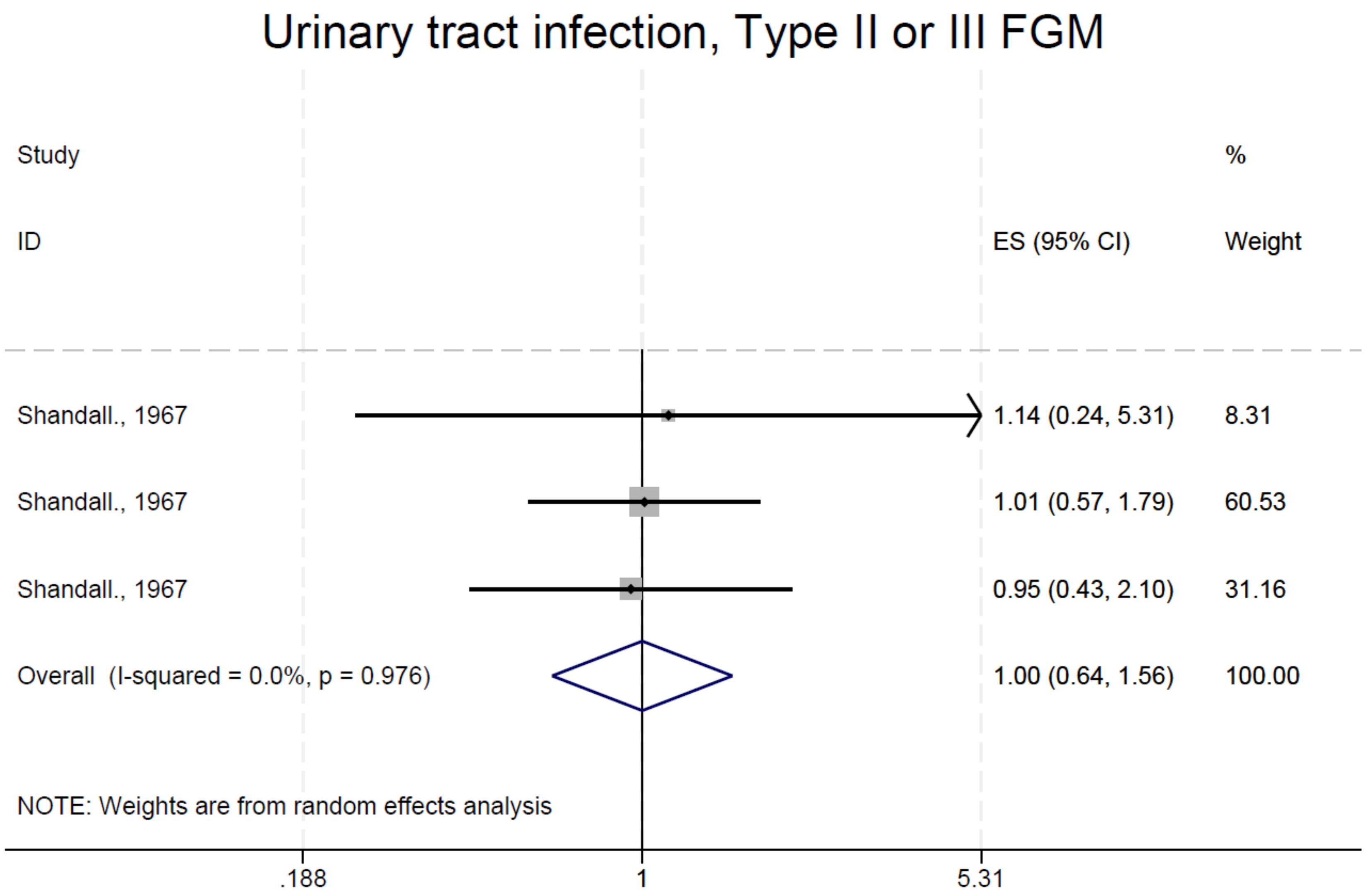
Urinary tract infection, Type II or III FGM
Study
ID
ES (95% CI)
%
Weight
Shandall., 1967
1.14 (0.24, 5.31)
8.31
Shandall., 1967
1.01 (0.57, 1.79)
60.53
Shandall., 1967
0.95 (0.43, 2.10)
31.16
Overall (I-squared = 0.0%, p = 0.976)
1.00 (0.64, 1.56)
100.00
NOTE: Weights are from random effects analysis
.188
1
5.31

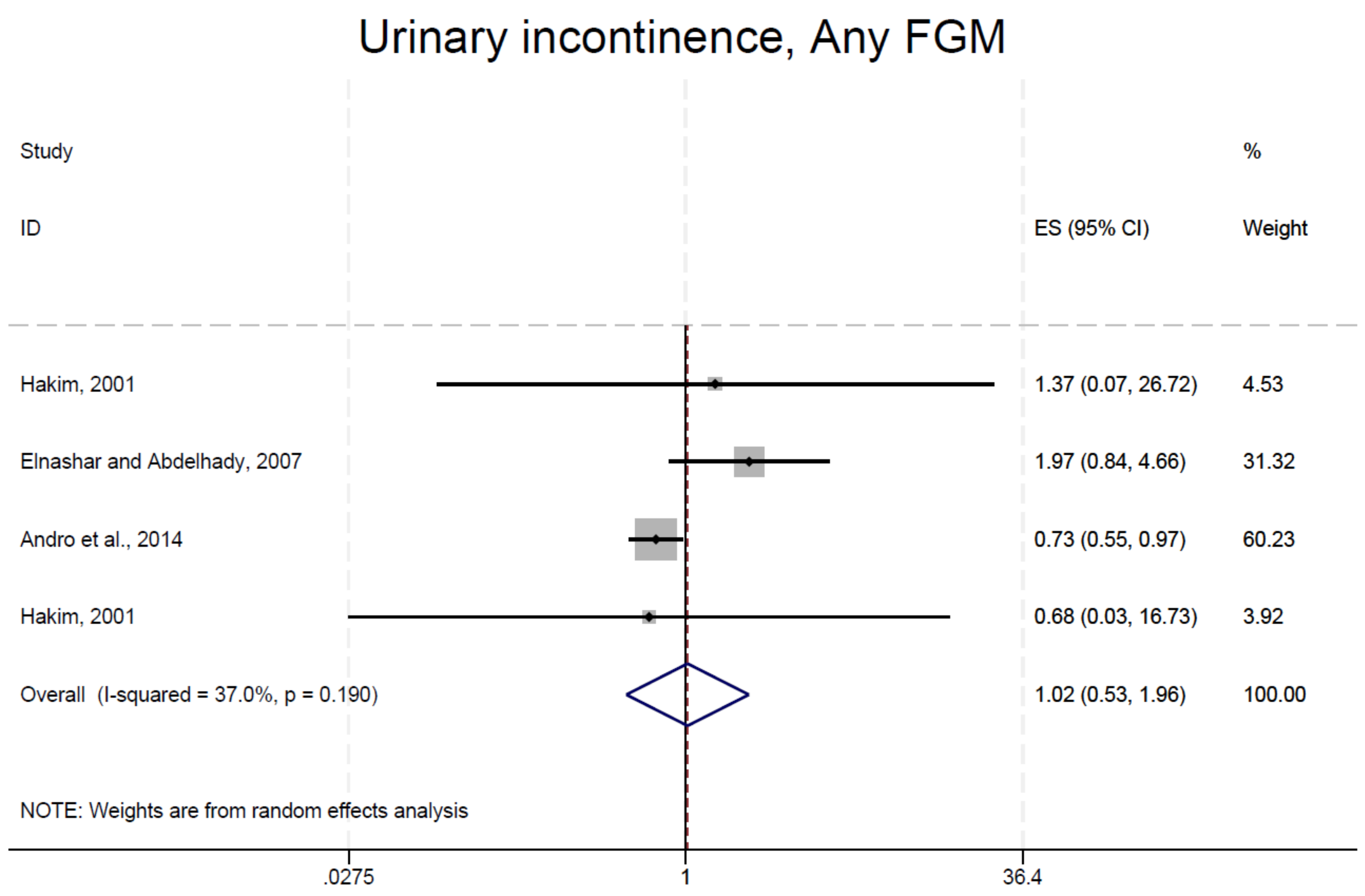
Urinary incontinence, Any FGM
Study
ID
ES (95% CI)
%
Weight
Hakim, 2001
1.37 (0.07, 26.72)
4.53
Elnashar and Abdelhady, 2007
1.97 (0.84, 4.66)
31.32
Andro et al., 2014
0.73 (0.55, 0.97)
60.23
Hakim, 2001
0.68 (0.03, 16.73)
3.92
Overall (I-squared = 37.0%, p = 0.190)
1.02 (0.53, 1.96)
100.00
NOTE: Weights are from random effects analysis
.0275
1
36.4

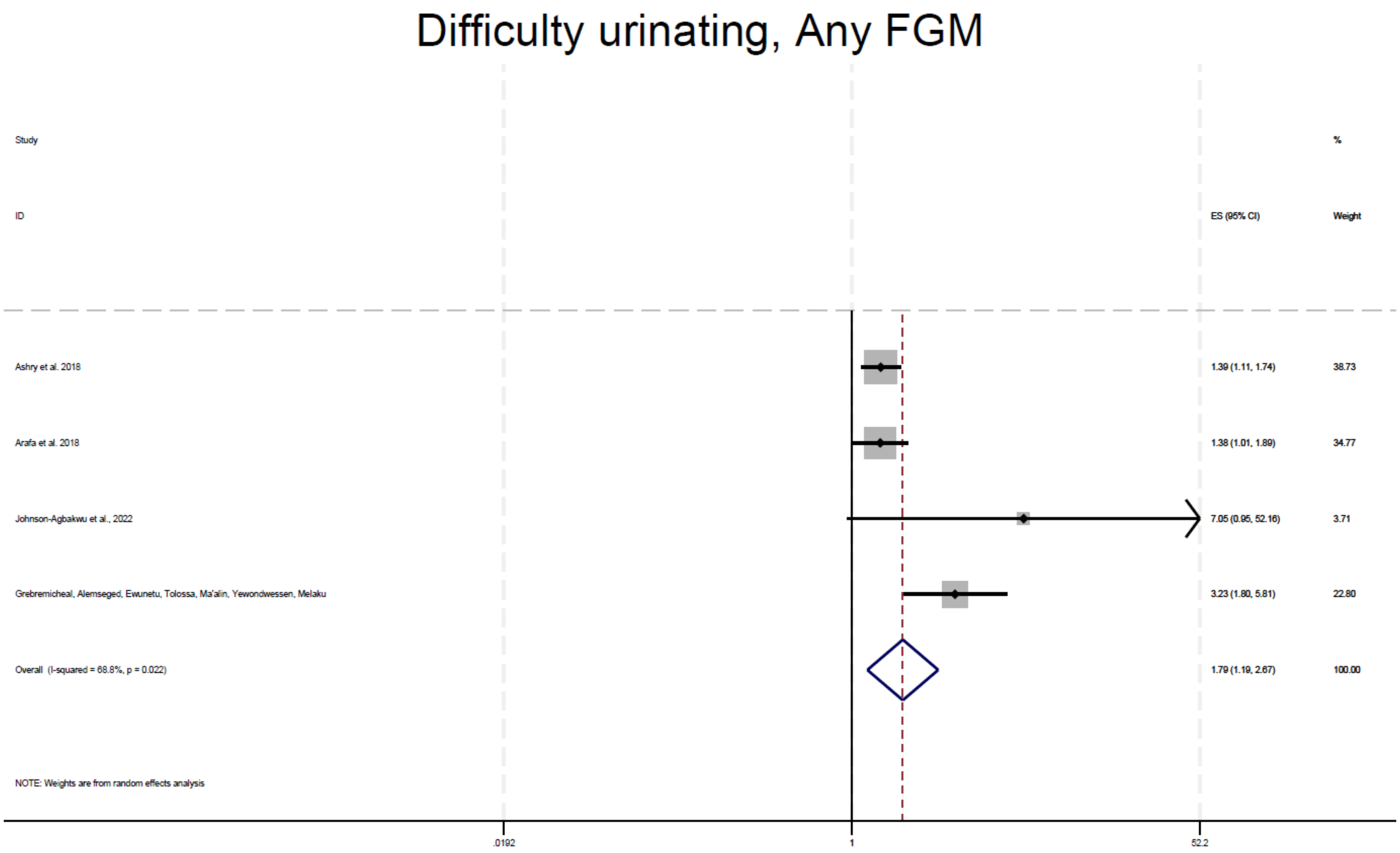
Difficulty urinating, Any FGM
Study
ID
ES (95% CI)
%
Weight
Ashry et al. 2018
1.39 (1.11, 1.74)
38.73
Arafa et al. 2018
1.38 (1.01, 1.89)
34.77
Johnson-Agbakwu et al., 2022
7.05 (0.95, 52.16)
3.71
Grebremicheal, Alemseged, Ewunetu, Tolossa, Ma'alin, Yewondwessen, Melaku
3.23 (1.80, 5.81)
22.80
Overall (I-squared = 68.8%, p = 0.022)
1.79 (1.19, 2.67)
100.00
NOTE: Weights are from random effects analysis
.0192
1
52.2

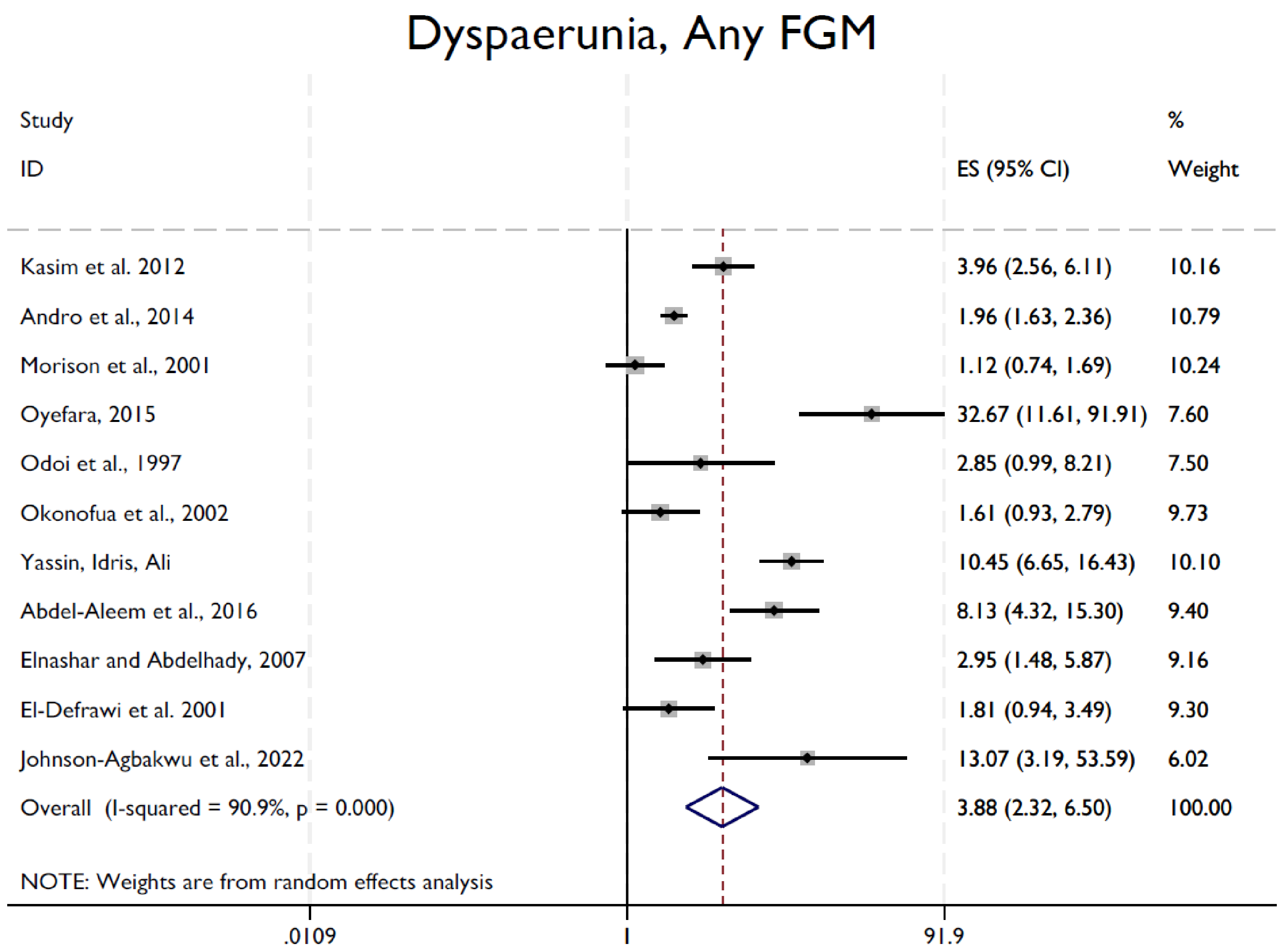
Dyspaerunia, Any FGM
Study
ID
ES (95% CI)
%
Weight
Kasim et al. 2012
3.96 (2.56, 6.11)
10.16
Andro et al., 2014
1.96 (1.63, 2.36)
10.79
Morison et al., 2001
1.12 (0.74, 1.69)
10.24
Oyefara, 2015
32.67 (11.61, 91.91)
7.60
Odoi et al., 1997
2.85 (0.99, 8.21)
7.50
Okonofua et al., 2002
1.61 (0.93, 2.79)
9.73
Yassin, Idris, Ali
10.45 (6.65, 16.43)
10.10
Abdel-Aleem et al., 2016
8.13 (4.32, 15.30)
9.40
Elnashar and Abdelhady, 2007
2.95 (1.48, 5.87)
9.16
El-Defrawi et al. 2001
1.81 (0.94, 3.49)
9.30
Johnson-Agbakwu et al., 2022
13.07 (3.19, 53.59)
6.02
Overall (I-squared = 90.9%, p = 0.000)
3.88 (2.32, 6.50)
100.00
NOTE: Weights are from random effects analysis
.0109
1
91.9

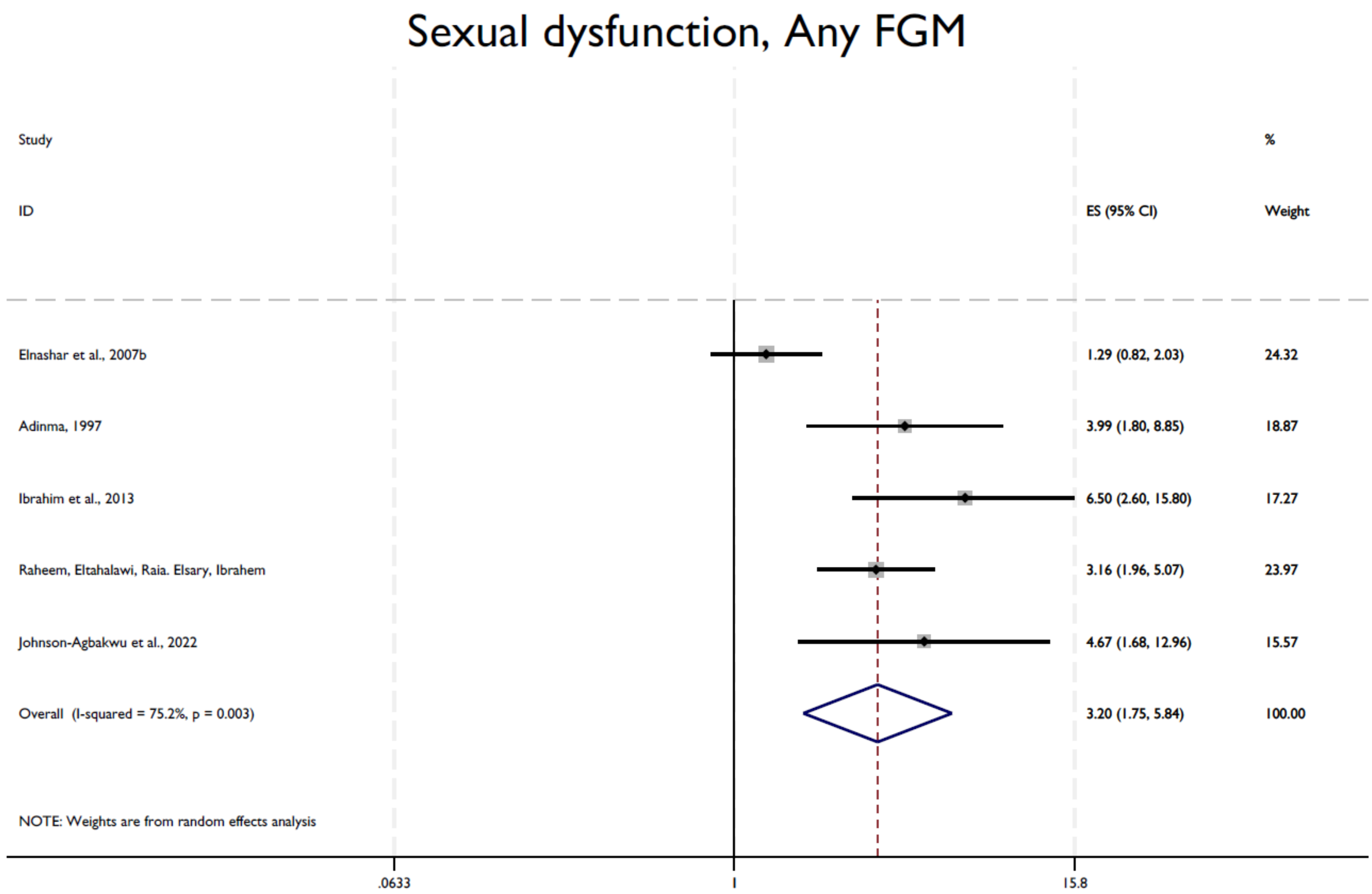
Sexual dysfunction, Any FGM
Study
ID
ES (95% CI)
%
Weight
Elnashar et al., 2007b
1.29 (0.82, 2.03)
24.32
Adinma, 1997
3.99 (1.80, 8.85)
18.87
Ibrahim et al., 2013
6.50 (2.60, 15.80)
17.27
Raheem, Eltahalawi, Raia. Elsary, Ibrahem
3.16 (1.96, 5.07)
23.97
Johnson-Agbakwu et al., 2022
4.67 (1.68, 12.96)
15.57
Overall (I-squared = 75.2%, p = 0.003)
3.20 (1.75, 5.84)
100.00
NOTE: Weights are from random effects analysis
.0633
1
15.8

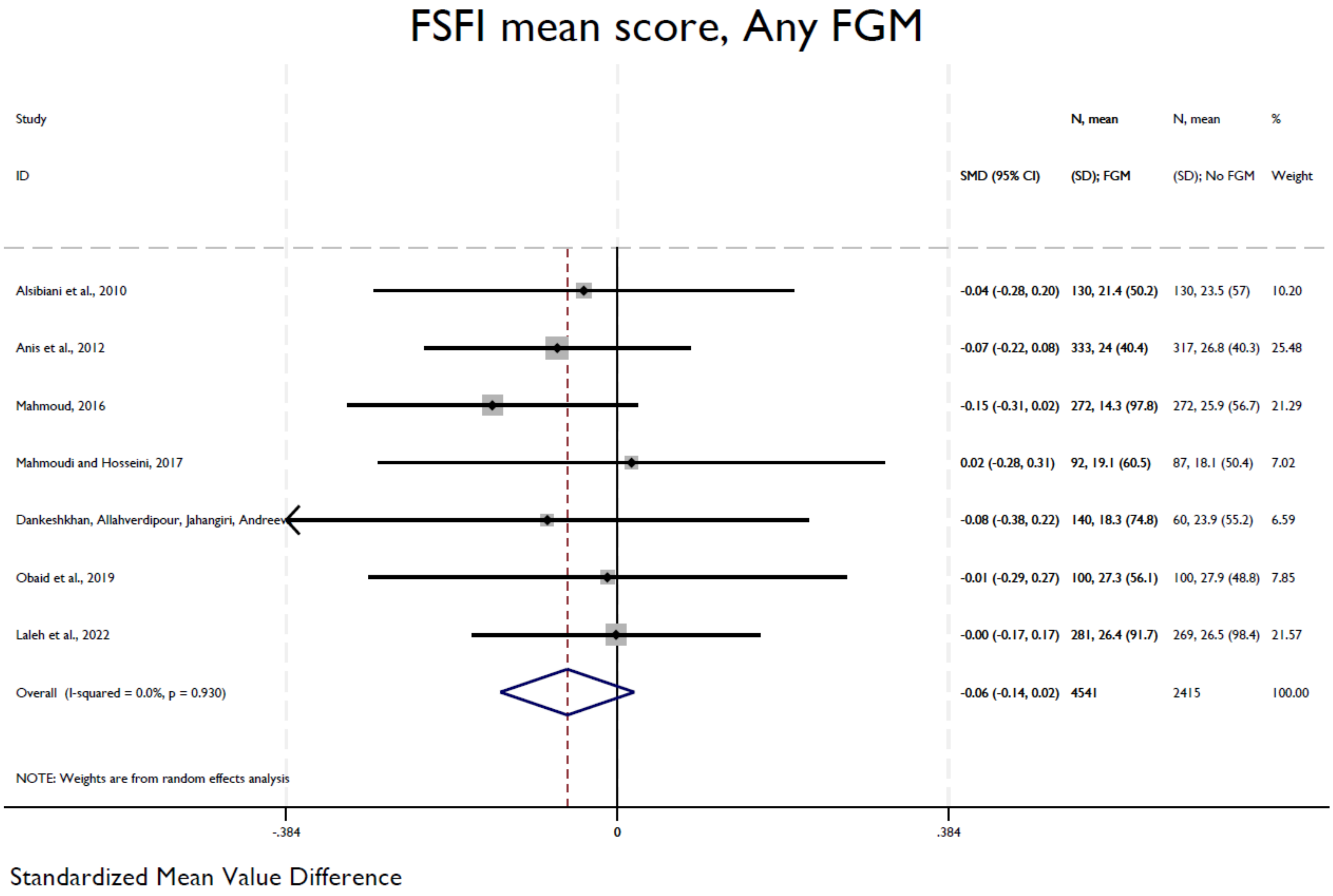
FSFI mean score, Any FGM
Study
ID
SMD (95% CI)
N, mean (SD); FGM
N, mean (SD); No FGM
% Weight
Alsibiani et al., 2010
-0.04 (-0.28, 0.20)
130, 21.4 (50.2)
130, 23.5 (57)
10.20
Anis et al., 2012
-0.07 (-0.22, 0.08)
333, 24 (40.4)
317, 26.8 (40.3)
25.48
Mahmoud, 2016
-0.15 (-0.31, 0.02)
272, 14.3 (97.8)
272, 25.9 (56.7)
21.29
Mahmoudi and Hosseini, 2017
0.02 (-0.28, 0.31)
92, 19.1 (60.5)
87, 18.1 (50.4)
7.02
Dankeshkhan, Allahverdipour, Jahangiri, Andreev
-0.08 (-0.38, 0.22)
140, 18.3 (74.8)
60, 23.9 (55.2)
6.59
Obaid et al., 2019
-0.01 (-0.29, 0.27)
100, 27.3 (56.1)
100, 27.9 (48.8)
7.85
Laleh et al., 2022
-0.00 (-0.17, 0.17)
281, 26.4 (91.7)
269, 26.5 (98.4)
21.57
Overall (I-squared = 0.0%, p = 0.930)
-0.06 (-0.14, 0.02)
4541
2415
100.00
NOTE: Weights are from random effects analysis
-.384
0
.384
Standardized Mean Value Difference

FSFI mean score, Type I or II FGM

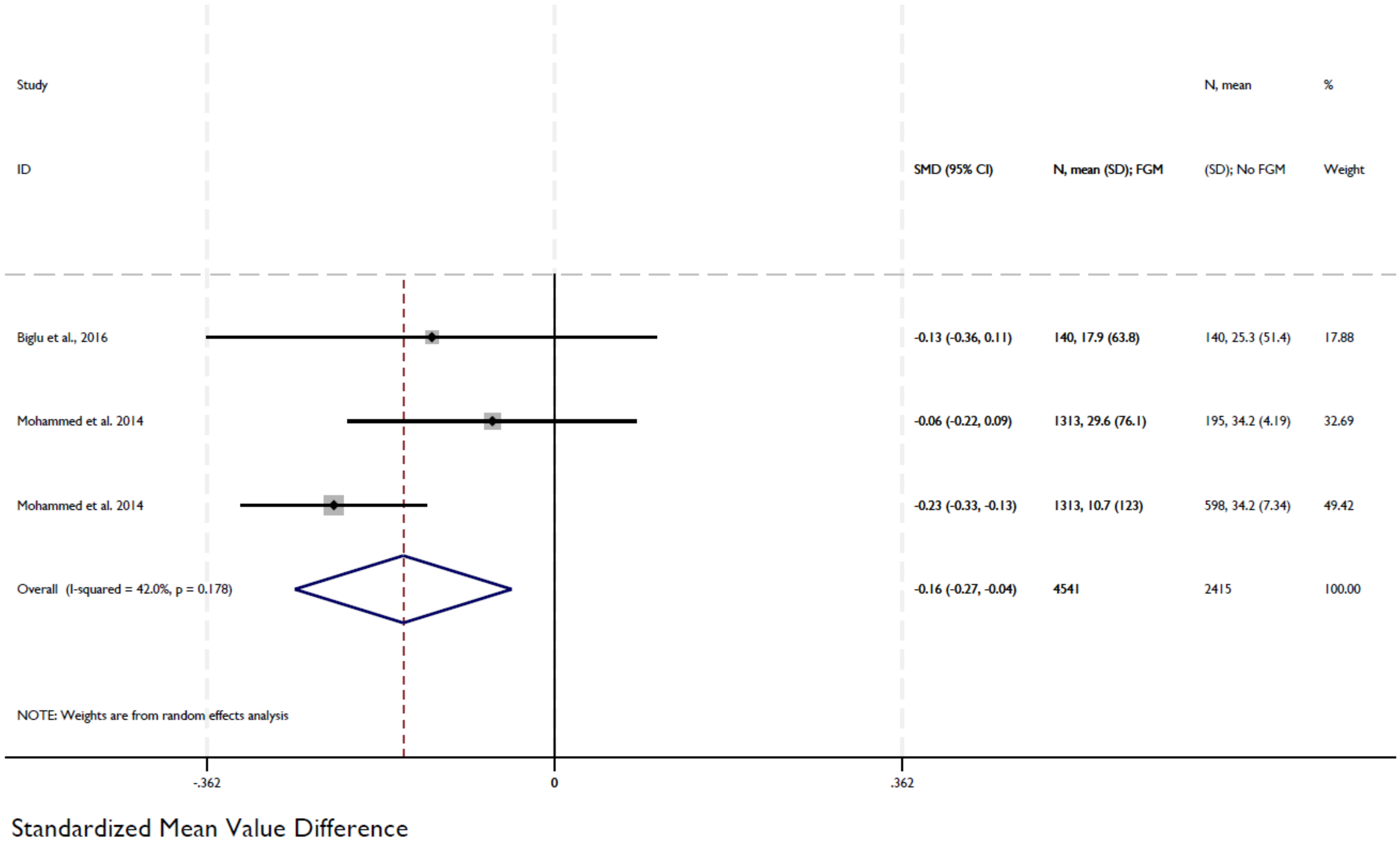

PTSD, Any FGM

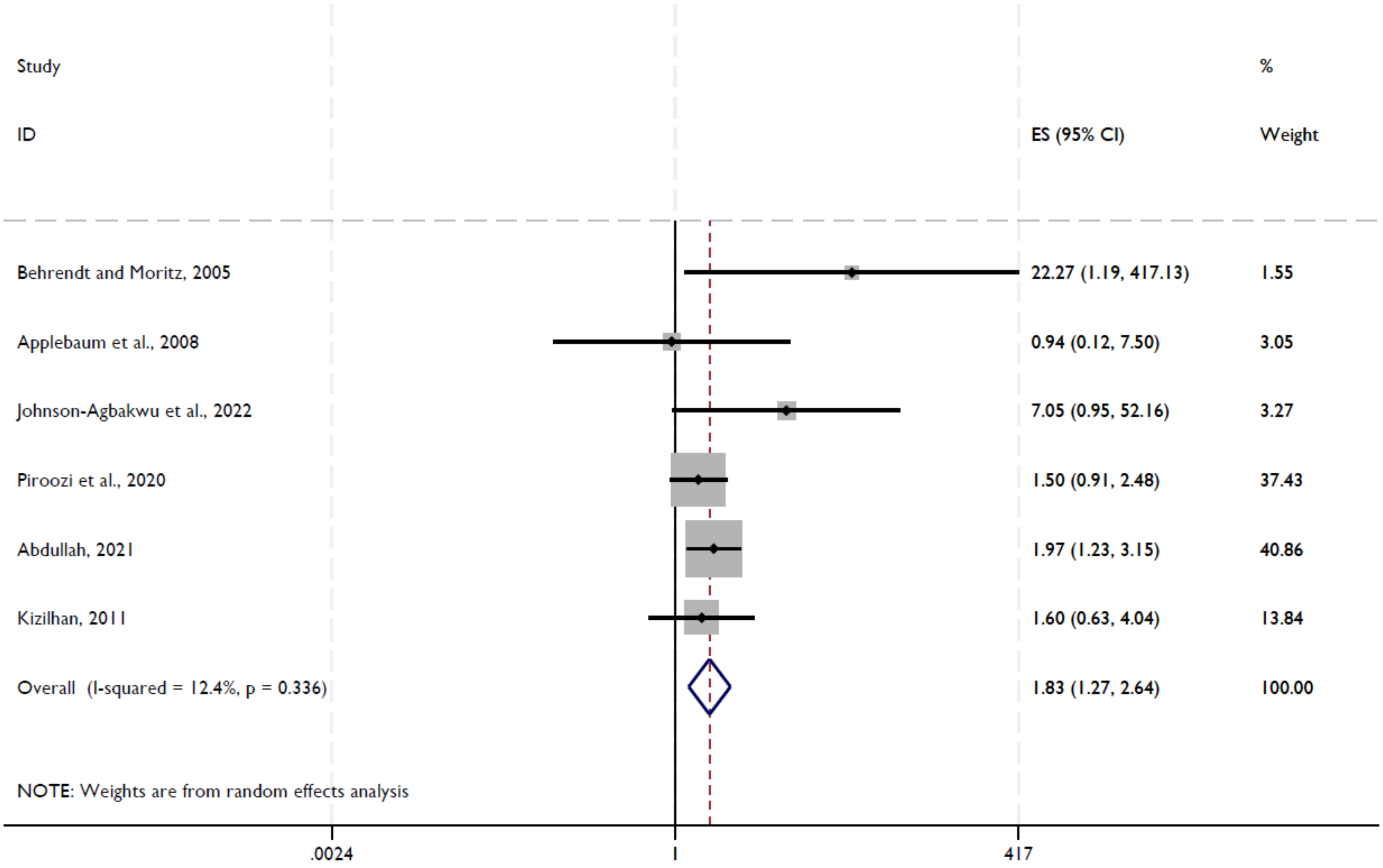
Study
ID
ES (95% CI)
%
Weight
Behrendt and Moritz, 2005
22.27 (1.19, 417.13)
1.55
Applebaum et al., 2008
0.94 (0.12, 7.50)
3.05
Johnson-Agbakwu et al., 2022
7.05 (0.95, 52.16)
3.27
Piroozi et al., 2020
1.50 (0.91, 2.48)
37.43
Abdullah, 2021
1.97 (1.23, 3.15)
40.86
Kizilhan, 2011
1.60 (0.63, 4.04)
13.84
Overall (I-squared = 12.4%, p = 0.336)
1.83 (1.27, 2.64)
100.00
NOTE: Weights are from random effects analysis
.0024
1
417

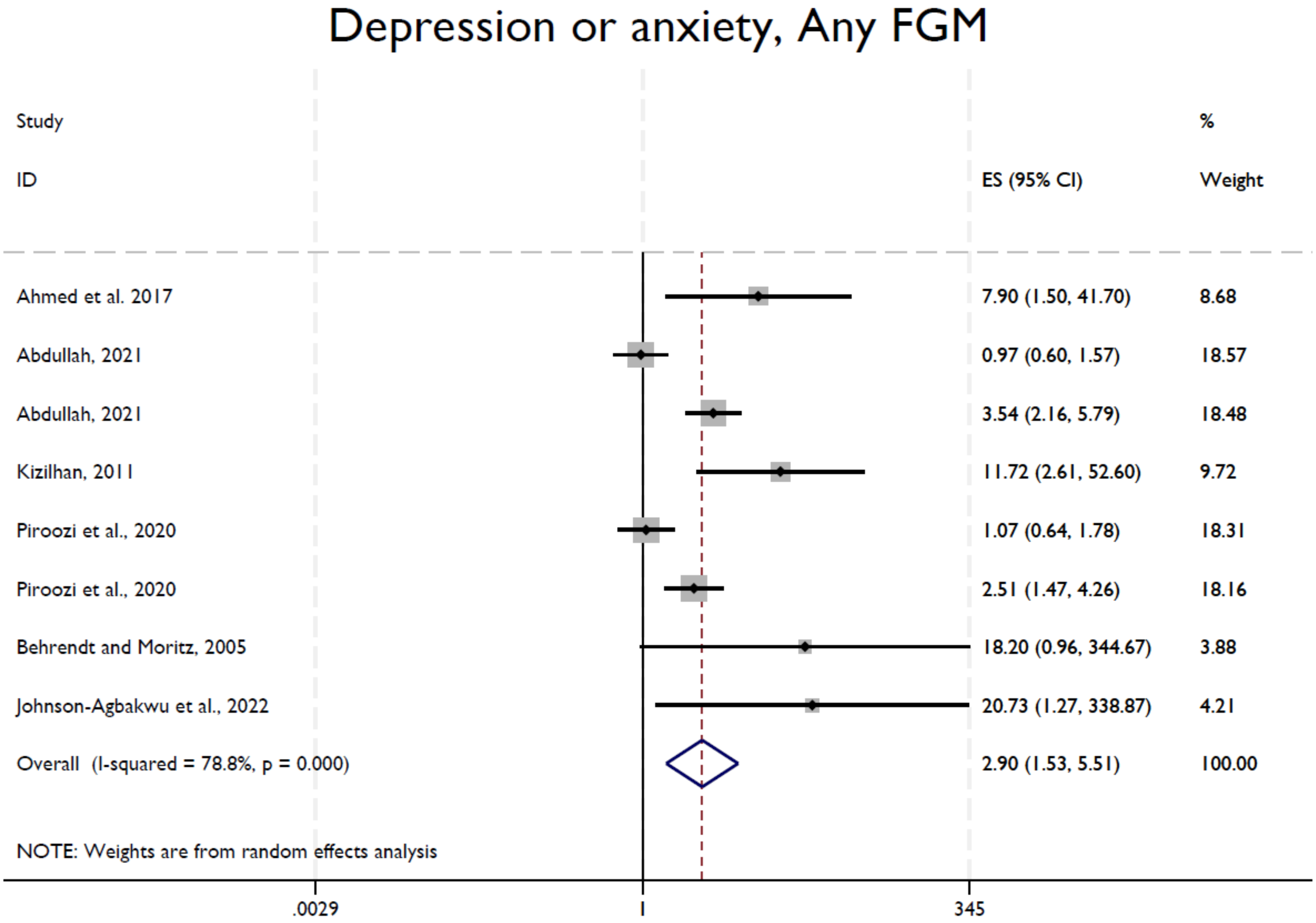
Depression or anxiety, Any FGM
Study
ID
ES (95% CI)
%
Weight
Ahmed et al. 2017
7.90 (1.50, 41.70)
8.68
Abdullah, 2021
0.97 (0.60, 1.57)
18.57
Abdullah, 2021
3.54 (2.16, 5.79)
18.48
Kizilhan, 2011
11.72 (2.61, 52.60)
9.72
Piroozi et al., 2020
1.07 (0.64, 1.78)
18.31
Piroozi et al., 2020
2.51 (1.47, 4.26)
18.16
Behrendt and Moritz, 2005
18.20 (0.96, 344.67)
3.88
Johnson-Agbakwu et al., 2022
20.73 (1.27, 338.87)
4.21
Overall (I-squared = 78.8%, p = 0.000)
2.90 (1.53, 5.51)
100.00
NOTE: Weights are from random effects analysis
.0029
1
345

Somatoform Disorder, Any FGM

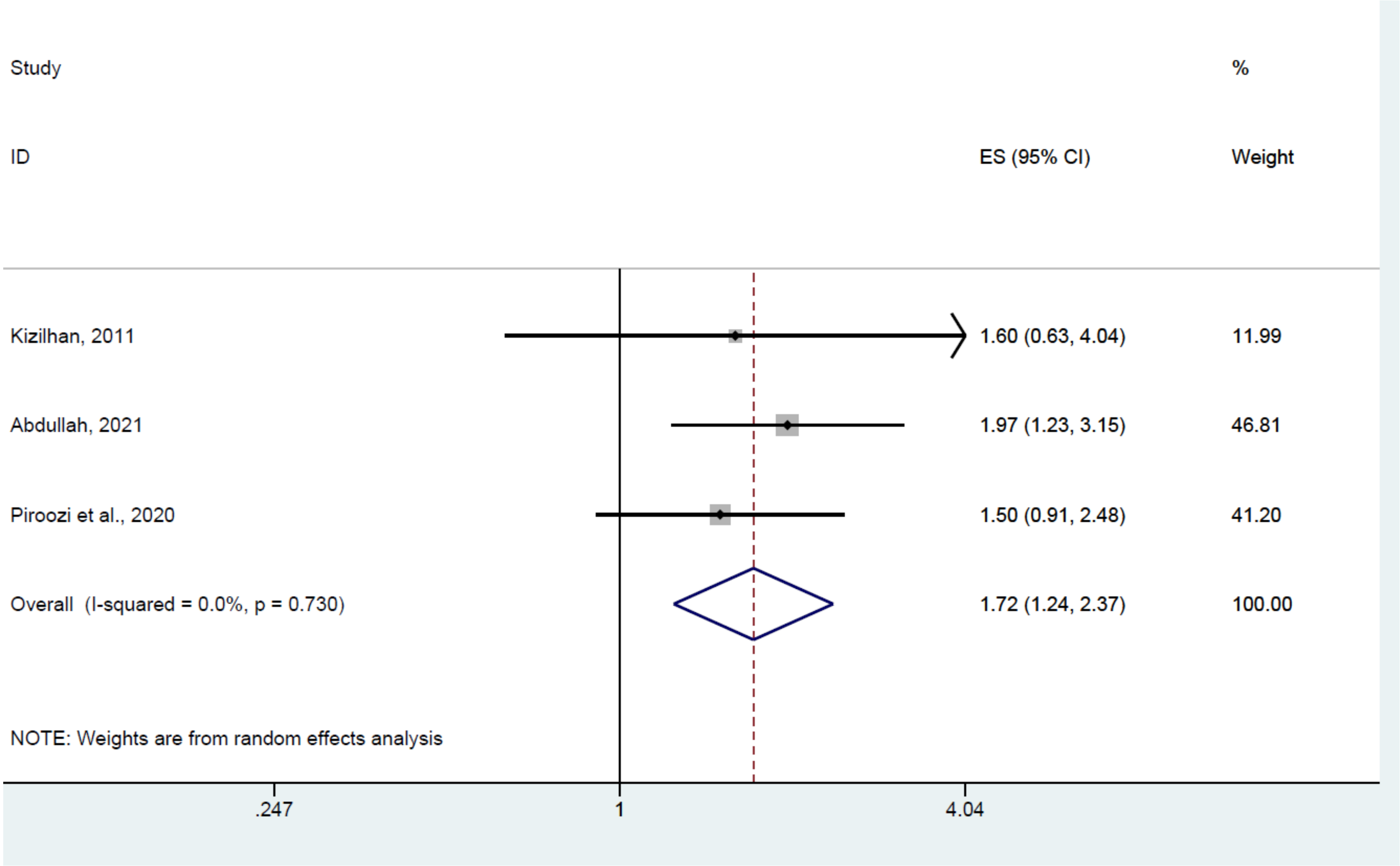

Supplement: Supplementary file 2 — Supplementary Material 2. [file 12889_2025_21584_MOESM2_ESM.pdf]
